# Synthesis of 1,2,3-triazoles containing an allomaltol moiety from substituted pyrano[2,3-*d*]isoxazolones via base-promoted Boulton–Katritzky rearrangement

**DOI:** 10.3762/bjoc.20.117

**Published:** 2024-06-11

**Authors:** Constantine Vyacheslavovich Milyutin, Andrey Nikolaevich Komogortsev, Boris Valerievich Lichitsky

**Affiliations:** 1 N.D. Zelinsky Institute of Organic Chemistry, Russian Academy of Sciences, Leninsky Pr., 47, Moscow, 119991, Russian Federation

**Keywords:** allomaltol, Boulton–Katritzky rearrangement, hydrazones, pyrano[2,3-*d*]isoxazolones, recyclization, 1,2,3-triazoles

## Abstract

For the first time, the interaction of aroyl containing pyrano[2,3-*d*]isoxazolone derivatives with various hydrazines was studied. It was shown that the considered process includes formation of corresponding hydrazones followed by Boulton–Katritzky rearrangement. As a result, the general method for the synthesis of substituted 1,2,3-triazoles bearing an allomaltol fragment was elaborated. The suggested approach can be applied to various aromatic and heterocyclic hydrazines. At the same time for unsubstituted hydrazine the Boulton–Katritzky recyclization is not implemented. In this case the opening of the pyranone ring was observed leading to pyrazolylisoxazole derivatives. Both types of aforementioned structures were proved by X-ray analysis.

## Introduction

**Scheme 1:**
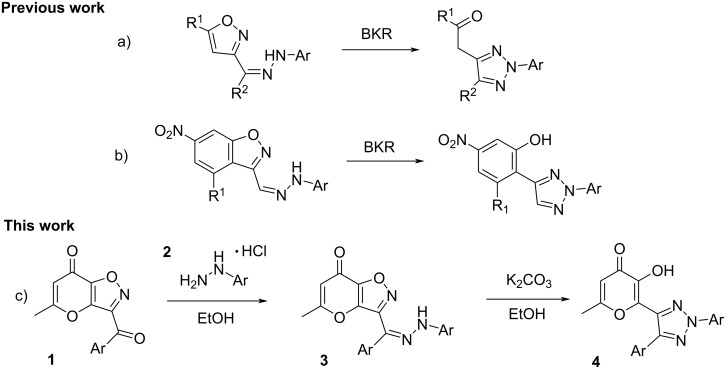
Synthesis of various triazole derivatives using Boulton–Katritzky rearrangement.

The Boulton–Katritzky rearrangement (BKR) also known as mononuclear heterocyclic rearrangement is a valuable recyclization of various systems containing an N–O bond in the ring. This approach is a powerful synthetic tool allowing to obtain diverse types of nitrogen-containing five-membered heterocycles [1–2]. Among the wide variety of recyclizations of this class the processes with participation of hydrazones attract special attention. This reaction is a general method for the preparation of 1,2,3-triazoles bearing various substituents at position 2. Wherein, depending on the type of starting heterocycles various functional derivatives are formed. So, the well-known Boulton–Katritzky reaction of hydrazones of 1,2,4-oxadiazoles leads to the corresponding 1,2,3-triazoles containing an amide fragment. Generally, the considered rearrangement proceeds under action of acidic or basic reagents [3–7]. Other options for this process are based on the application of copper salts or ionic liquids [8–9]. Also, thermal and photochemical variants of studied recyclization are known in the literature [10–11]. Besides that, an unusual procedure for Boulton–Katritzky reaction including the use of surfactants is suggested by Fontana and co-workers [12]. Other heterocyclic systems capable to Boulton–Katritzky rearrangement are furazanes and furoxanes. In the case of furazanes the recyclization leads to 1,2,3-triazoles with an oxime moiety in the side chain [13–14]. At the same time 1,2,3-triazole N-oxides are formed from similar furoxanes [15]. Furthermore, special attention is paid to the considered rearrangement of isoxazoles containing an hydrazone unit. In this case the studied process results in formation of corresponding 1,2,3-triazoles with carbonyl moiety (Scheme 1a) [16–19]. Despite the wide variety of described recyclizations of this type for diverse hydrazones there is only one example of an Boulton–Katritzky rearrangement for a condensed heterocyclic system known in the literature (Scheme 1b) [20]. In this regard extension of this reaction to another annulated products is an actual task.

Previously, the methods for the synthesis of a wide range of terarylenes containing an allomaltol fragment were developed in our group [21–26]. Ongoing the research in this area, herein, we studied the reaction of aroyl containing substituted pyrano[2,3-*d*]isoxazolones **1** with various hydrazine hydrochlorides **2**. It was shown that formed hydrazones **3** undergo base-promoted Boulton–Katritzky rearrangement to appropriate 1,2,3-triazoles **4** (Scheme 1c). Based on the performed investigation a general approach to the preparation of corresponding terarylenes with a 3-hydroxy-4-pyranone unit was designed.

## Results and Discussion

**Scheme 2:**
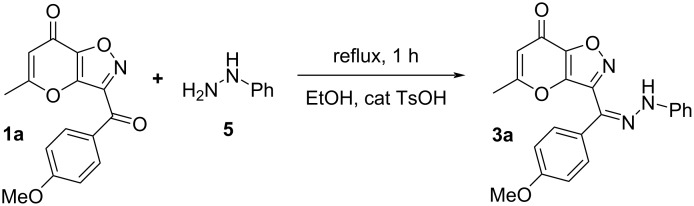
Synthesis of hydrazone **3a**.

**Scheme 3:**
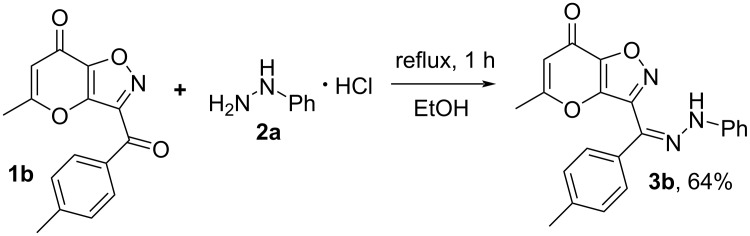
Synthesis of hydrazone **3b** using phenylhydrazine hydrochloride.

The starting ketones **1** were obtained in three steps from allomaltol by a previously described method [27–28] Earlier, we have shown that hydrazone **3a** can be synthesized by reaction of compound **1a** with phenylhydrazine (**5**) in ethanol using a catalytic amount of *p*-TsOH (Scheme 2). Next, we supposed that hydrochlorides of arylhydrazines can be used as starting materials in the studied condensation. We tested this hypothesis using the interaction of ketone **1b** and phenylhydrazine hydrochloride (**2a**). It was shown that reflux of the starting compounds in ethanol for 1 h leads to the target hydrazone **3b** in 64% yield (Scheme 3). It should be noted that based on NMR spectroscopy data the synthesized product **3b** exists as a mixture of *E*/*Z* isomers. Having in hands hydrazone **3** we tried to perform the Boulton–Katritzky rearrangement into corresponding 1,2,3-triazole **4**. In order to achieve the best yields of product **4b** we varied the used reagents, solvents and time of the process. The obtained results are presented in Table 1.

**Table 1:** Optimization of the reaction conditions^a^.

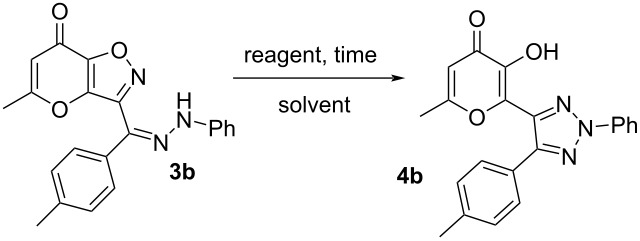				

Entry	Solvent	Reagent	Time, h	Yield, %

1	MeCN	Et_3_N	1	44
2	MeCN	K_2_CO_3_	1	53
3	MeCN	AcONa	1	31
4	MeCN	DABCO	1	42
5	MeCN	DBU	1	–
6	MeCN	NaHCO_3_	1	–
7	DMF	K_2_CO_3_	1	62
8	acetone	K_2_CO_3_	1	54
9	dioxane	K_2_CO_3_	1	49
10	EtOH	K_2_CO_3_	1	71
11	EtOH	K_2_CO_3_	2	77
12	EtOH	K_2_CO_3_	6	76
13	EtOH	–	6	–
14	AcOH	–	6	–
15^b^	EtOH	K_2_CO_3_	2	77
16	EtOH_aq_ (80%)	K_2_CO_3_	2	65

^a^Reaction conditions: **3b** (0.5 mmol, 0.18 g), reagent (1.5 mmol), solvent (5 mL), reflux, air atmosphere; ^b^inert atmosphere.

Initially, we tried to perform the considered rearrangement using various basic reagents at reflux in MeCN for 1 h (Table 1, entries 1–6). Among the tested conditions the best yield was observed in the case of K_2_CO_3_ (Table 1, entry 2). It should be noted that application of strong base DBU led to the complex mixture of products (Table 1, entry 5). At the same time basicity of NaHCO_3_ is not enough for realization of the studied recyclization and in this case the starting hydrazone **3b** was isolated unchanged (Table 1, entry 6). Further, we tested the reaction in various solvents employing K_2_CO_3_ as a base (Table 1, entries 7–10). Wherein, optimal results were achieved utilizing EtOH (Table 1, entry 10). Next, we varied time of the process (Table 1, entries 11 and 12). It was shown that reflux for 2 h leads to slight increase in the yield of product **4b** (Table 1, Entry 11). At the same time further prolongation of the reaction did not affect on the obtained results (Table 1, entry 12). It is important to emphasize that basic reagent is necessary for the considered rearrangement. For example, reflux in EtOH or AcOH for 6 h without any dopants leads only to recovery of the starting material (Table 1, entries 13 and 14). Also, it should be noted that implementation of the studied reaction in inert atmosphere did not influence the yield of target product **4b** (Table 1, entry 15). Besides that, we assessed the influence of water on the considered recyclization. So, carrying out the process in 80% aqueous EtOH resulted in a diminished yield (Table 1, entry 16). Thus, optimal conditions for the investigated recyclization are the application of K_2_CO_3_ in EtOH at reflux for 2 h.

**Scheme 4:**
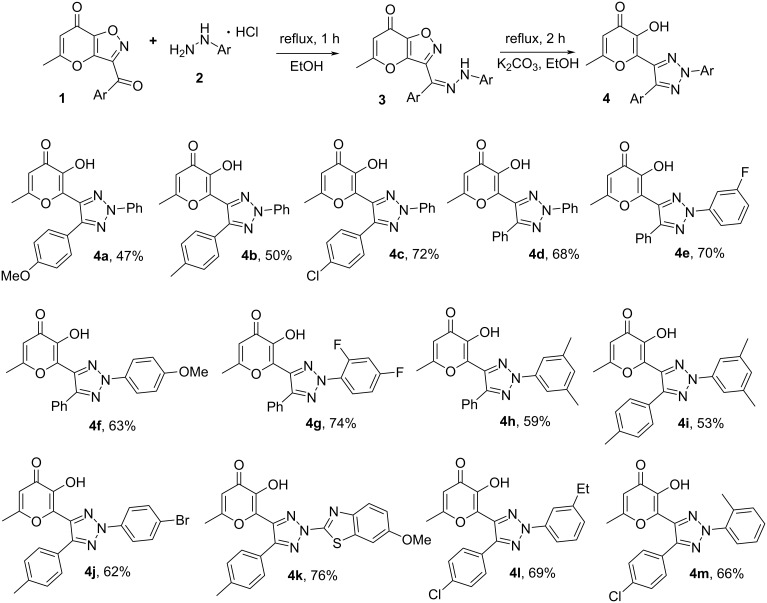
Synthesis of target 1,2,3-triazoles **4**. Reaction conditions: **1** (0.5 mmol), arylhydrazine hydrochloride (0.55 mmol), EtOH (5 ml), then K_2_CO_3_ (1.5 mmol, 0.21 g), EtOH (5 ml).

The elaborated conditions allowed us to prepare an array of target 1,2,3-triazoles **4** with allomaltol unit (Scheme 4). It is interesting to note that the recyclization products can be synthesized in good yields employing various starting arylhydrazones **3** both with donor and acceptor substituents at aromatic rings. Besides that, heterocyclic hydrazones also can be utilized in the considered rearrangement. It should be mentioned that the presented reaction is the first example of a recyclization of the pyrano[2,3-*d*]isoxazolone core.

**Figure 1:**
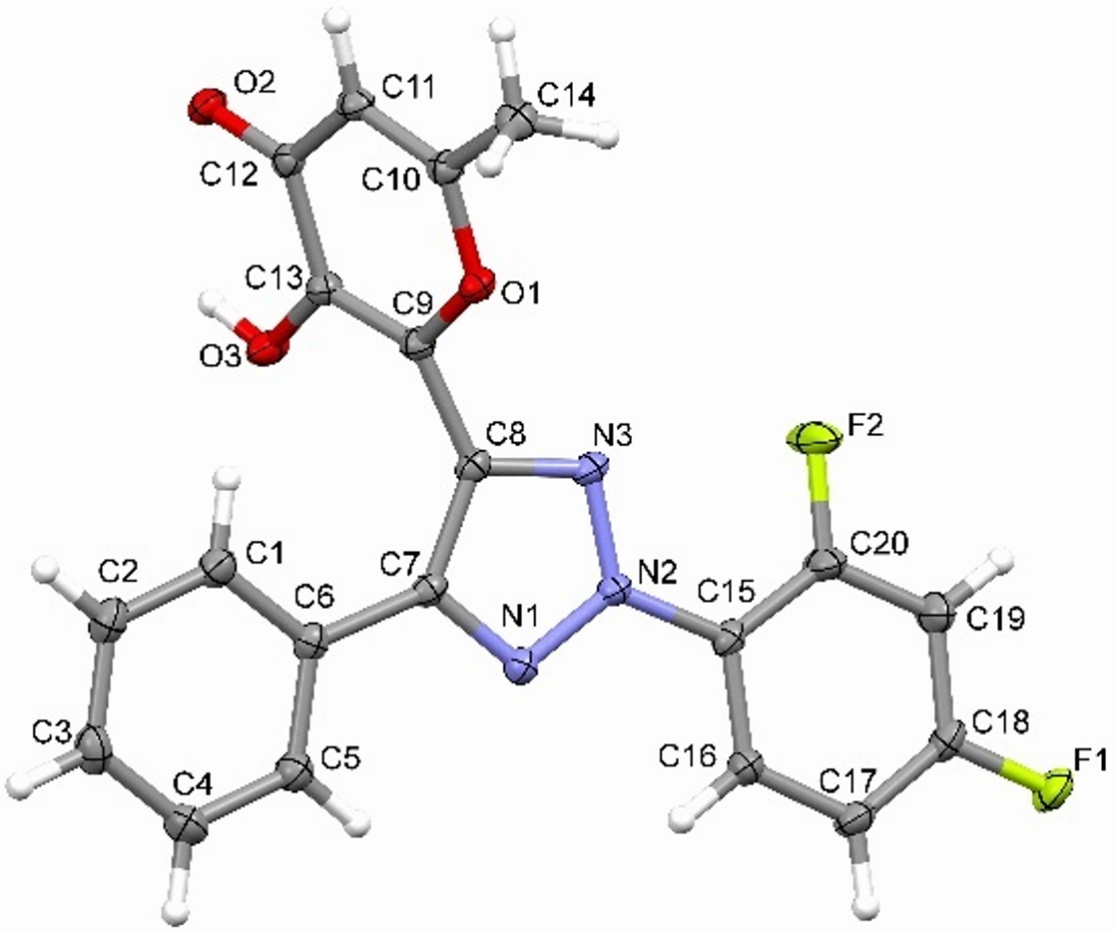
The X-ray crystal structure of compound **4g** (CCDC 2343878).

The obtained 1,2,3-triazoles **4** are solid crystalline products, whose structures were proved by ^1^H, ^13^C NMR spectroscopy and high-resolution mass spectrometry. Moreover, X-ray analysis was used for confirmation of structure of compound **4g** (Figure 1).

**Scheme 5:**
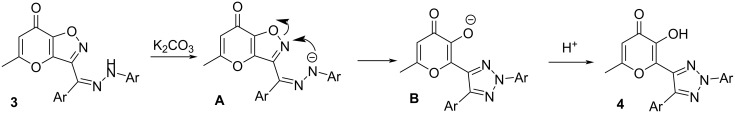
Proposed reaction mechanism.

A plausible mechanism of the studied rearrangement is presented in Scheme 5. At first, anion **A** is generated from starting hydrazone **3** under action of base. Next, intramolecular recyclization accompanied by opening of the isoxazole ring and formation of the N–N bond leads to intermediate **B**. Finally, target 1,2,3-triazole **4** is produced via acidification of anion **B**.

**Scheme 6:**
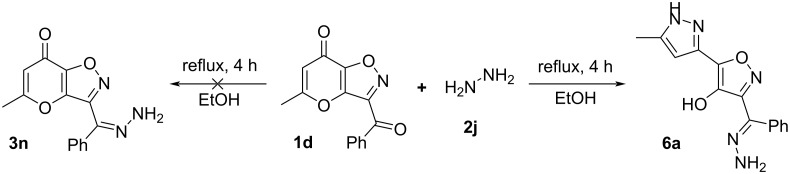
Reaction of **1d** with hydrazine hydrate ^a^.

**Scheme 7:**
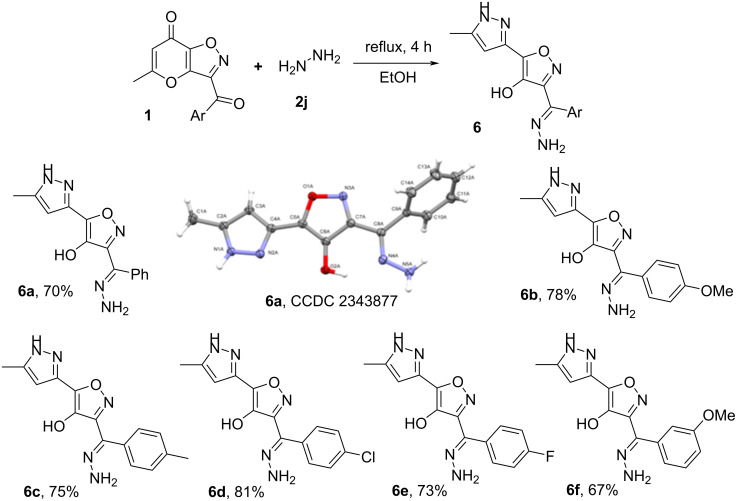
Synthesis of products **6**. Reaction conditions: **1** (0.5 mmol), hydrazine hydrate (1.5 mmol, 0.08 g), EtOH (5 ml).

Next, we tried to expand the presented rearrangement to hydrazones derived from aliphatic hydrazines (MeNHNH_2_, *t-*BuNHNH_2_). Unfortunately, in this case interaction of ketones **1** with aforementioned hydrazines led only to a complex mixture of unidentified products. At the same time the use of benzhydrazide or semicarbazide in the similar condensation resulted in recovery of starting compounds **1**. Further, in order to test the Boulton–Katritzky reaction for unsubstituted hydrazones we investigated the condensation of ketone **1d** with hydrazine. The process was carried out with 3-fold excess of hydrazine hydrate in EtOH at reflux for 4 h. Unexpectedly, in this case instead of desired hydrazone **3n** we have obtained the recyclized product **6a** (Scheme 6), whose structure was confirmed by ^1^H, ^13^C NMR spectroscopy, high-resolution mass spectrometry and X-ray analysis. Based on the aforementioned reaction we have synthesized a set of pyrazolylisoxazoles **6** (Scheme 7).

**Scheme 8:**
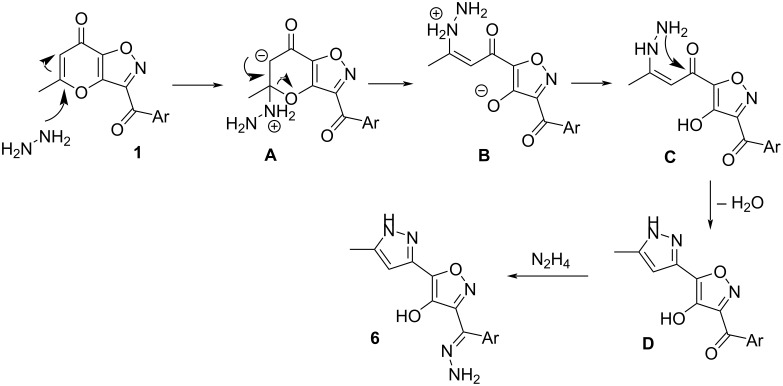
Proposed reaction mechanism for the formation of products **6**.

The proposed mechanism of investigated recyclization is depicted at Scheme 8. Initially, hydrazine molecule is added to double bond of the pyranone ring leading to zwitter-ion **A**. Further, cleavage of dihydropyranone fragment results in intermediate **B**. Next, enehydrazine **C** is formed from compound **B** through migration of a proton. Then, intramolecular cyclization with participation of hydrazine and carbonyl functions leads to pyrazolylisoxazole **D**. Finally, condensation with second equivalent of hydrazine results in the target hydrazone **6**.

**Scheme 9:**
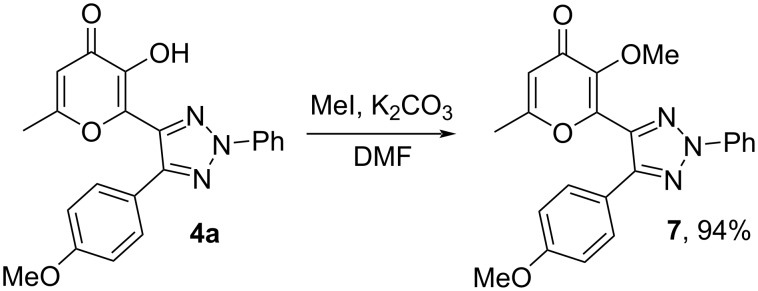
Synthesis of methylated product **7**.

The synthetic utility of obtained 1,2,3-triazoles is demonstrated by its further derivatization. So, alkylation by MeI in the presence of K_2_CO_3_ in DMF allowed us to prepare 3-methoxypyran-4-one derivative **7** (Scheme 9).

## Conclusion

In summary, we investigated the reaction of substituted pyrano[2,3-*d*]isoxazolones with diverse hydrazines. We have demonstrated that initially the studied process leads to appropriate hydrazones. Further, the obtained hydrazones undergo Boulton–Katritzky recyclization to the corresponding 1,2,3-triazoles. Based on the performed study a convenient approach for the preparation of 1,2,3-triazole derivatives with 3-hydroxypyran-4-one unit was designed. It was shown that the proposed method can be used for various aromatic and heterocyclic hydrazines. Wherein, our attempts to realize this protocol for aliphatic hydrazines were unsuccessful. Besides that, in the case of unsubstituted hydrazine opening of the pyranone ring was observed and pyrazolylisoxazoles were produced as a result of recyclization. The structures of one example of the 1,2,3-triazole derivatives and one synthesized pyrazolylisoxazole were established by X-ray analysis.

## Supporting Information

File 1 Experimental procedures, characterization data of all products, copies of ^1^H, ^13^C NMR, spectra of all new compounds, and X-ray crystallographic data.

## Data Availability

All data that supports the findings of this study is available in the published article and/or the supporting information to this article.
