# Supplementary material for: Synthesis of 1,2,3-triazoles containing an allomaltol moiety from substituted pyrano[2,3-d]isoxazolones via base-promoted Boulton–Katritzky rearrangement
Source: Beilstein J Org Chem. 2024 Jun 11;20:1334–40. doi: 10.3762/bjoc.20.117 (PMC11181224; doi:10.3762/bjoc.20.117)
Supplement: File 1 — Experimental procedures, characterization data of all products, copies of 1H, 13C NMR, spectra of all new compounds, and X-ray crystallographic data. [file Beilstein_J_Org_Chem-20-1334-s001.pdf]

BEILSTEIN JOURNAL OF ORGANIC CHEMISTRY

Supporting Information

for

Synthesis of 1,2,3-triazoles containing an allomaltol moiety from substituted pyrano[2,3-*d*]isoxazolones via base-promoted Boulton–Katritzky rearrangement

Constantine V. Milyutin, Andrey N. Komogortsev and Boris V. Lichitsky

Beilstein J. Org. Chem. **2024,** *20,* 1334–1340. doi:10.3762/bjoc.20.117

Experimental procedures, characterization data of all products, copies of ^{1}H, ^{13}C NMR, spectra of all new compounds, and X-ray crystallographic data

License and Terms: This is a supporting information file under the terms of the Creative Commons Attribution License (https://creativecommons.org/licenses/by/4.0). Please note that the reuse, redistribution and reproduction in particular requires that the author(s) and source are credited and that individual graphics may be subject to special legal provisions.
The license is subject to the *Beilstein Journal of Organic Chemistry* terms and conditions: (https://www.beilstein-journals.org/bjoc/terms)

Table of contents

1.General information S2

2.Characterization data of compound 3b S4

3.Characterization data of compounds 4 .... S5

4.Characterization data of compounds 6 .... S8

5.Characterization data of compound 7 .... S10

6.NMR ^{1}H and ^{13}C spectra for starting compounds 3b S11

7.NMR ^{1}H and ^{13}C spectra for starting compounds 4 .... S12

8.NMR ^{1}H and ^{13}C spectra for compounds 6 .... S25

9.NMR ^{1}H and ^{13}C spectra for compound 7 .... S31

10.X-ray crystallographic data and refinement details S32

1. General information

General information. Unless otherwise stated, all starting chemicals were commercially available and were used as received. Arylhydrazines and its hydrochloride salts are toxic materials. For handling arylhydrazines and their hydrochlorides impermeable gloves, lab coat and goggles should be used. The starting compounds **1** were prepared to a procedure described in the literature.[1] NMR spectra were recorded with Bruker AM 300 (300 MHz) and Bruker DRX 500 (500 MHz) spectrometers in DMSO-d_6. Chemical shifts (ppm) are given relative to solvent signals (DMSO-d_6: 2.50 ppm (^{1}H NMR) and 39.52 ppm (^{13}C NMR). High-resolution mass spectra (HRMS) were obtained on a Bruker micrOTOF II instrument using electrospray ionization (ESI). The melting points were determined on a Kofler hot stage. A magnetic stirrer IKA C-MAG HS 7 was used for the reactions that require heating.

General experimental procedure for the synthesis of product 3b.

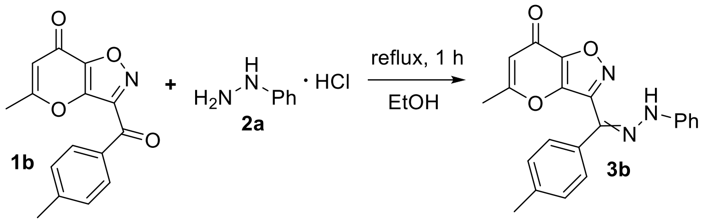

The mixture of **1b** (0.5 mmol, 0.13 g) and phenylhydrazine hydrochloride **2a** (0.55 mmol, 0.08 g) in EtOH (5 ml) was refluxed for 1 h. The resulting precipitate was filtered off and washed with EtOH (3 x 3 ml).

General experimental procedure for the synthesis of product 4b.

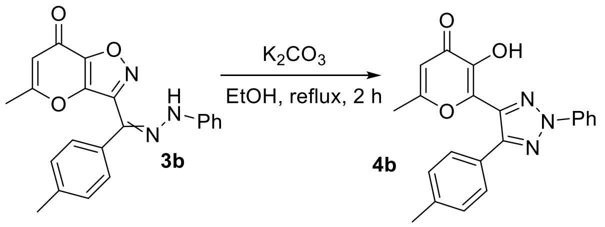

The mixture of hydrazone **3b** (0.5 mmol, 0.18 g) and K_2CO_3 (1.5 mmol, 0.21 g) was refluxed in EtOH (5 ml) for 2 h. After complete of the reaction, AcOH (5 mmol, 0.30 g), H_2O (1 ml) were added to resulting mixture and stirred for 10 min. The precipitated product was filtered off and washed with 50% aqueous EtOH (3 x 3 ml).

General experimental procedure for the synthesis of products 4.

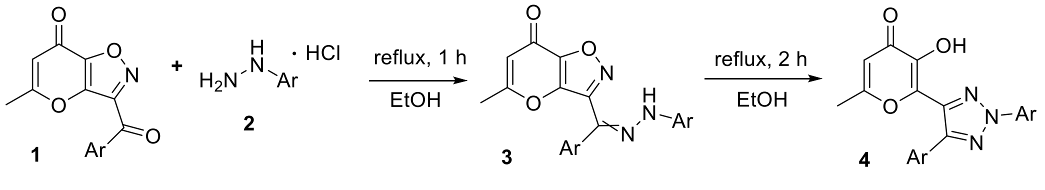

The mixture of **1** (0.5 mmol) and corresponding arylhydrazine hydrochloride (0.55 mmol) in EtOH (5 ml) was refluxed for 1 h. The resulting precipitate was filtered off and washed with EtOH (3x3

[1] Milyutin, C. V.; Komogortsev, A. N.; Melekhina, V. G.; Lichitsky B. V. *Synth. Commun*, **2023**, *53*, 2108-2116. DOI: 10.1080/00397911.2023.2272208

ml). The obtained crude product and K_2CO_3 (1.5 mmol, 0.21 g) was refluxed in EtOH (5 ml) for 2 h. After complete of the reaction, AcOH (5 mmol, 0.30 g), H_2O (1 ml) were added to resulting mixture and stirred for 10 min. The precipitated product was filtered off and washed with 50% aqueous EtOH (3 x 3 ml).

General experimental procedure for the synthesis of products 6.

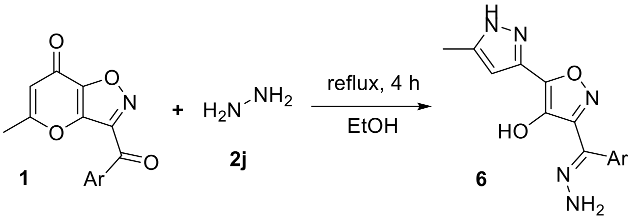

The mixture of **1** (0.5 mmol), hydrazine hydrate (1.5 mmol, 0.08 g) in EtOH (5 ml) was refluxed for 4 h. The resulting precipitate was filtered off and washed with EtOH (3 x 3 ml).

Experimental procedure for the synthesis of 3-methoxy-2-(5-(4-methoxyphenyl)-2-phenyl-2*H*-1,2,3-triazol-4-yl)-6-methyl-4*H*-pyran-4-one (7).

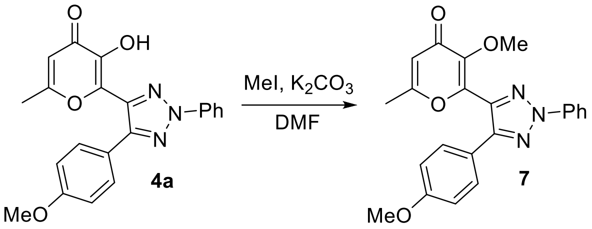

A mixture of compound **4a** (1 mmol, 0.38 g), K_2CO_3 (3 mmol, 0.41 g) and MeI (3 mmol, 0.43 g) in DMF (5 ml) was stirred at room temperature for 8 h. Then the resulting mixture was evaporated in vacuo. To obtained residue, H_2O (50 ml) was added and left overnight. The formed precipitate was filtered off and washed with H_2O (3 × 10 ml).

2. Characterization data of compound 3b

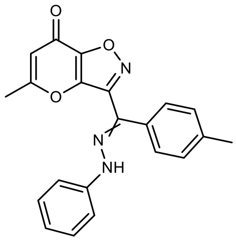

5-Methyl-3-((2-phenylhydrazineylidene)(p-tolyl)methyl)-7*H*-pyrano[2,3-*d*]isoxazol-7-one (**3b**) mixture of E/Z isomers (1:3).

Yellow powder; yield 64% (0.23 g); m.p. 179-181 °C. ^{1}H NMR (300 MHz, DMSO-d_6) δ 10.01 (s, 0.7 H), 9.94 (s, 0.3 H), 7.94 – 7.85 (m, 0.3 H), 7.56 – 7.37 (m, 4H), 7.32 – 7.18 (m, 4.7H), 6.95 – 6.82 (m, 1H), 6.79 (s, 0.3 H), 6.58 (s, 0.7 H), 2.41 (s, 1.8 H), 2.36 – 2.29 (m, 4.2 H). ^{13}C NMR (75 MHz, DMSO-d_6) δ 167.40, 166.53, 152.73, 147.04, 144.79, 144.45, 138.00, 133.23, 130.10, 129.69, 129.52, 129.31, 129.12, 129.05, 129.00, 128.92, 128.87, 127.87, 125.99, 125.36, 124.29, 120.57, 113.79, 113.71, 113.29, 21.32, 20.79, 19.32, 19.15. HRMS (ESI-TOF) *m/z*: $[M+H]^+$ Calcld for $C_{21}H_{17}N_3O_3$: 360.1348; Found 360.1330.

3. Characterization data of compounds 4

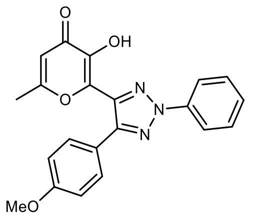

3-Hydroxy-2-(5-(4-methoxyphenyl)-2-phenyl-2H-1,2,3-triazol-4-yl)-6-methyl-4H-pyran-4-one (**4a**)

White powder; yield 47% (0.09 g); m.p. 165-167 °C. ^{1}H NMR (300 MHz, DMSO-d_6) δ 9.52 (s, 1H), 8.10 (d, *J* = 7.9 Hz, 2H), 7.68 – 7.56 (m, 4H), 7.54 – 7.45 (m, 1H), 7.05 (d, *J* = 8.6 Hz, 2H), 6.42 (s, 1H), 3.80 (s, 3H), 2.26 (s, 3H). ^{13}C NMR (126 MHz, DMSO-d_6) δ 173.75, 165.73, 160.06, 147.16, 144.29, 138.79, 138.31, 136.02, 129.93, 128.60, 128.45, 121.50, 118.71, 114.36, 111.82, 55.29, 19.39. HRMS (ESI-TOF) *m/z*: $[M+H]^+$ Calcld for $C_{21}H_{17}N_3O_4$: 376.1297; Found 376.1304.

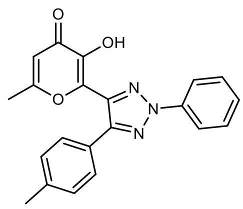

3-Hydroxy-6-methyl-2-(2-phenyl-5-(p-tolyl)-2H-1,2,3-triazol-4-yl)-4H-pyran-4-one (**4b**)

White powder; yield 50% (0.09 g); m.p. 155-157 °C. ^{1}H NMR (300 MHz, DMSO-d_6) δ 9.55 (s, 1H), 8.11 (d, *J* = 7.9 Hz, 2H), 7.69 – 7.54 (m, 4H), 7.53 – 7.44 (m, 1H), 7.29 (d, *J* = 7.8 Hz, 2H), 6.42 (s, 1H), 2.35 (s, 3H), 2.26 (s, 3H). ^{13}C NMR (126 MHz, DMSO-d_6) δ 173.64, 165.65, 147.29, 144.20, 138.88, 138.74, 138.18, 136.29, 129.88, 129.41, 128.47, 127.03, 126.35, 118.73, 111.75, 20.89, 19.31. HRMS (ESI-TOF) *m/z*: $[M+H]^+$ Calcld for $C_{21}H_{17}N_3O_3$: 360.1348; Found 360.1340.

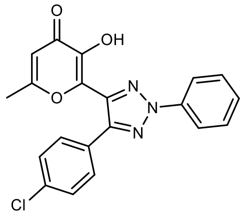

2-(5-(4-Chlorophenyl)-2-phenyl-2H-1,2,3-triazol-4-yl)-3-hydroxy-6-methyl-4H-pyran-4-one (**4c**).

White powder; yield 72% (0.14 g); m.p. 197-199 °C. ^{1}H NMR (300 MHz, DMSO-d_6) δ 9.55 – 8.80 (br. s, 1H), 8.11 (d, *J* = 7.9 Hz, 2H), 7.77 – 7.67 (m, 2H), 7.67 – 7.58 (m, 2H), 7.58 – 7.43 (m, 3H), 6.38 (s, 1H), 2.26 (s, 3H). ^{13}C NMR (126 MHz, DMSO-d_6) δ 173.66, 165.66, 146.13, 144.13, 138.66, 137.84, 136.61, 133.97, 129.89, 129.00, 128.89, 128.64, 128.21, 118.83, 111.77, 19.31. HRMS (ESI-TOF) *m/z*: $[M+H]^+$ Calcld for $C_{20}H_{14}ClN_3O_3$: 380.0802; Found 380.0789.

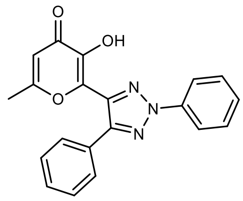

2-(2,5-Diphenyl-2H-1,2,3-triazol-4-yl)-3-hydroxy-6-methyl-4H-pyran-4-one (**4d**).

White powder; yield 68% (0.12 g); m.p. 152-154 °C. ^{1}H NMR (300 MHz, DMSO-d_6) δ 9.57 (s, 1H), 8.12 (d, *J* = 7.9 Hz, 2H), 7.75 – 7.58 (m, 4H), 7.55 – 7.41 (m, 4H), 6.42 (s, 1H), 2.24 (s, 3H). ^{13}C NMR (75 MHz, DMSO-d_6) δ 173.63, 165.59, 147.23, 144.18, 138.71, 138.06, 136.48, 129.86, 129.24, 129.20, 128.79, 128.51, 127.16, 118.76, 111.72, 19.24. HRMS (ESI-TOF) *m/z*: $[M+H]^+$ Calcld for $C_{20}H_{15}N_3O_3$: 346.1192; Found 346.1199.

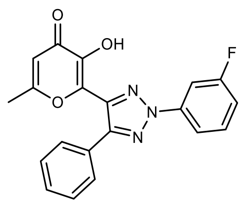

2-(2-(3-Fluorophenyl)-5-phenyl-2H-1,2,3-triazol-4-yl)-3-hydroxy-6-methyl-4H-pyran-4-one (**4e**)

White powder; yield 70% (0.13 g); m.p. 139-141 °C. ^{1}H NMR (300 MHz, DMSO-d_6) δ 9.63 (s, 1H), 8.02 – 7.86 (m, 2H), 7.72 – 7.65 (m, 3H), 7.53 – 7.45 (m, 3H), 7.41 – 7.30 (m, 1H), 6.42 (s, 1H), 2.24 (s, 3H). ^{13}C NMR (75 MHz, DMSO-d_6) δ 173.62, 165.63, 162.44 (d, J_{CF} = 245.4 Hz), 147.61, 144.27, 139.88 (d, J_{CF} = 10.6 Hz), 137.79, 136.96, 131.90 (d, J_{CF} = 9.0 Hz), 129.40, 128.96, 128.80, 127.24, 115.28 (d, J_{CF} = 21.0 Hz),

114.79 (d, J_{CF} = 2.8 Hz), 106.25 (d, J_{CF} = 27.4 Hz), 19.23. HRMS (ESI-TOF) *m/z*: $[M+H]^+$ Calcld for $C_{20}H_{14}FN_3O_3$: 364.1097; Found 364.1088.

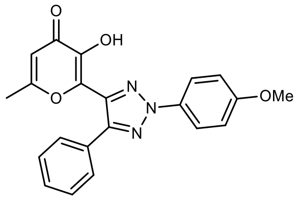

3-Hydroxy-2-(2-(4-methoxyphenyl)-5-phenyl-2H-1,2,3-triazol-4-yl)-6-methyl-4H-pyran-4-one (**4f**)

White powder; yield 63% (0.12 g); m.p. 161-162 °C. ^1H NMR (300 MHz, DMSO-d_6) δ 8.03 (d, *J* = 8.6 Hz, 2H), 7.68 (d, *J* = 6.9 Hz, 2H), 7.51 – 7.42 (m, 3H), 7.16 (d, *J* = 8.6 Hz, 2H), 6.39 (s, 1H), 3.84 (s, 3H), 2.23 (s, 3H). ^{13}C NMR (126 MHz, DMSO-d_6) δ 173.91, 165.41, 159.23, 146.77, 144.51, 138.20, 136.08, 132.39, 129.40, 129.09, 128.77, 127.13, 120.38, 114.87, 111.69, 55.57, 19.27. HRMS (ESI-TOF) *m/z*: $[M+H]^+$ Calcld for $C_{21}H_{17}N_3O_4$: 376.1297; Found 376.1307.

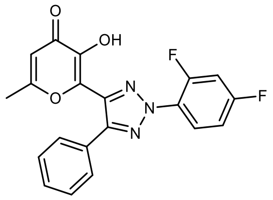

2-(2-(2,4-Difluorophenyl)-5-phenyl-2H-1,2,3-triazol-4-yl)-3-hydroxy-6-methyl-4H-pyran-4-one (**4g**)

White powder; yield 74% (0.14 g); m.p. 155-157 °C. ^1H NMR (300 MHz, DMSO-d_6) δ 9.60 (br. s, 1H), 8.12 – 7.98 (m, 1H), 7.76 – 7.59 (m, 3H), 7.52 – 7.44 (m, 3H), 7.42 – 7.31 (m, 1H), 6.41 (s, 1H), 2.23 (s, 3H). ^{13}C NMR (126 MHz, DMSO-d_6) δ 173.73, 165.71, 162.46 (dd, J_{CF} = 249.9, 11.3 Hz), 154.65 (dd, J_{CF} = 256.3, 13.4 Hz), 147.50, 144.29, 137.37 (d, J_{CF} = 116.2 Hz), 129.39, 129.03, 128.89, 127.45 (d, J_{CF} = 10.5 Hz), 127.25, 124.29 (d, J_{CF} = 3.9 Hz), 124.21 (d, J_{CF} = 3.8 Hz), 112.85 (d, J_{CF} = 3.8 Hz), 112.67 (d, J_{CF} = 3.6 Hz), 111.79, 106.19 (d, J_{CF} = 3.7 Hz), 106.19 (d, J_{CF} = 51.1 Hz), 19.29. HRMS (ESI-TOF) *m/z*: $[M+H]^+$ Calcld for $C_{20}H_{13}F_2N_3O_3$: 382.1003; Found 382.1001.

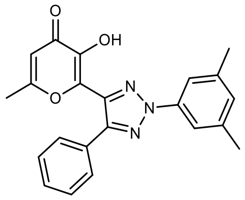

2-(2-(3,5-Dimethylphenyl)-5-phenyl-2H-1,2,3-triazol-4-yl)-3-hydroxy-6-methyl-4H-pyran-4-one (**4h**).

White powder; yield 59% (0.11 g); m.p. 172-174 °C. ^1H NMR (300 MHz, DMSO-d_6) δ 9.53 (br. s, 1H), 7.83 – 7.60 (m, 4H), 7.46 (s, 3H), 7.16 – 7.10 (m, 1H), 6.41 (s, 1H), 2.40 (s, 6H), 2.24 (s, 3H). ^{13}C NMR (126 MHz, DMSO-d_6) δ 173.68, 165.64, 147.02, 144.20, 139.39, 138.70, 138.17, 136.26, 129.93, 129.27, 129.25, 128.82, 127.17, 116.34, 111.76, 20.90, 19.29. HRMS (ESI-TOF) *m/z*: $[M+H]^+$ Calcld for $C_{22}H_{19}N_3O_3$: 374.1505; Found 374.1491.

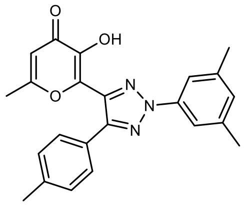

2-(2-(3,5-Dimethylphenyl)-5-(p-tolyl)-2H-1,2,3-triazol-4-yl)-3-hydroxy-6-methyl-4H-pyran-4-one (**4i**)

Yellowish powder; yield 53% (0.10 g); m.p. 151-153 °C. ^1H NMR (300 MHz, DMSO-d_6) δ 7.73 (s, 2H), 7.58 (d, *J* = 7.8 Hz, 2H), 7.28 (d, *J* = 7.8 Hz, 2H), 7.12 (s, 1H), 6.39 (s, 1H), 2.39 (s, 6H), 2.34 (s, 3H), 2.25 (s, 3H). ^{13}C NMR (126 MHz, DMSO-d_6) δ 183.15, 174.01, 165.45, 147.03, 139.35, 138.77, 138.74, 138.19, 136.26, 129.80, 129.38, 127.04, 126.47, 116.26, 111.70, 64.94, 20.91, 19.33, 15.18. HRMS (ESI-TOF) *m/z*: $[M+H]^+$ Calcld for $C_{23}H_{21}N_3O_3$: 388.1661; Found 388.1670.

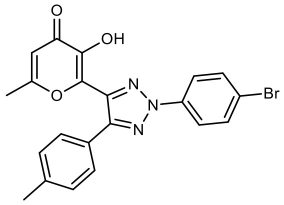

2-(2-(4-Bromophenyl)-5-(p-tolyl)-2H-1,2,3-triazol-4-yl)-3-hydroxy-6-methyl-4H-pyran-4-one (**4j**).

Yellowish powder; yield 62% (0.14 g); m.p. 173-175 °C. ^1H NMR (300 MHz, DMSO-d_6) δ 9.51 (s, 1H), 8.06 (d, *J* = 8.5 Hz, 2H), 7.82 (d, *J* = 8.5 Hz, 2H), 7.57 (d, *J* = 7.8 Hz, 2H), 7.29 (d, *J* = 7.7 Hz, 2H), 6.42 (s, 1H), 2.35 (s, 3H),

2.25 (s, 3H). ^{13}C NMR (126 MHz, DMSO-d_6) δ 165.70, 147.60, 139.05, 137.98, 137.91, 136.70, 132.83, 129.46, 127.11, 126.20, 121.12, 120.69, 111.80, 20.94, 19.34. HRMS (ESI-TOF) *m/z*: $[M+H]^+$ Calcld for $C_{21}H_{16}BrN_3O_3$: 438.0453; Found 438.0450.

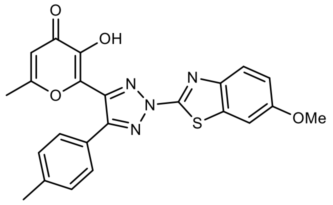

3-Hydroxy-2-(2-(6-methoxybenzo[d]thiazol-2-yl)-5-(p-tolyl)-H-1,2,3-triazol-4-yl)-6-methyl-4H-pyran-4-one (**4k**).

Yellowish powder; yield 76% (0.17 g); m.p. 196-198 °C. ^{1}H NMR (300 MHz, DMSO-d_6) δ 7.95 (d, *J* = 9.0 Hz, 1H), 7.76 (s, 1H), 7.58 (d, *J* = 7.7 Hz, 2H), 7.31 (d, *J* = 7.8 Hz, 2H), 7.23 – 7.13 (m, 1H), 6.44 (s, 1H), 3.85 (s, 3H), 2.35 (s, 3H), 2.25 (s, 3H). ^{13}C NMR (126 MHz, DMSO-d_6) δ 173.67, 165.81, 157.90, 155.17, 148.98, 144.58, 144.46, 139.63, 138.46, 137.14, 134.76, 129.50, 127.36, 125.50, 123.82, 116.42, 111.87, 105.40, 55.81, 20.97, 19.32. HRMS (ESI-TOF) *m/z*: $[M+H]^+$ Calcld for $C_{23}H_{18}N_4O_4S$: 447.1127; Found 447.1120.

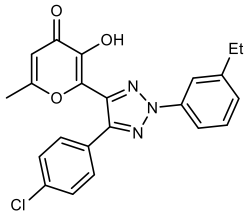

2-(5-(4-Chlorophenyl)-2-(3-ethylphenyl)-2H-1,2,3-triazol-4-yl)-3-hydroxy-6-methyl-4H-pyran-4-one (**4l**).

White powder; yield 69% (0.14 g); m.p. 143-145 °C. ^{1}H NMR (300 MHz, DMSO-d_6) δ 7.98 – 7.88 (m, 2H), 7.76 – 7.67 (m, 2H), 7.59 – 7.47 (m, 3H), 7.35 (d, *J* = 7.6 Hz, 1H), 6.40 (s, 1H), 2.75 (q, *J* = 7.6 Hz, 2H), 2.26 (s, 3H), 1.25 (t, *J* = 7.5 Hz, 3H).. ^{13}C NMR (126 MHz, DMSO-d_6) δ 173.88, 165.67, 146.06, 145.96, 144.42, 138.78, 137.89, 136.63, 133.98, 129.90, 129.05, 128.94, 128.29, 128.19, 118.12, 116.35, 111.82, 28.11, 19.39, 15.54. HRMS (ESI-TOF) *m/z*: $[M+H]^+$ Calcld for $C_{22}H_{18}ClN_3O_3$: 408.1115; Found 408.1111.

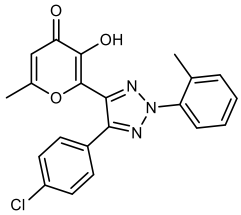

2-(5-(4-Chlorophenyl)-2-(o-tolyl)-2H-1,2,3-triazol-4-yl)-3-hydroxy-6-methyl-4H-pyran-4-one (**4m**).

White powder; yield 66% (0.13 g); m.p. 143-145 °C. ^{1}H NMR (300 MHz, DMSO-d_6) δ 9.55 (s, 1H), 7.75 – 7.64 (m, 3H), 7.59 – 7.39 (m, 5H), 6.41 (s, 1H), 2.40 (s, 3H), 2.25 (s, 3H). ^{13}C NMR (126 MHz, DMSO-d_6) δ 173.88, 165.67, 146.06, 145.96, 144.42, 138.78, 137.89, 136.63, 133.98, 129.90, 129.05, 128.94, 128.29, 128.19, 118.12, 116.35, 111.82, 28.11, 19.39, 15.54. HRMS (ESI-TOF) *m/z*: $[M+H]^+$ Calcld for $C_{21}H_{16}ClN_3O_3$: 394.0958; Found 394.0970.

4. Characterization data of compounds 6

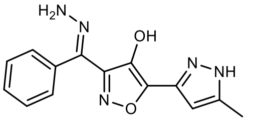

(E)-3-(Hydrazineylidene(phenyl)methyl)-5-(5-methyl-1H-pyrazol-3-yl)isoxazol-4-ol (**6a**)

Yellowish powder; yield 70% (0.10 g); m.p. 201-203 °C. ^{1}H NMR (300 MHz, DMSO-d_6) δ 12.89 (s, 1H), 9.11 (s, 1H), 7.61 – 7.42 (m, 5H), 7.26 (s, 2H), 6.39 (s, 1H), 2.29 (s, 3H).. ^{13}C NMR (126 MHz, DMSO-d_6) δ 151.33, 137.13, 134.16, 129.58, 129.53, 129.45, 129.34, 128.95, 128.68, 127.47, 101.91, 10.54. HRMS (ESI-TOF) *m/z*: [M+H]$^+$ Calcld for $C_{14}H_{13}N_5O_2$: 284.1147; Found 284.1152.

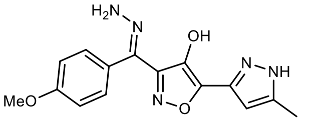

(E)-3-(Hydrazineylidene(4-methoxyphenyl)methyl)-5-(5-methyl-1H-pyrazol-3-yl)isoxazol-4-ol (**6b**)

Yellow powder; yield 78% (0.12 g); m.p. 163-165 °C. ^{1}H NMR (300 MHz, DMSO-d_6) δ 12.88 (s, 1H), 9.18 (s, 1H), 7.43 (d, *J* = 8.3 Hz, 2H), 7.22 – 7.16 (m, 2H), 7.09 (d, *J* = 8.3 Hz, 2H), 6.38 (s, 1H), 3.82 (s, 3H), 2.29 (s, 3H). ^{13}C NMR (126 MHz, DMSO-d_6) δ 159.78, 151.39, 134.27, 134.12, 130.24, 121.52, 114.32, 101.89, 55.26, 10.39. HRMS (ESI-TOF) *m/z*: [M+H]$^+$ Calcld for $C_{15}H_{15}N_5O_3$: 314.1253; Found 314.1260.

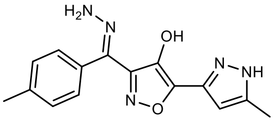

(E)-3-(Hydrazineylidene(p-tolyl)methyl)-5-(5-methyl-1H-pyrazol-3-yl)isoxazol-4-ol (**6c**)

White powder; yield 75% (0.11 g); m.p. 151-153 °C. ^{1}H NMR (300 MHz, DMSO-d_6) δ 12.90 (s, 1H), 9.16 (s, 1H), 7.45 – 7.30 (m, 4H), 7.22 (s, 2H), 6.39 (s, 1H), 2.37 (s, 3H), 2.29 (s, 3H). ^{13}C NMR (126 MHz, DMSO-d_6) δ 151.35, 138.88, 137.40, 134.22, 129.96, 129.50, 128.59, 127.37, 126.56, 101.91, 21.04, 10.60. HRMS (ESI-TOF) *m/z*: [M+H]$^+$ Calcld for $C_{15}H_{15}N_5O_2$: 298.1304; Found 298.1292.

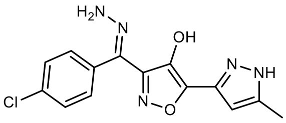

(E)-3-((4-Chlorophenyl)(hydrazineylidene)methyl)-5-(5-methyl-1H-pyrazol-3-yl)isoxazol-4-ol (**6d**)

Yellowish powder; yield 81% (0.13 g); m.p. 125-127 °C. ^{1}H NMR (300 MHz, DMSO-d_6) δ 12.88 (s, 1H), 9.00 (s, 1H), 7.60 (d, *J* = 8.1 Hz, 2H), 7.50 (d, *J* = 8.1 Hz, 2H), 7.38 (s, 2H), 6.38 (s, 1H), 2.29 (s, 3H). ^{13}C NMR (126 MHz, DMSO-d_6) δ 151.31, 135.48, 134.01, 133.91, 130.82, 129.56, 129.50, 129.04, 128.53, 101.93, 10.60. HRMS (ESI-TOF) *m/z*: [M+H]$^+$ Calcld for $C_{14}H_{12}ClN_5O_2$: 318.0758; Found 318.0742.

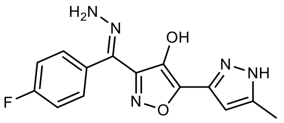

(E)-3-((4-Fluorophenyl)(hydrazineylidene)methyl)-5-(5-methyl-1H-pyrazol-3-yl)isoxazol-4-ol (**6e**)

Brown powder; yield 73% (0.11 g); m.p. 178-180 °C. ^{1}H NMR (300 MHz, DMSO-d_6) δ 12.91 (s, 1H), 9.09 (s, 1H), 7.64 – 7.50 (m, 2H), 7.45 – 7.31 (m, 4H), 6.40 (s, 1H), 2.31 (s, 3H). ^{13}C NMR (75 MHz, DMSO-d_6) δ 162.30 (d, J_{CF} = 246.3 Hz), 151.36, 139.44, 136.04, 134.05, 131.23 (d, J_{CF} = 8.6 Hz), 125.98 (d, J_{CF} = 3.1 Hz), 115.91 (d, J_{CF} = 21.6 Hz), 101.86, 10.25. HRMS (ESI-TOF) *m/z*: [M+H]$^+$ Calcld for $C_{14}H_{12}FN_5O_2$: 302.1053; Found 302.1051.

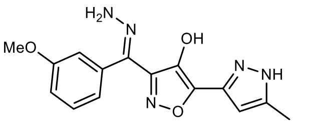

(E)-3-(Hydrazineylidene(3-methoxyphenyl)methyl)-5-(5-methyl-1H-pyrazol-3-yl)isoxazol-4-ol (**6f**)

Pale brown powder; yield 67% (0.10 g); m.p. 170-172 °C. ^{1}H NMR (300 MHz, DMSO-d_6) δ 12.87 (s, 1H), 9.08 (s, 1H), 7.52 – 7.41 (m, 1H), 7.28 (s, 2H), 7.09 – 6.98 (m, 3H),

6.38 (s, 1H), 3.80 (s, 3H), 2.29 (s, 3H). ^{13}C NMR (126 MHz, DMSO-d_6) δ 159.53, 151.27, 139.56, 136.78, 134.16, 130.75, 130.21, 120.78, 115.08, 113.99, 101.91, 55.20, 10.39. HRMS (ESI-TOF) *m/z*: $[M+H]^+$ Calcld for $C_{15}H_{15}N_5O_3$: 314.1253; Found 314.1260.

5. Characterization data of compound 7

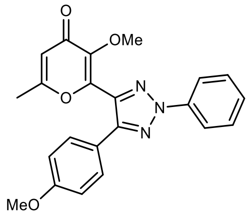

3-Methoxy-2-(5-(4-methoxyphenyl)-2-phenyl-2H-1,2,3-triazol-4-yl)-6-methyl-4H-pyran-4-one (**7**)

Yellowish powder; yield 94% (0.37 g); m.p. 118-120 °C. ^{1}H NMR (300 MHz, DMSO-d_6) δ 8.11 (d, *J* = 7.9 Hz, 2H), 7.68 – 7.56 (m, 4H), 7.56 – 7.47 (m, 1H), 7.07 (d, *J* = 8.3 Hz, 2H), 6.43 (s, 1H), 3.80 (s, 3H), 3.59 (s, 3H), 2.25 (s, 3H). ^{13}C NMR (126 MHz, DMSO-d_6) δ 174.75, 165.76, 160.22, 147.60, 147.48, 145.65, 138.68, 135.58, 129.94, 128.85, 128.59, 121.13, 118.75, 114.90, 114.42, 59.88, 55.29, 19.09. HRMS (ESI-TOF) *m/z*: [M+H]$^+$ Calcld for $C_{22}H_{19}N_3O_4$: 390.1454; Found 390.1443.

6. NMR ^{1}H and ^{13}C spectra for starting compounds 3b

^{1}H NMR spectrum (300 MHz) of **3b** in DMSO-d_6

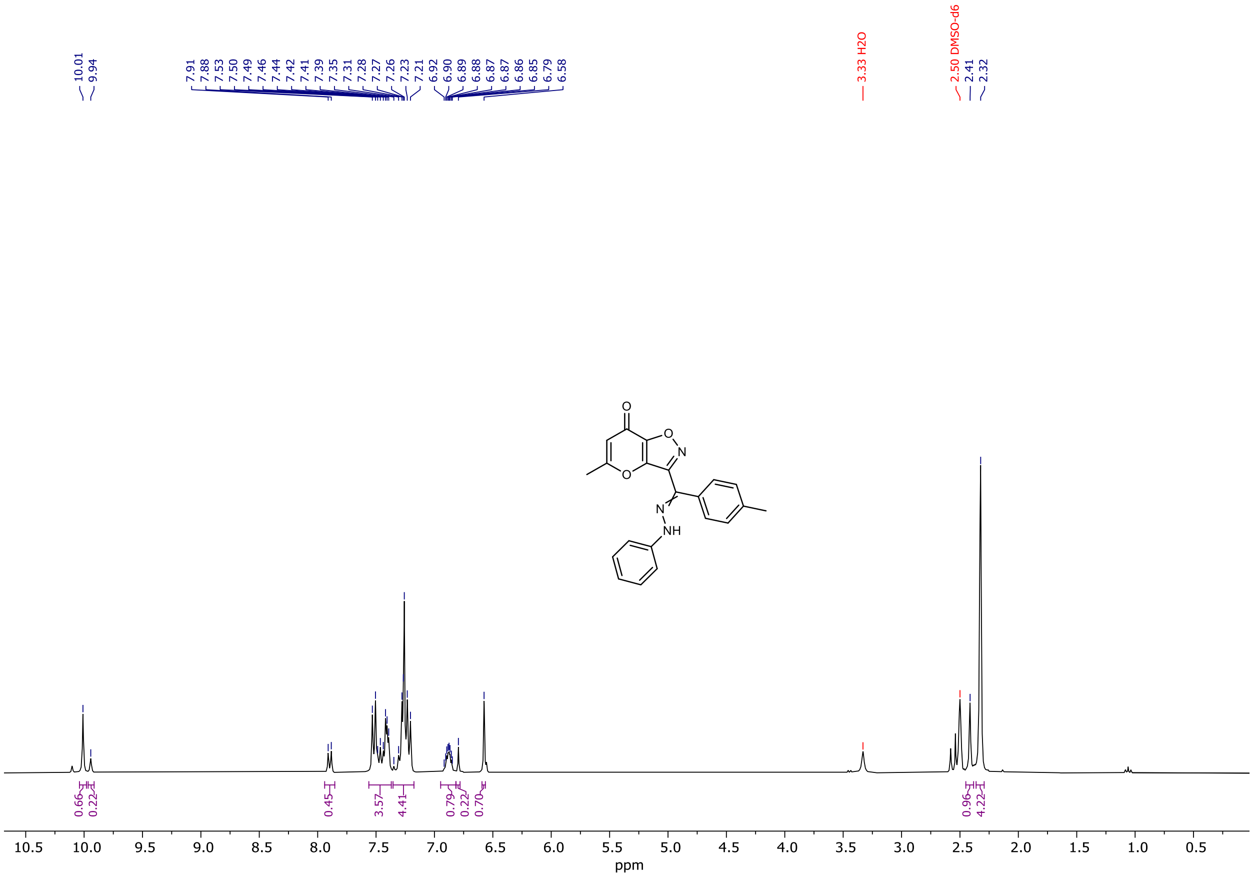

^{13}C {^{1}H} NMR spectrum (75 MHz) of **3b** in DMSO-d_6

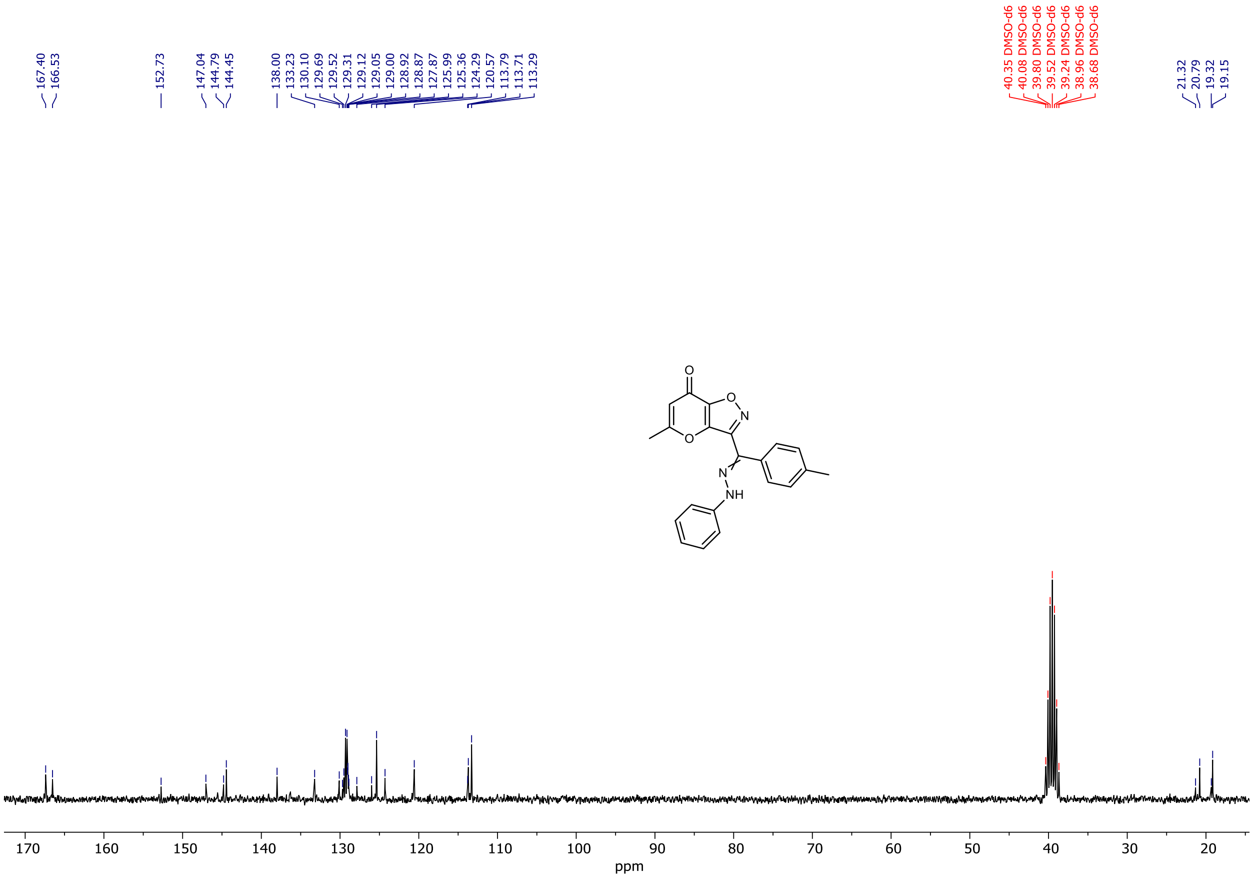

7. NMR ^{1}H and ^{13}C spectra for starting compounds 4

^{1}H NMR spectrum (300 MHz) of **4a** in DMSO-d_6

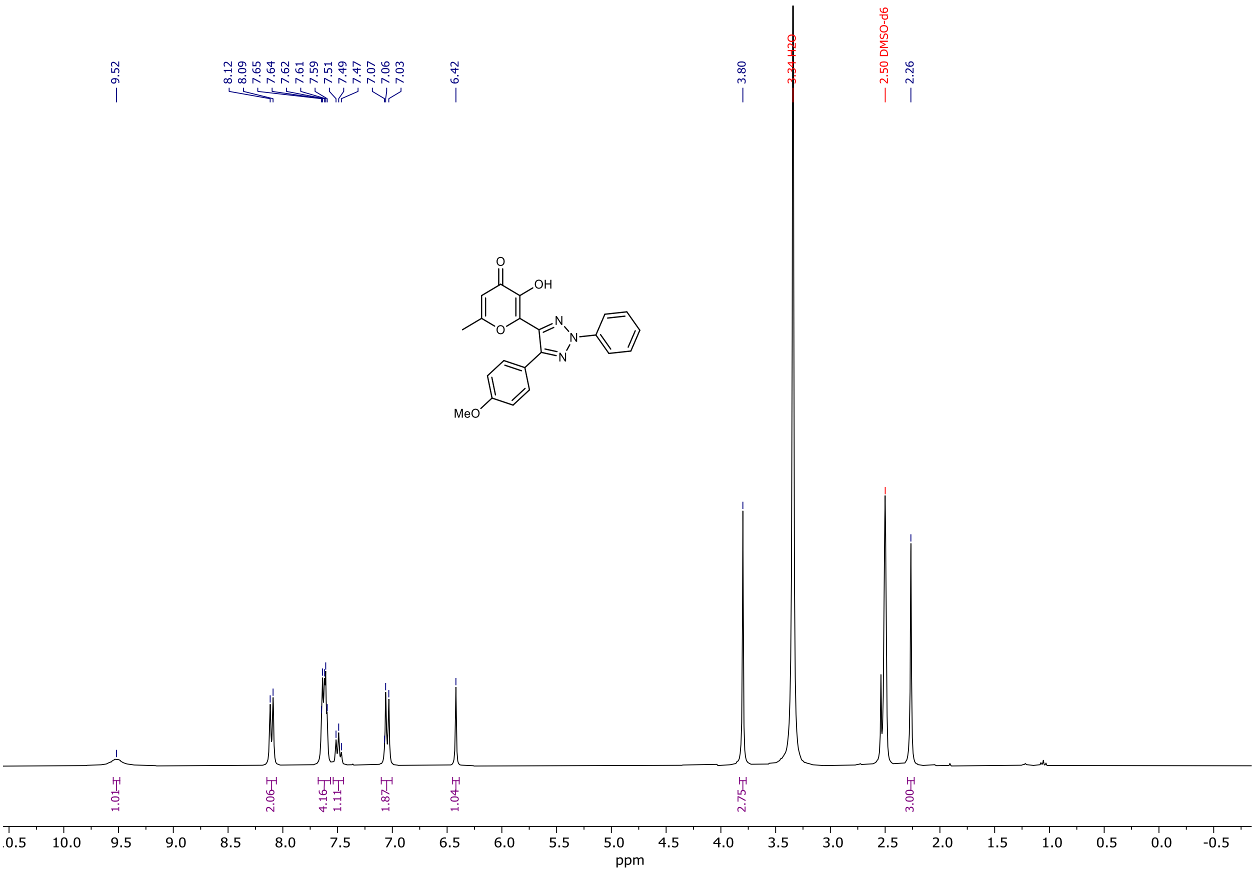

^{13}C {^{1}H} NMR spectrum (126 MHz) of **4a** in DMSO-d_6

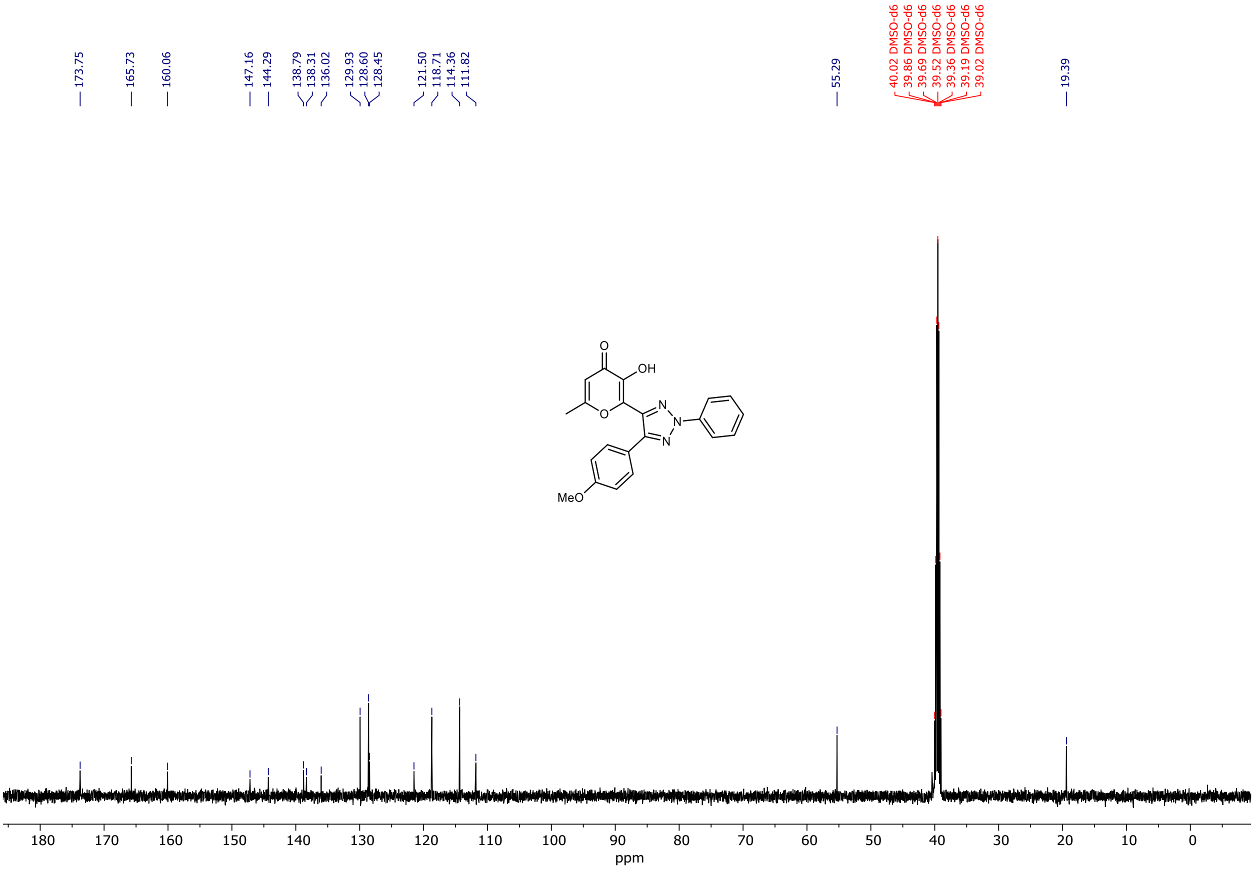

^1H NMR spectrum (300 MHz) of **4b** in DMSO-d_6

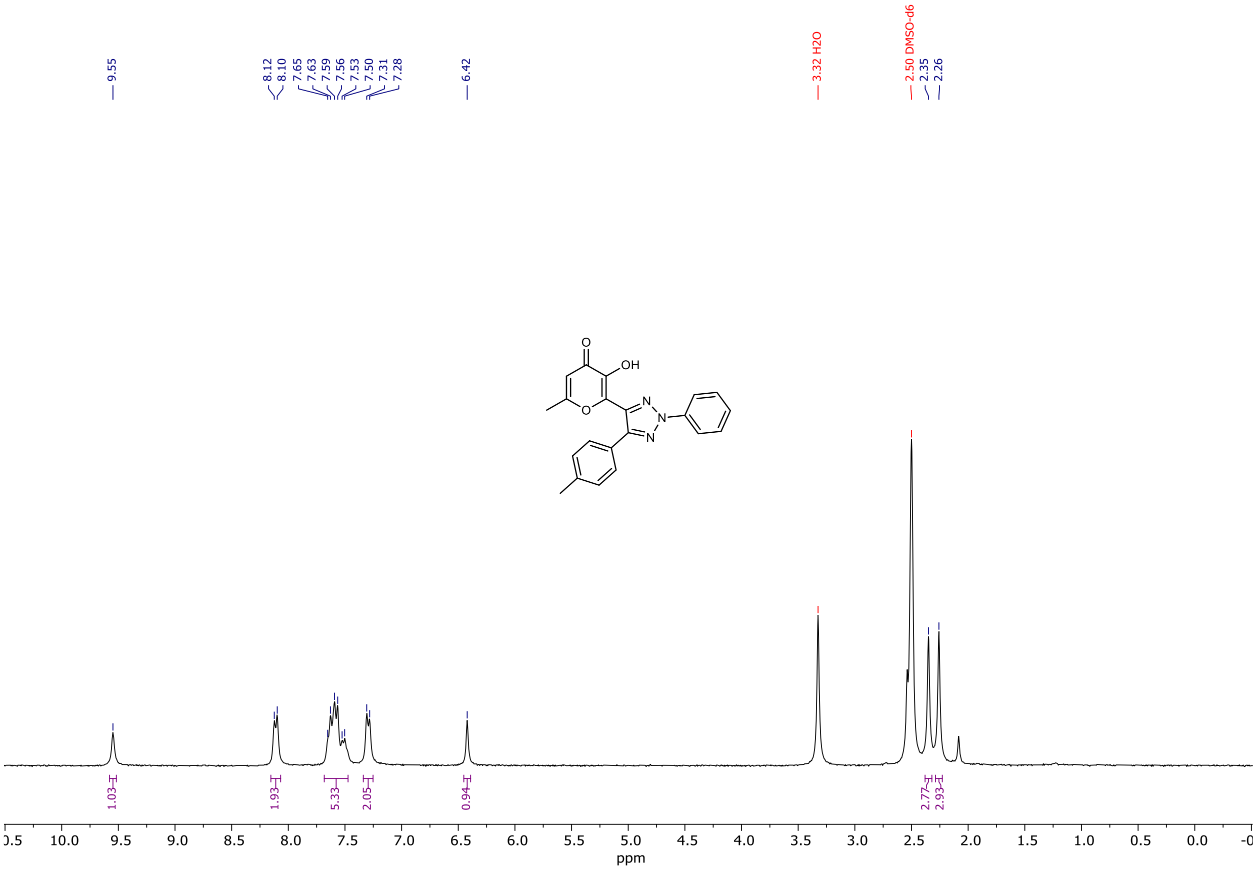

^{13}C {^1H} NMR spectrum (126 MHz) of **4b** in DMSO-d_6

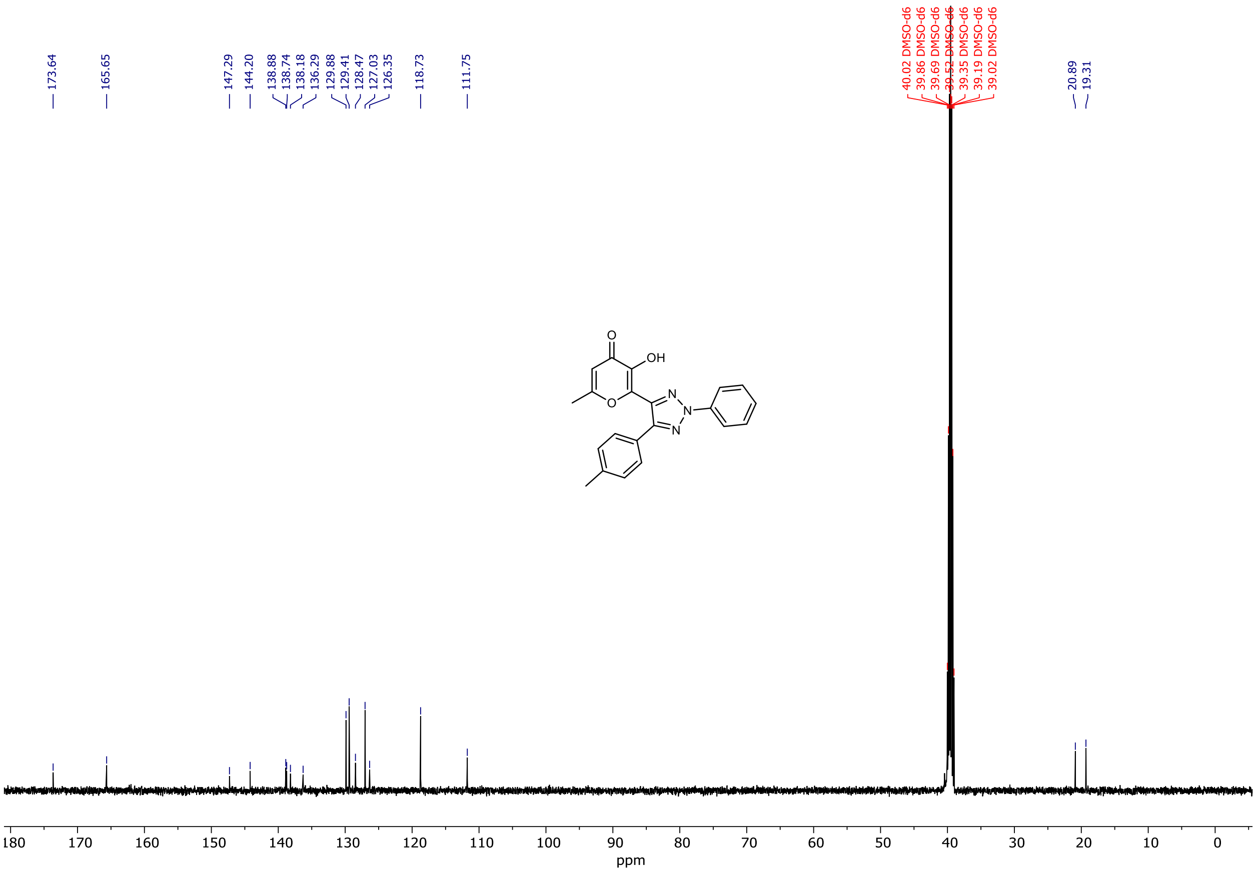

^{1}H NMR spectrum (300 MHz) of **4c** in DMSO-d_6

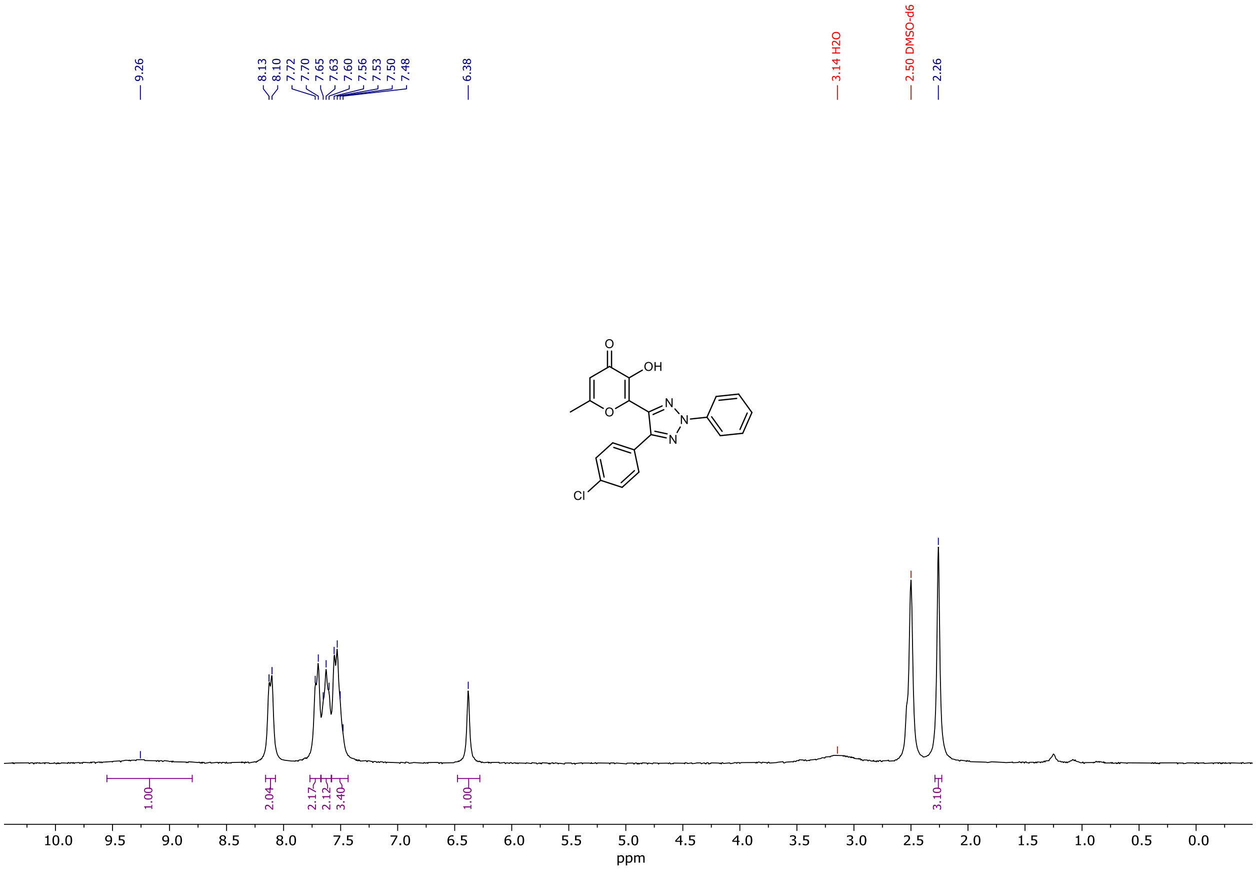

^{13}C {^{1}H} NMR spectrum (126 MHz) of **4c** in DMSO-d_6

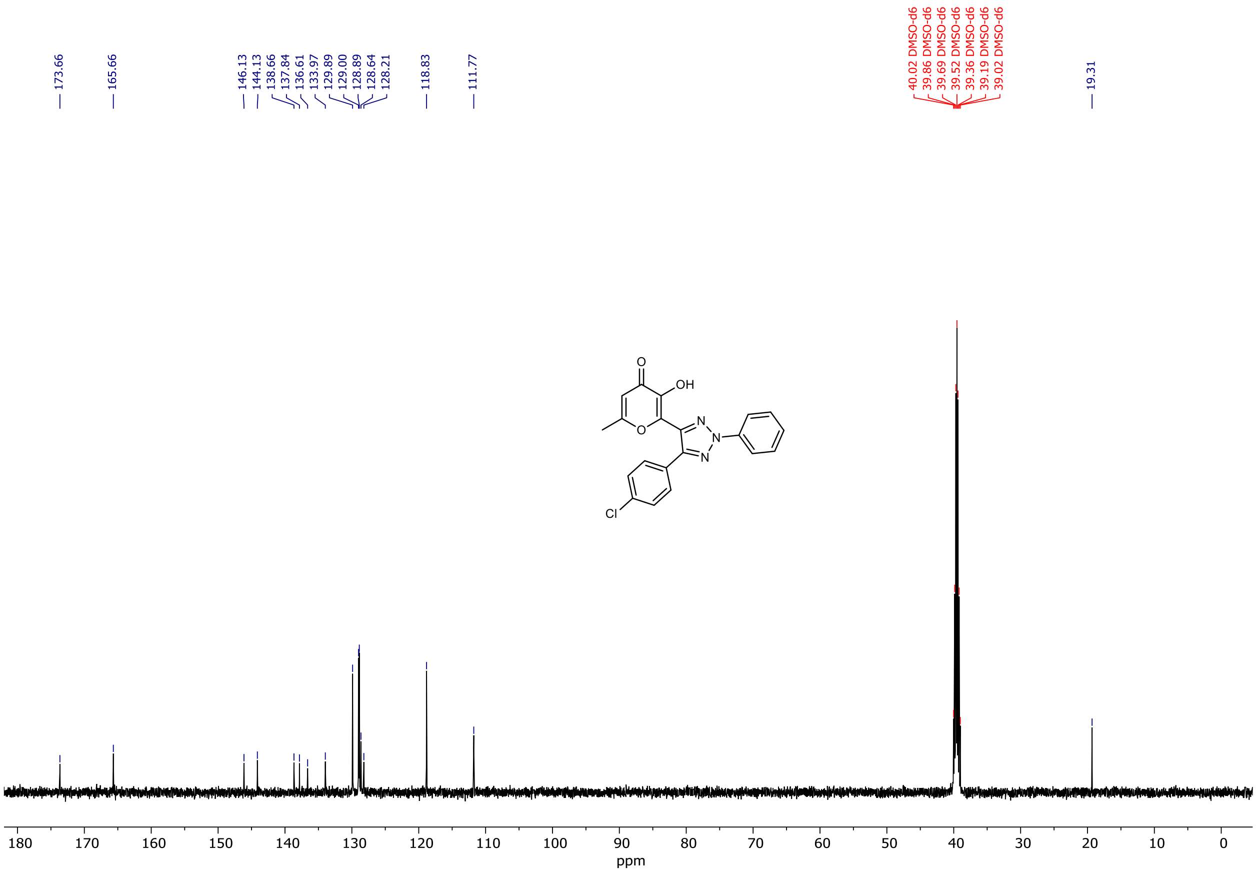

^{1}H NMR spectrum (300 MHz) of **4d** in DMSO-d_6

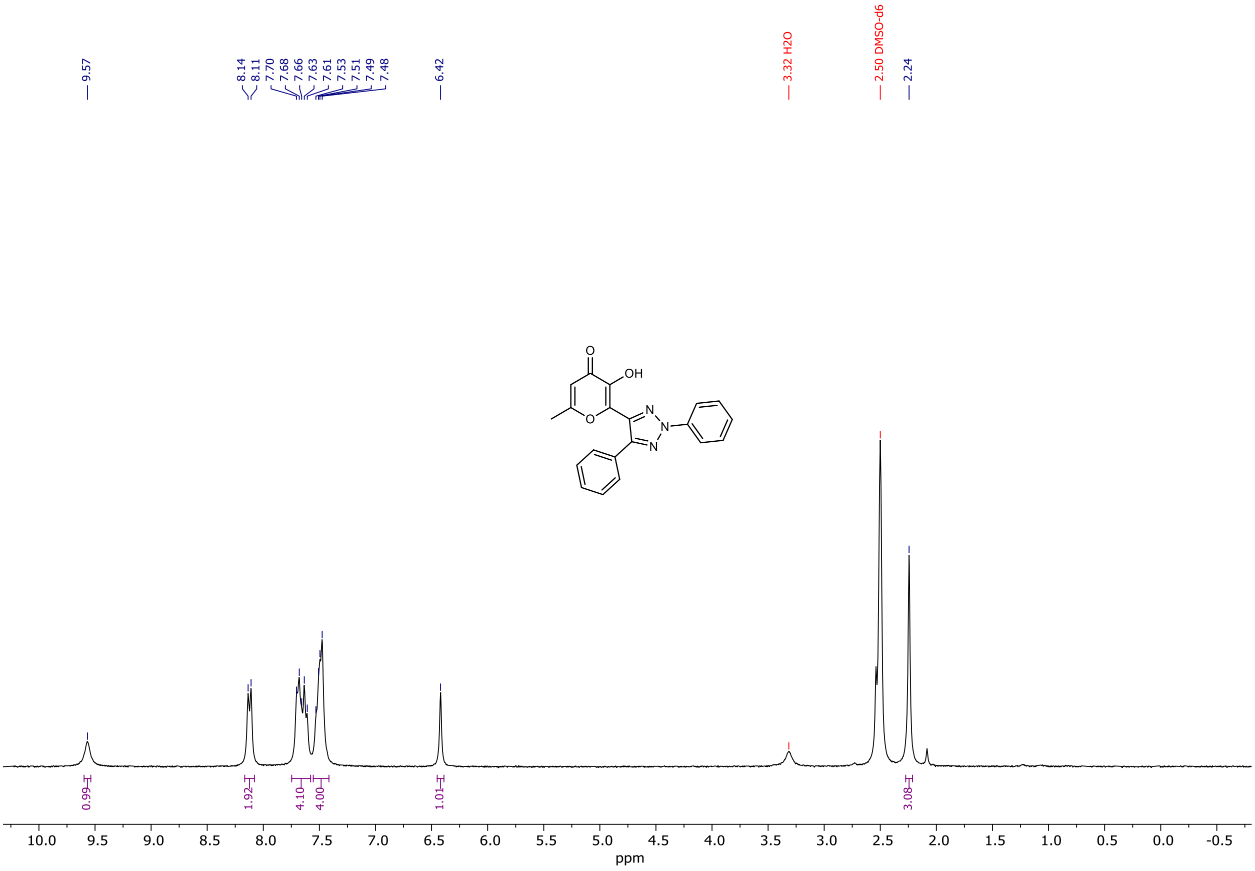

^{13}C {^{1}H} NMR spectrum (75 MHz) of **4d** in DMSO-d_6

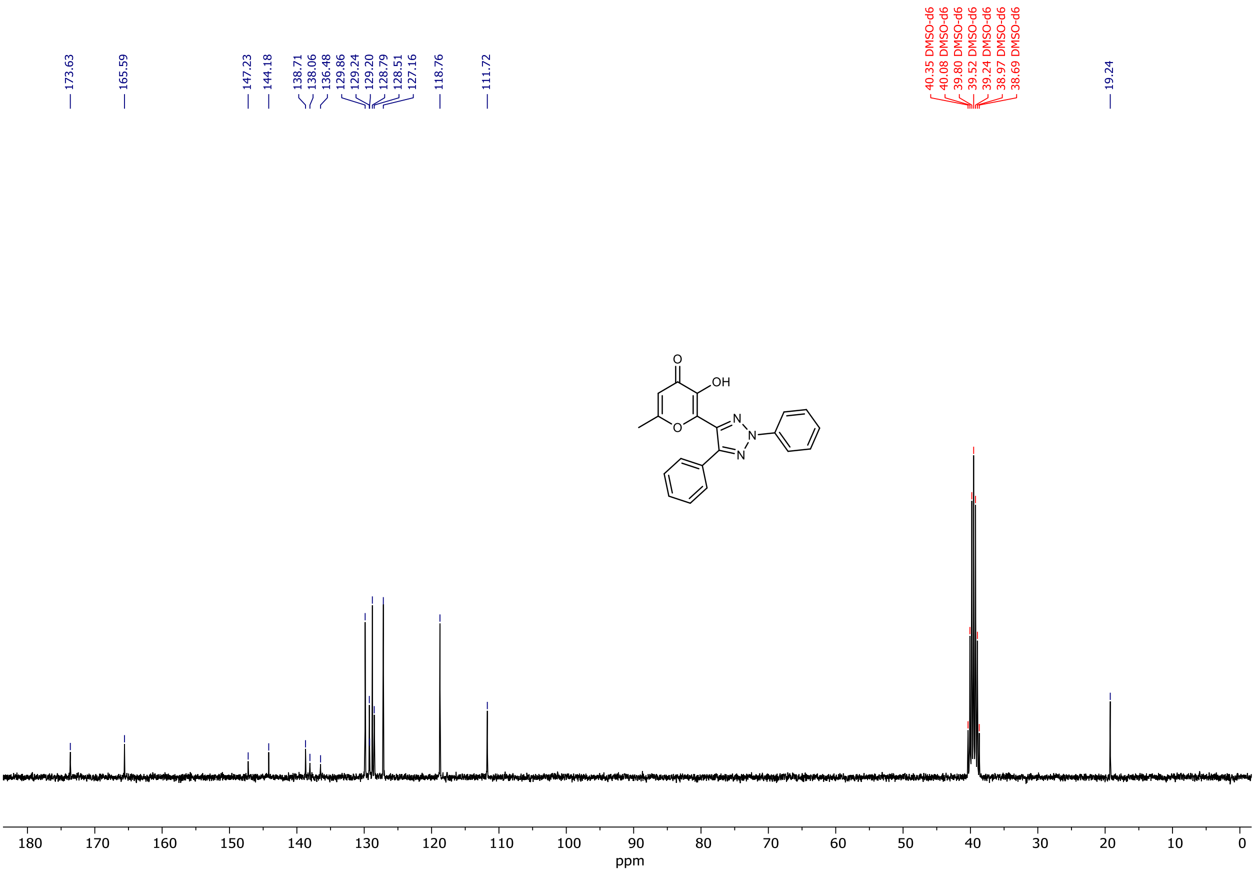

^{1}H NMR spectrum (300 MHz) of **4e** in DMSO-d_6

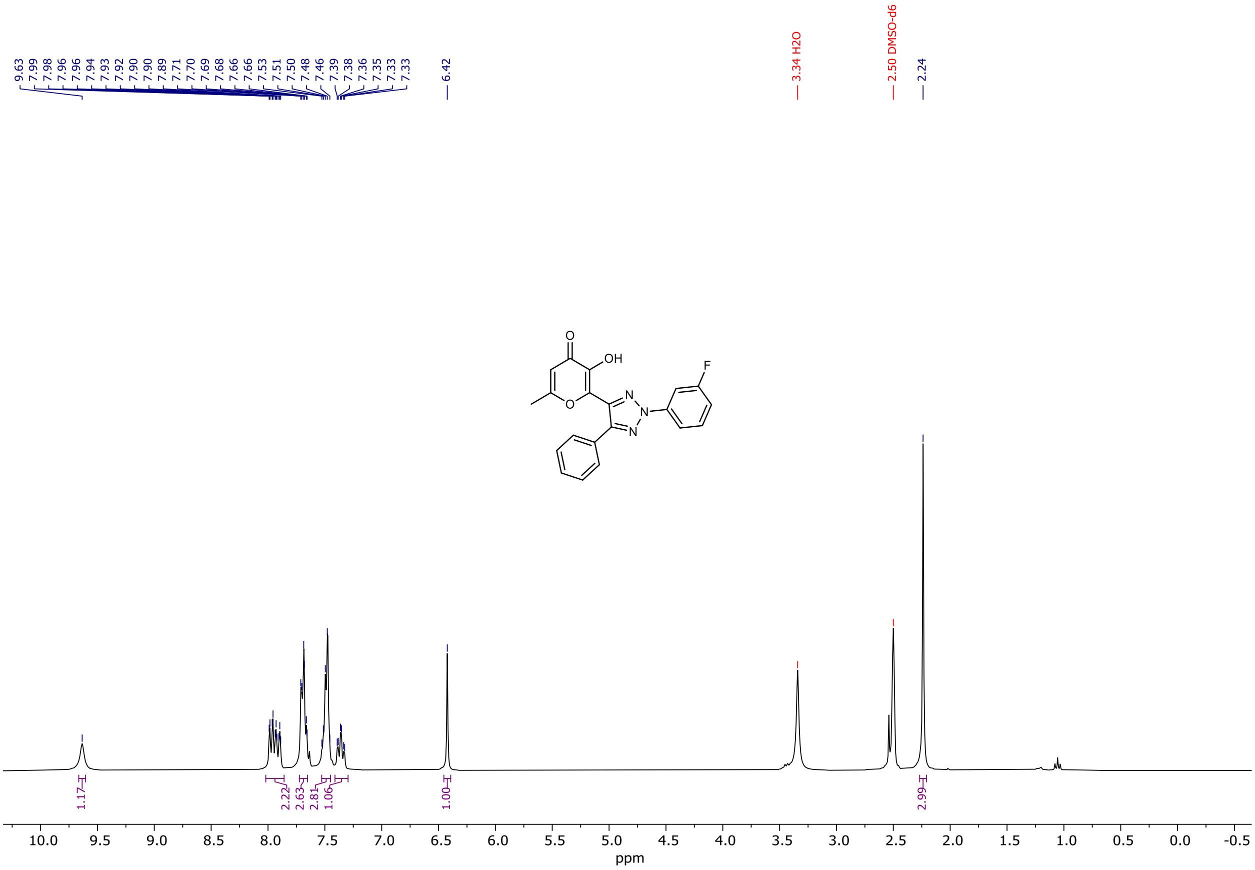

^{13}C {^{1}H} NMR spectrum (75 MHz) of **4e** in DMSO-d_6

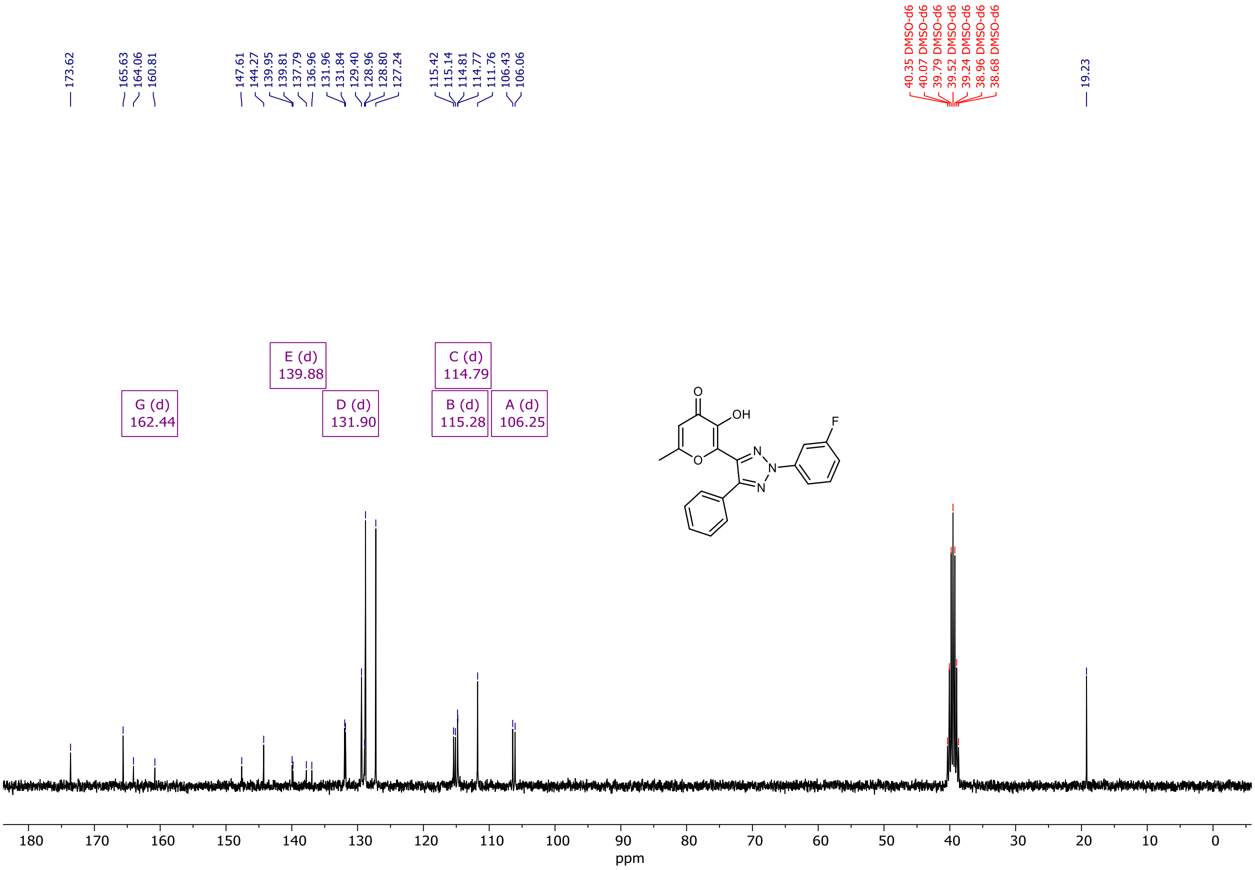

^{1}H NMR spectrum (300 MHz) of **4f** in DMSO-d_6

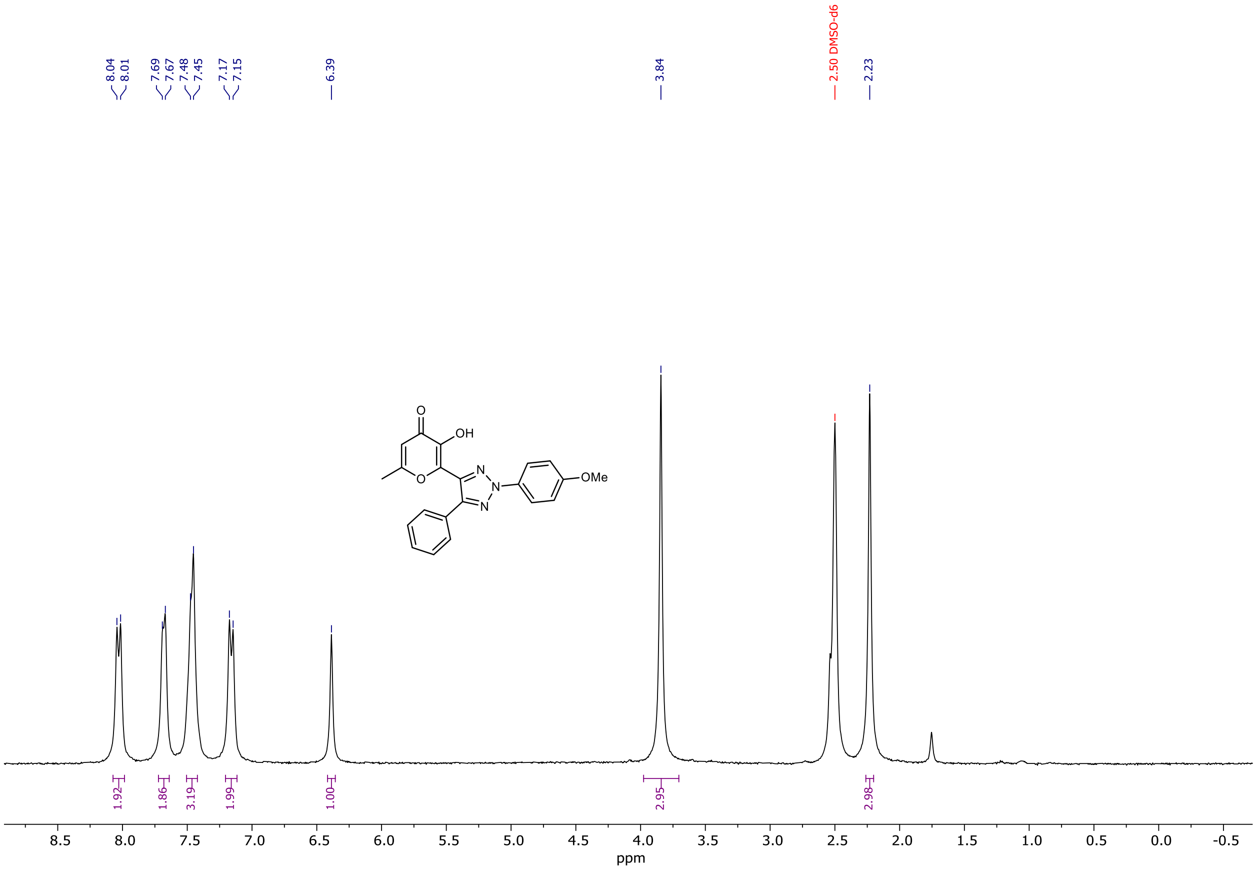

^{13}C {^{1}H} NMR spectrum (126 MHz) of **4f** in DMSO-d_6

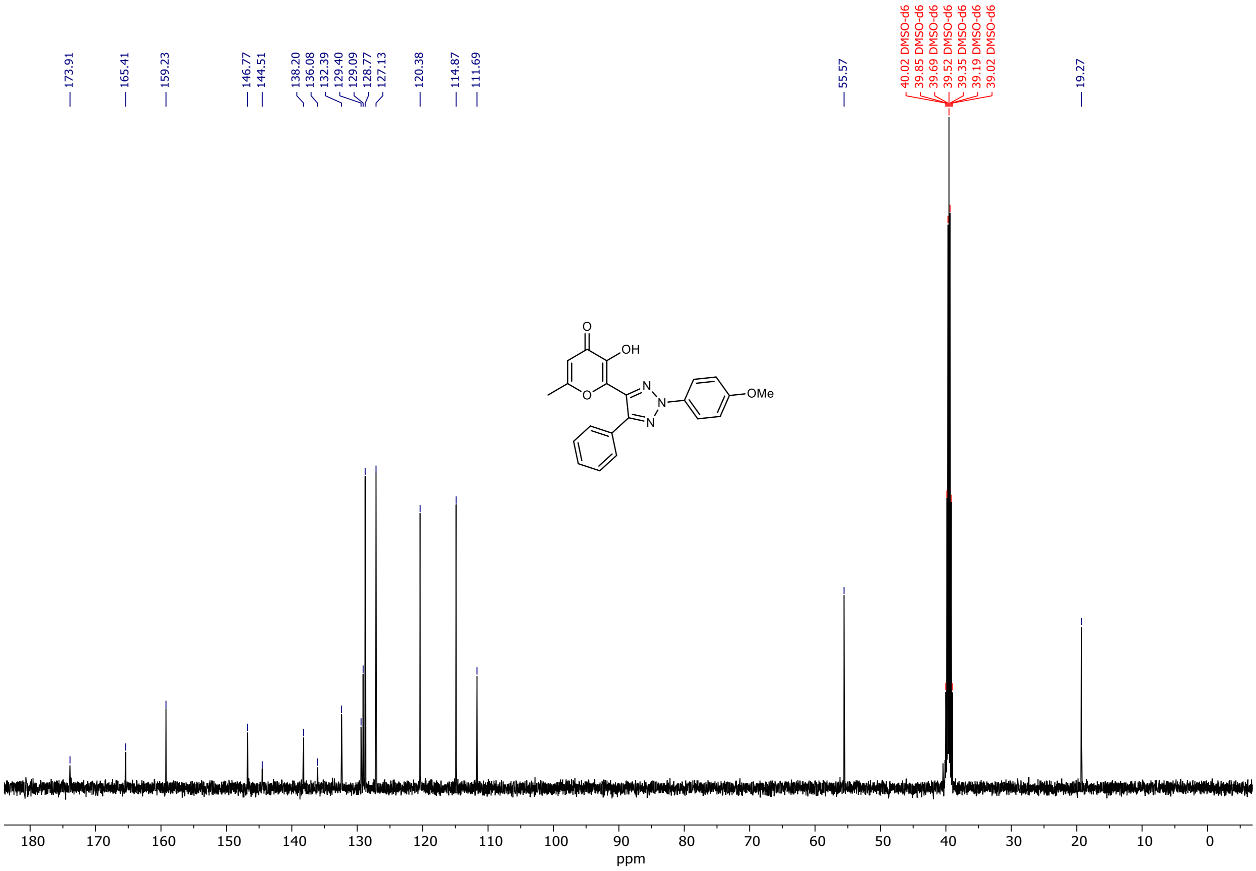

^{1}H NMR spectrum (300 MHz) of **4g** in DMSO-d_6

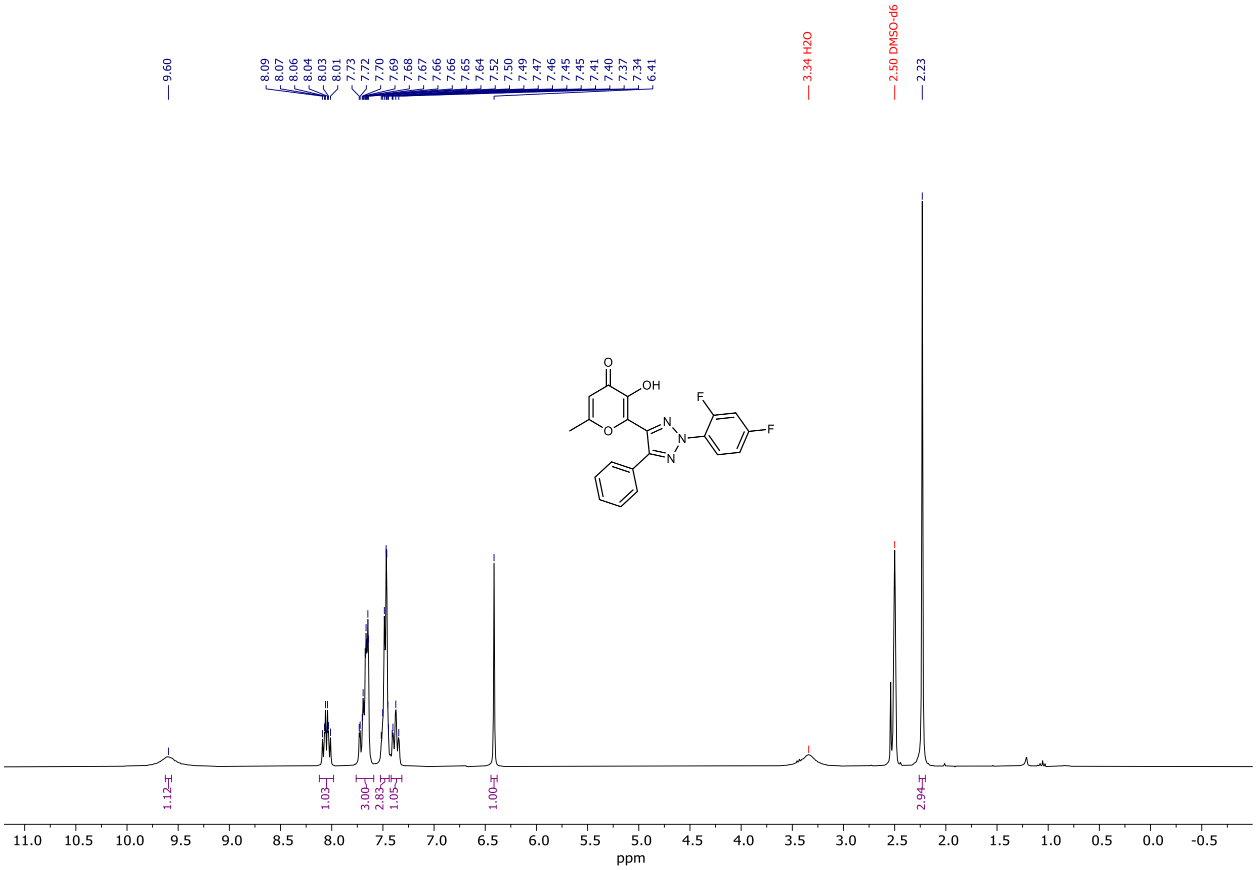

^{13}C {^{1}H} NMR spectrum (126 MHz) of **4g** in DMSO-d_6

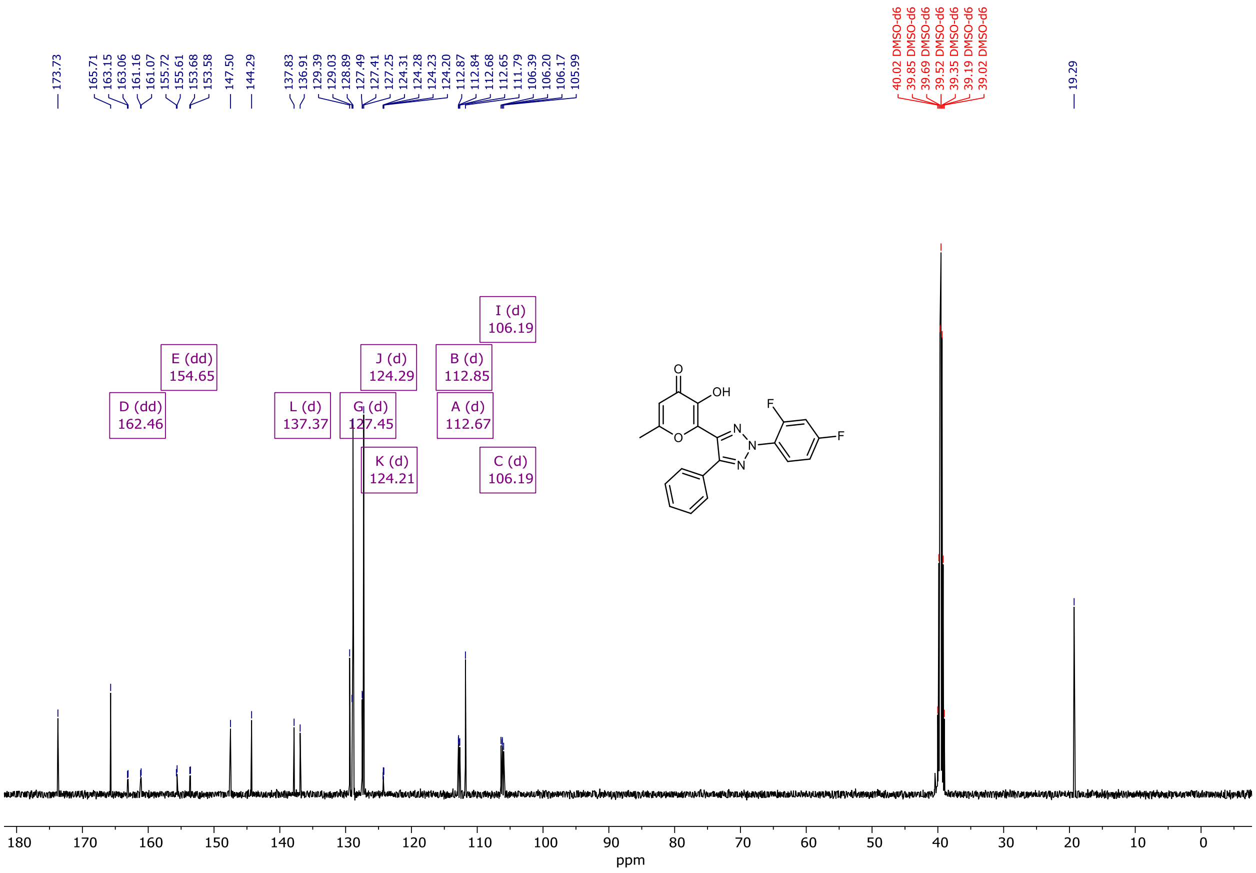

^{1}H NMR spectrum (300 MHz) of **4h** in DMSO-d_6

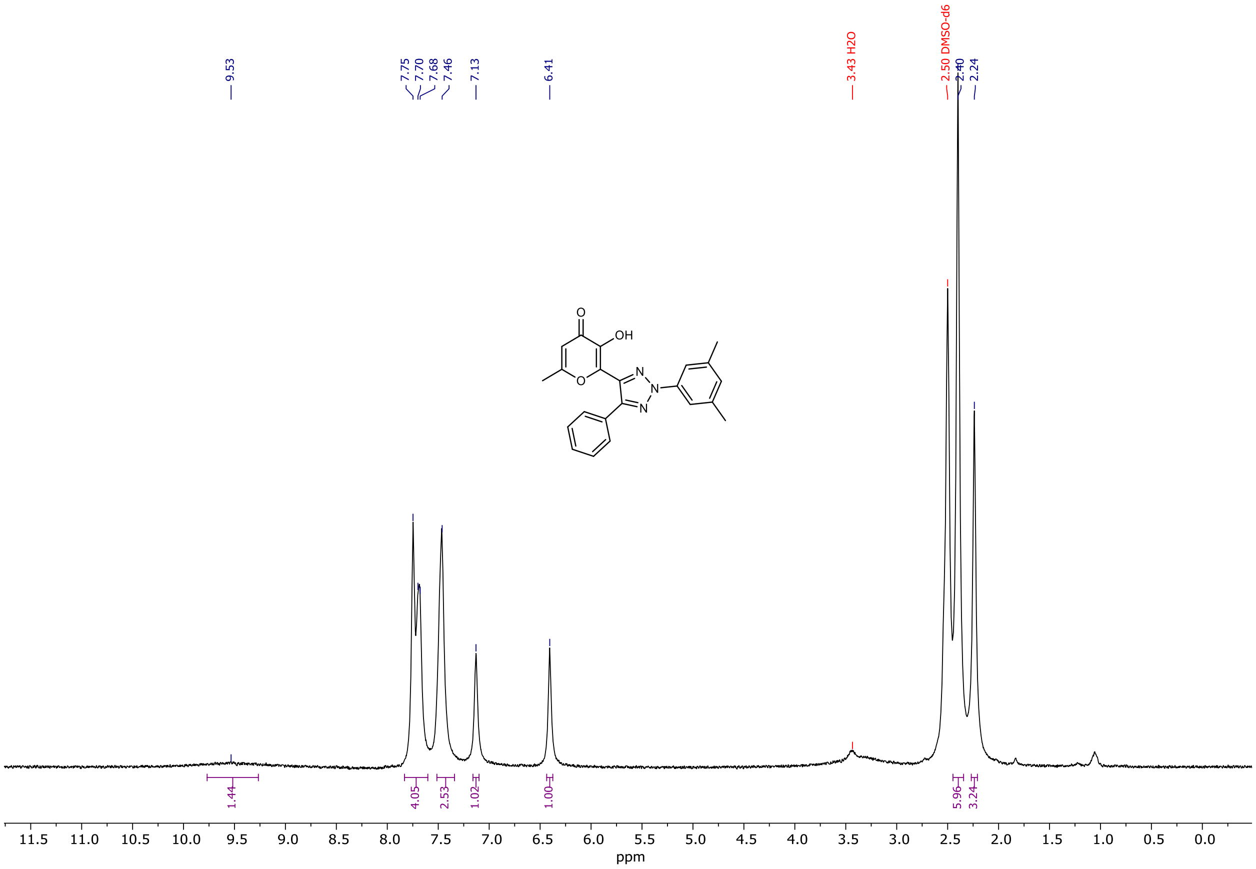

^{13}C {^{1}H} NMR spectrum (126 MHz) of **4h** in DMSO-d_6

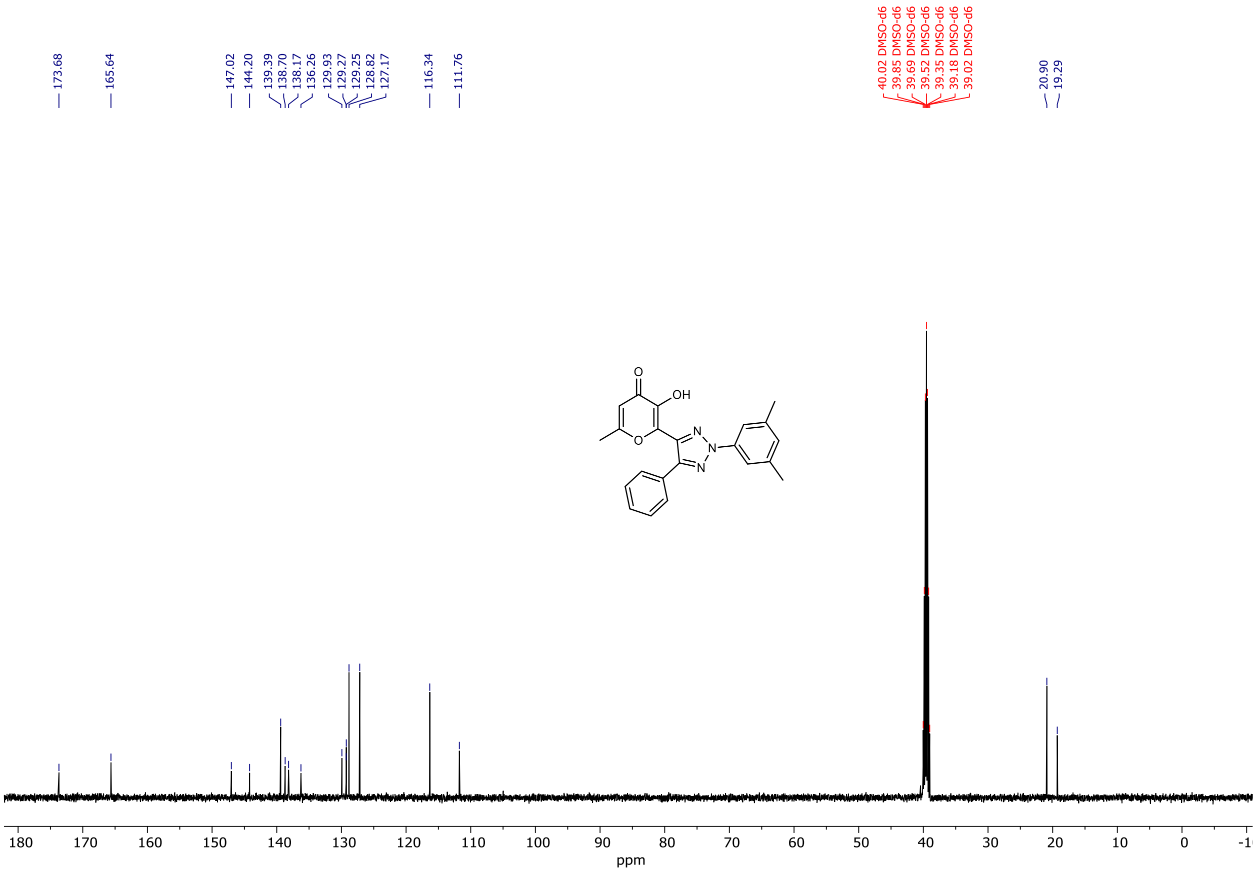

^{1}H NMR spectrum (300 MHz) of **4i** in DMSO-d_6

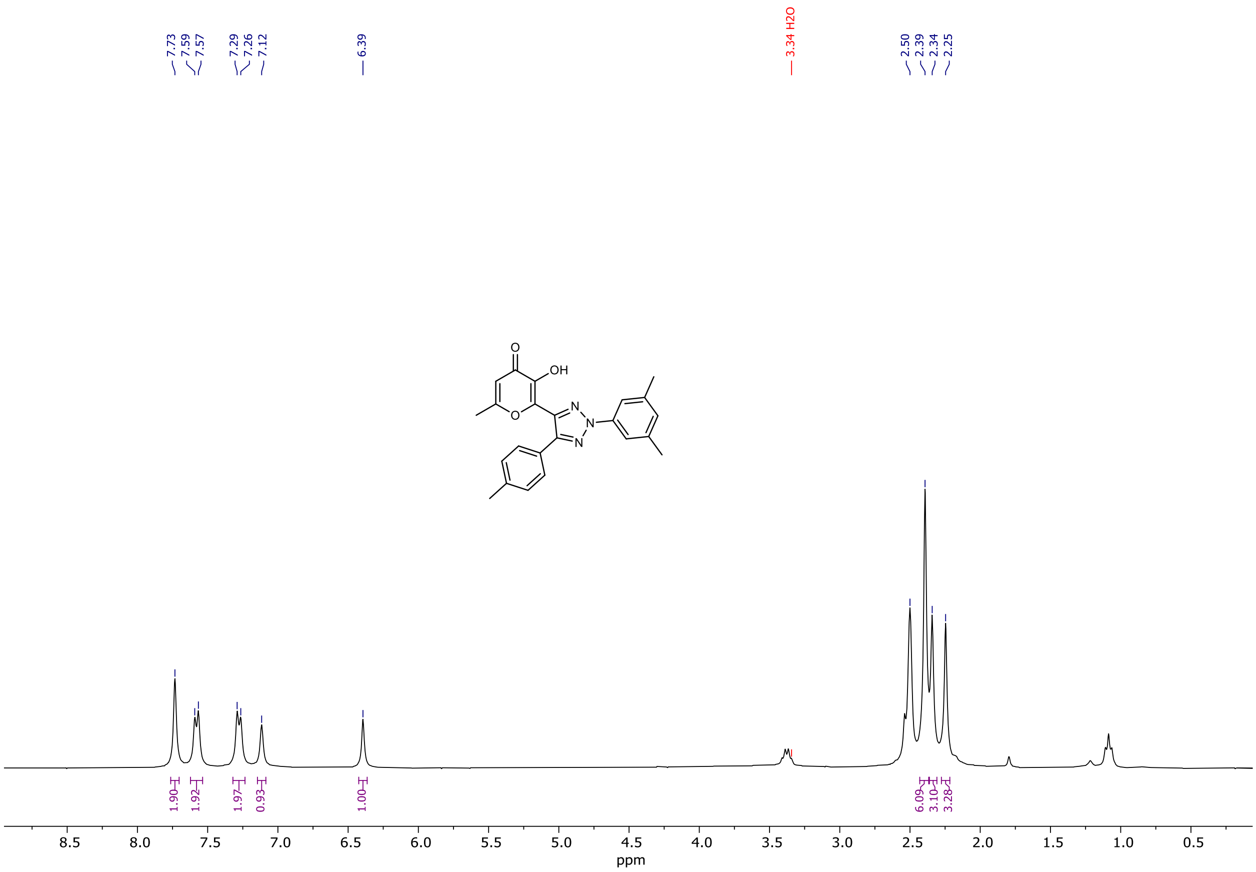

^{13}C {^{1}H} NMR spectrum (126 MHz) of **4i** in DMSO-d_6

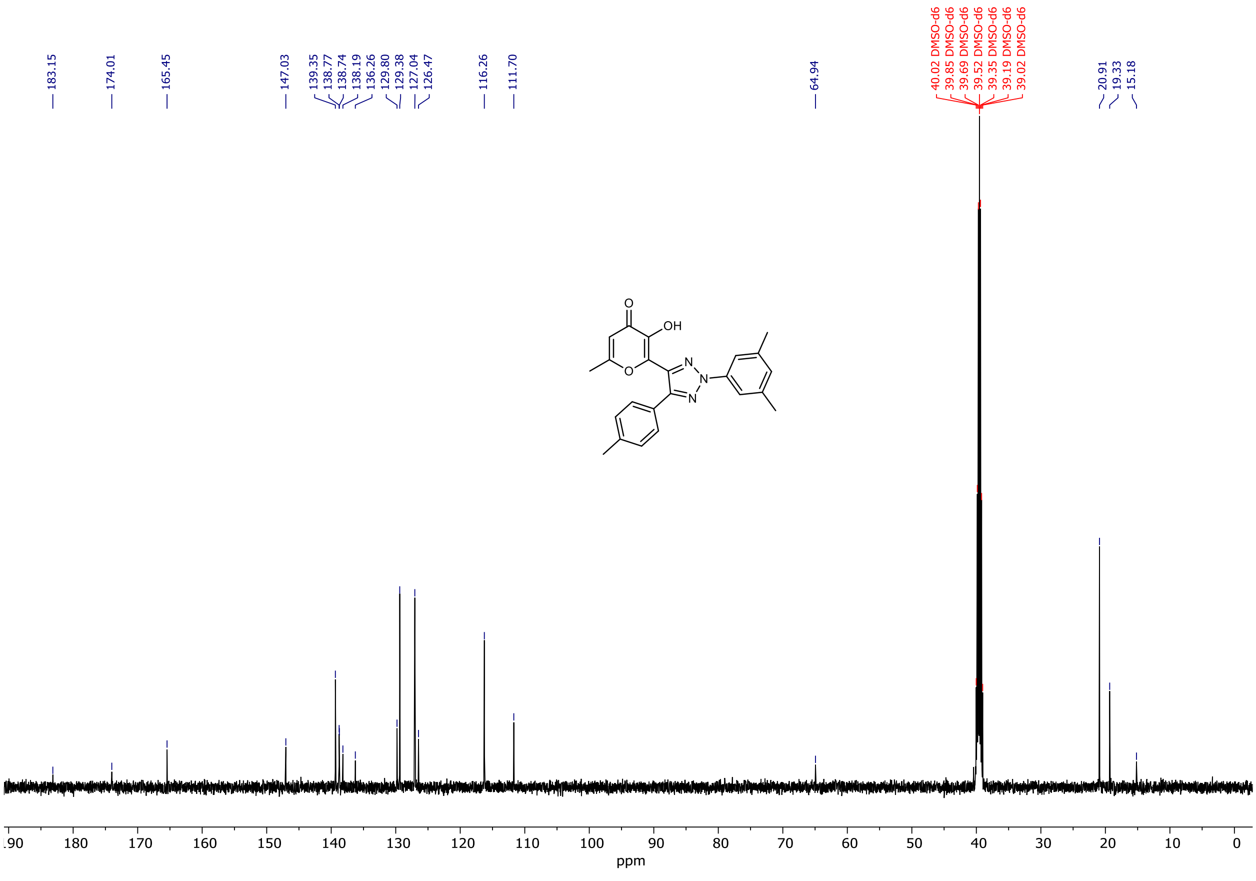

^1H NMR spectrum (300 MHz) of **4j** in DMSO-d_6

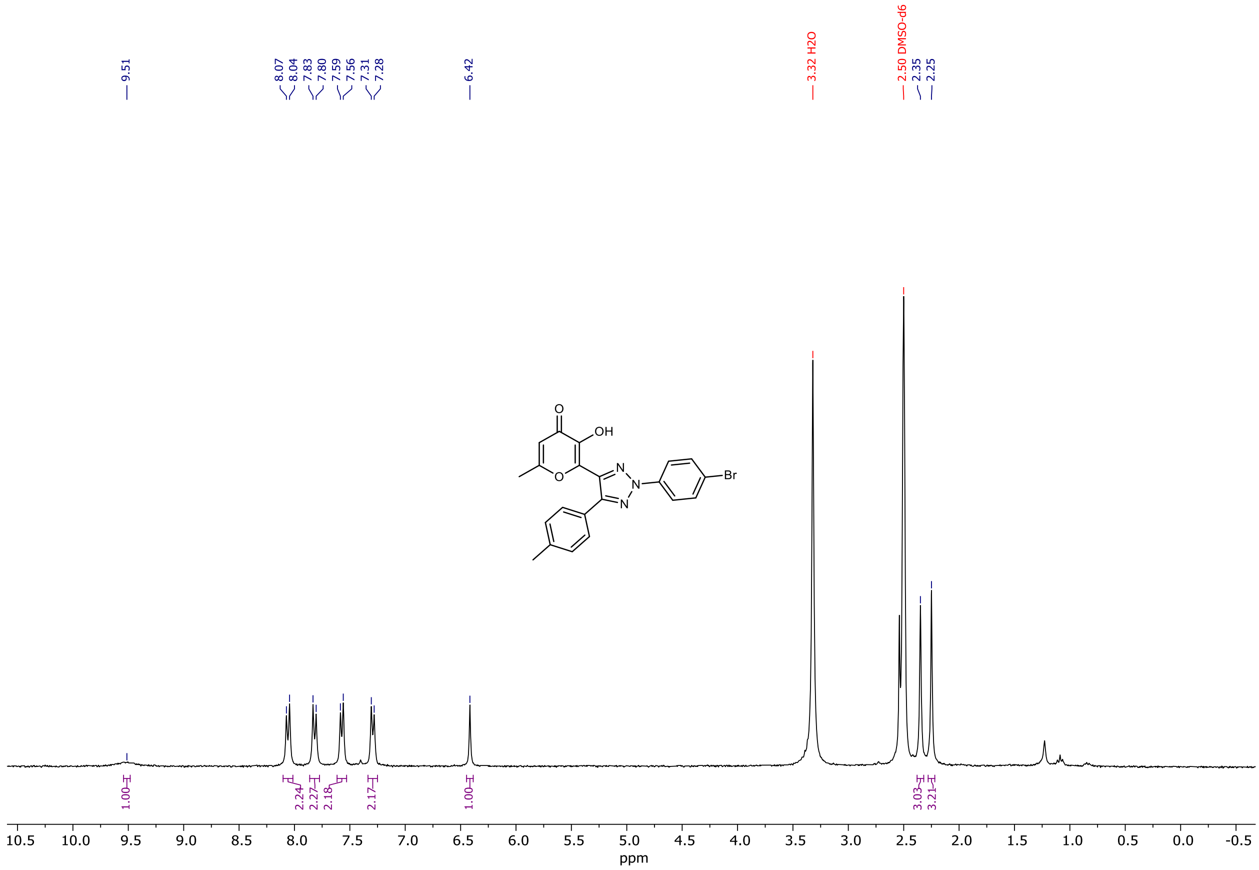

^{13}C {^1H} NMR spectrum (126 MHz) of **4j** in DMSO-d_6

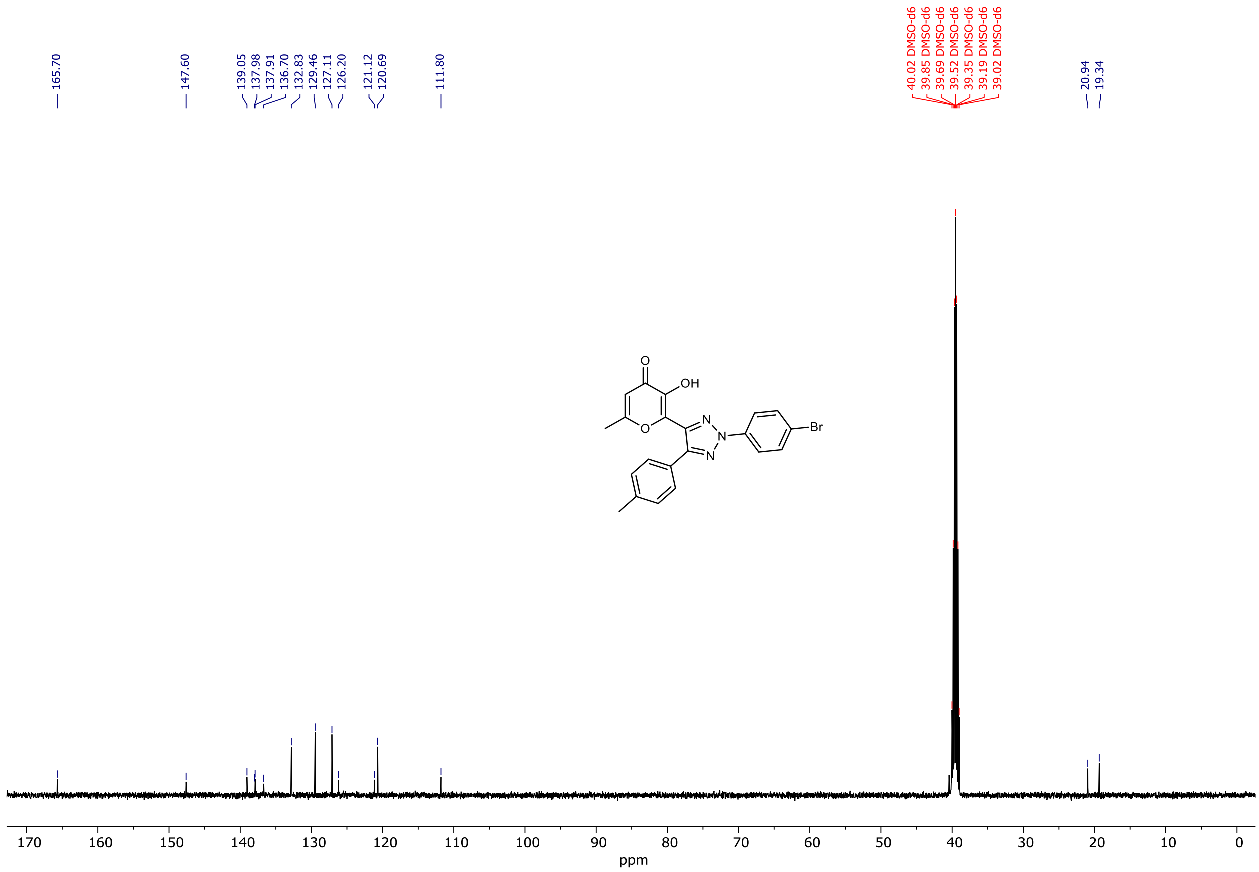

^{1}H NMR spectrum (300 MHz) of **4k** in DMSO-d_6

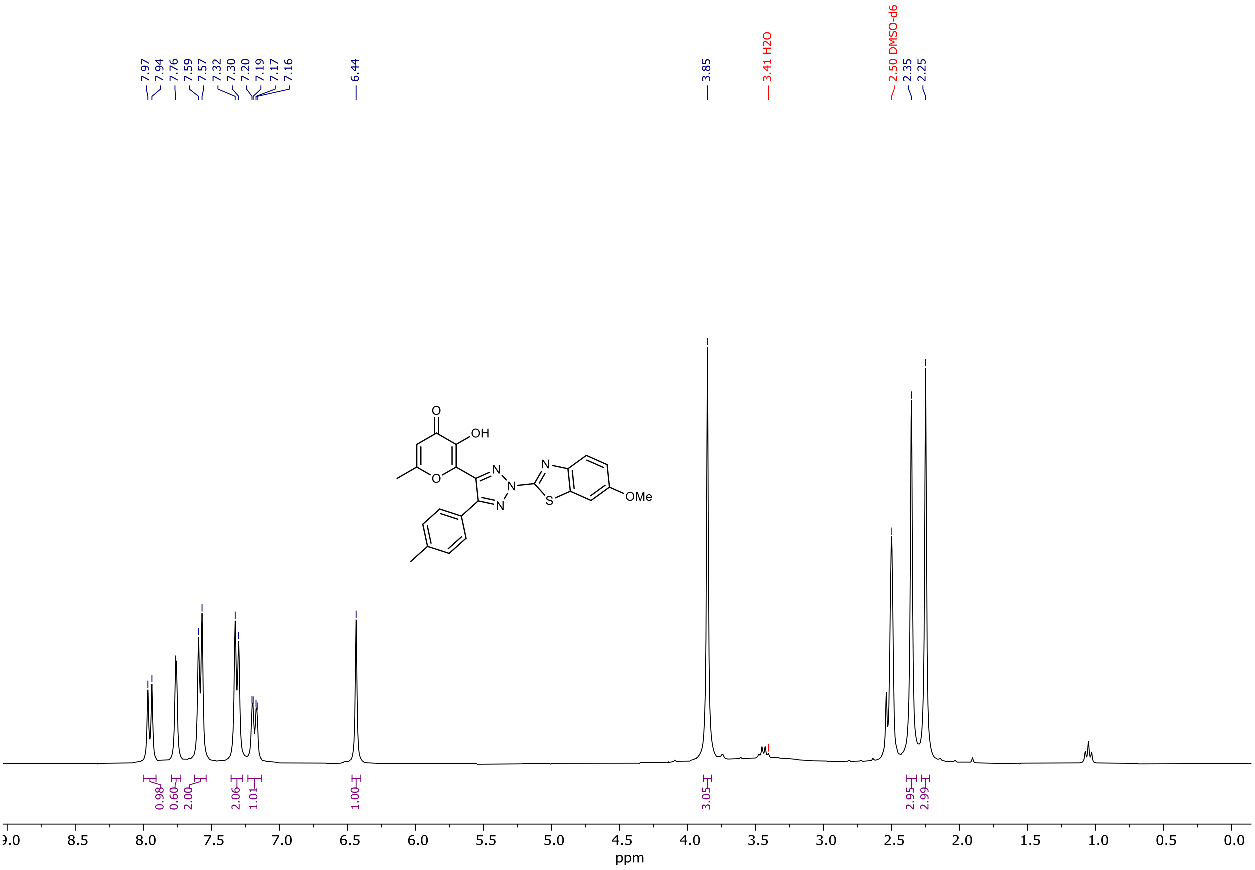

^{13}C {^{1}H} NMR spectrum (126 MHz) of **4k** in DMSO-d_6

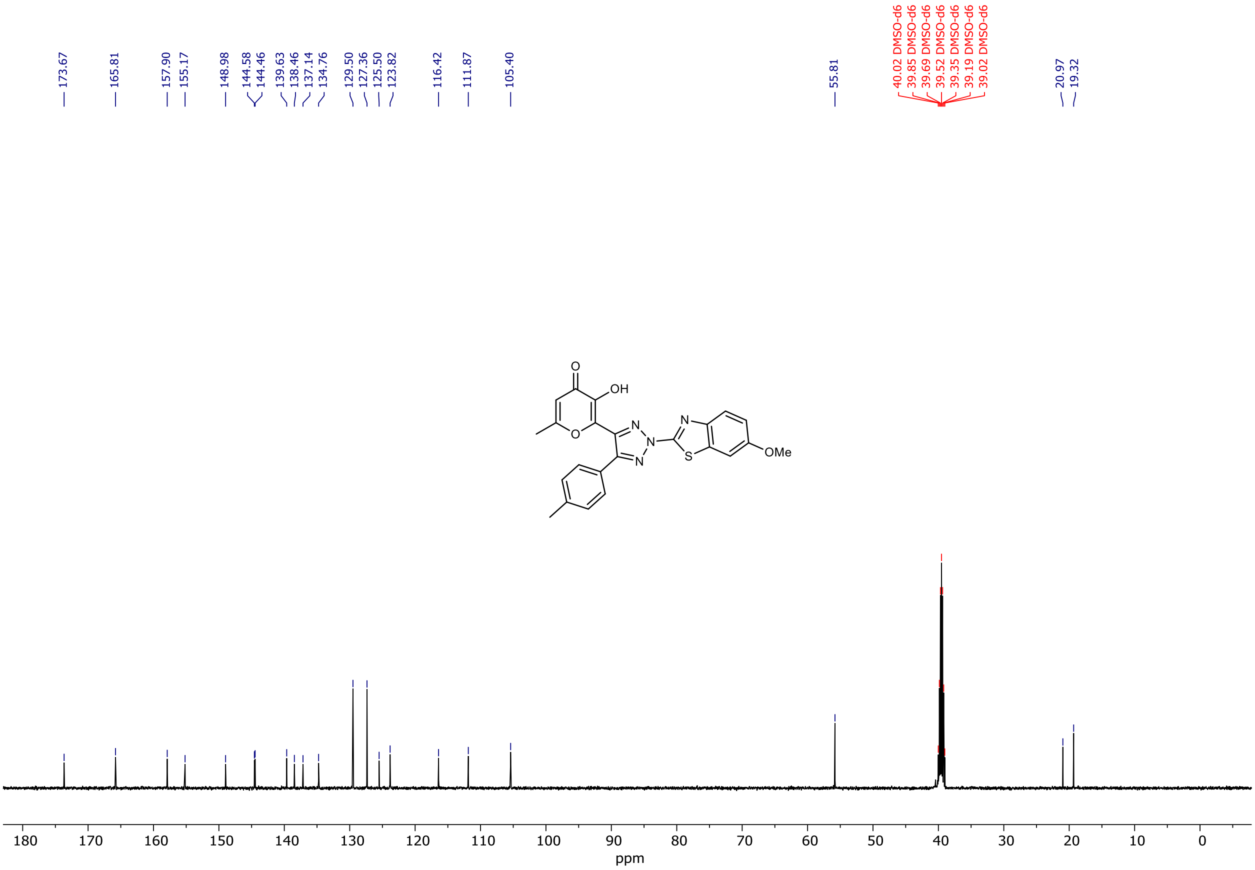

^{1}H NMR spectrum (300 MHz) of **4l** in DMSO-d_6

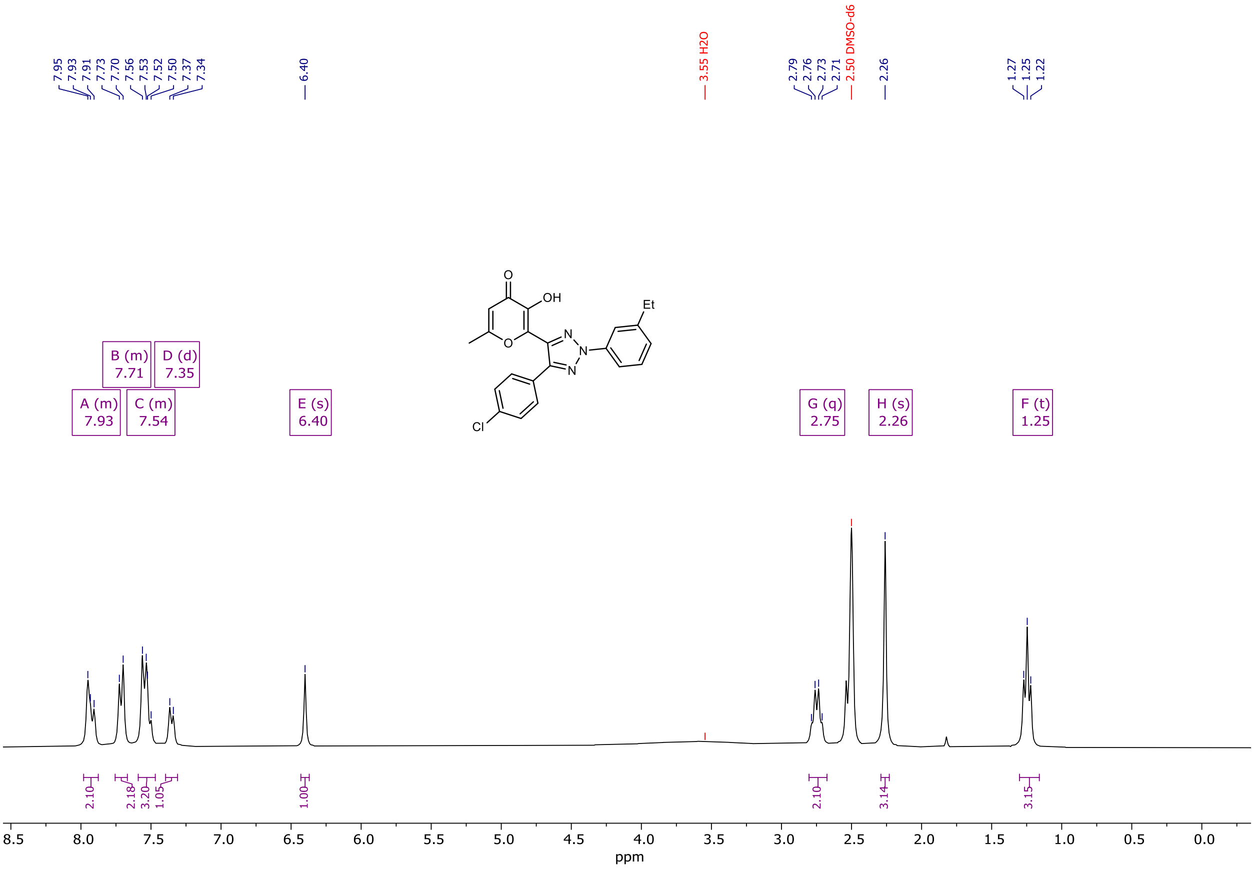

^{13}C {^{1}H} NMR spectrum (126 MHz) of **4l** in DMSO-d_6

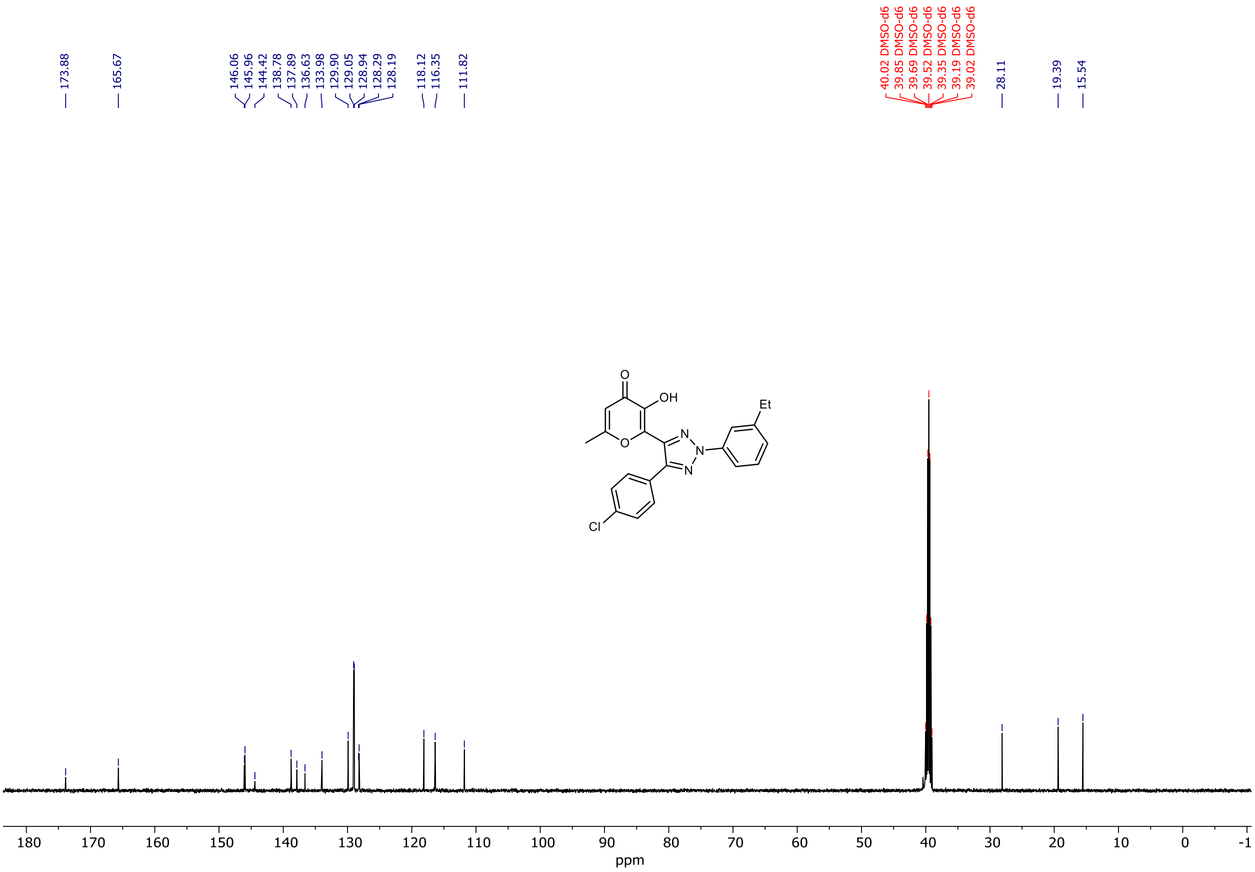

^{1}H NMR spectrum (300 MHz) of **4m** in DMSO-d_6

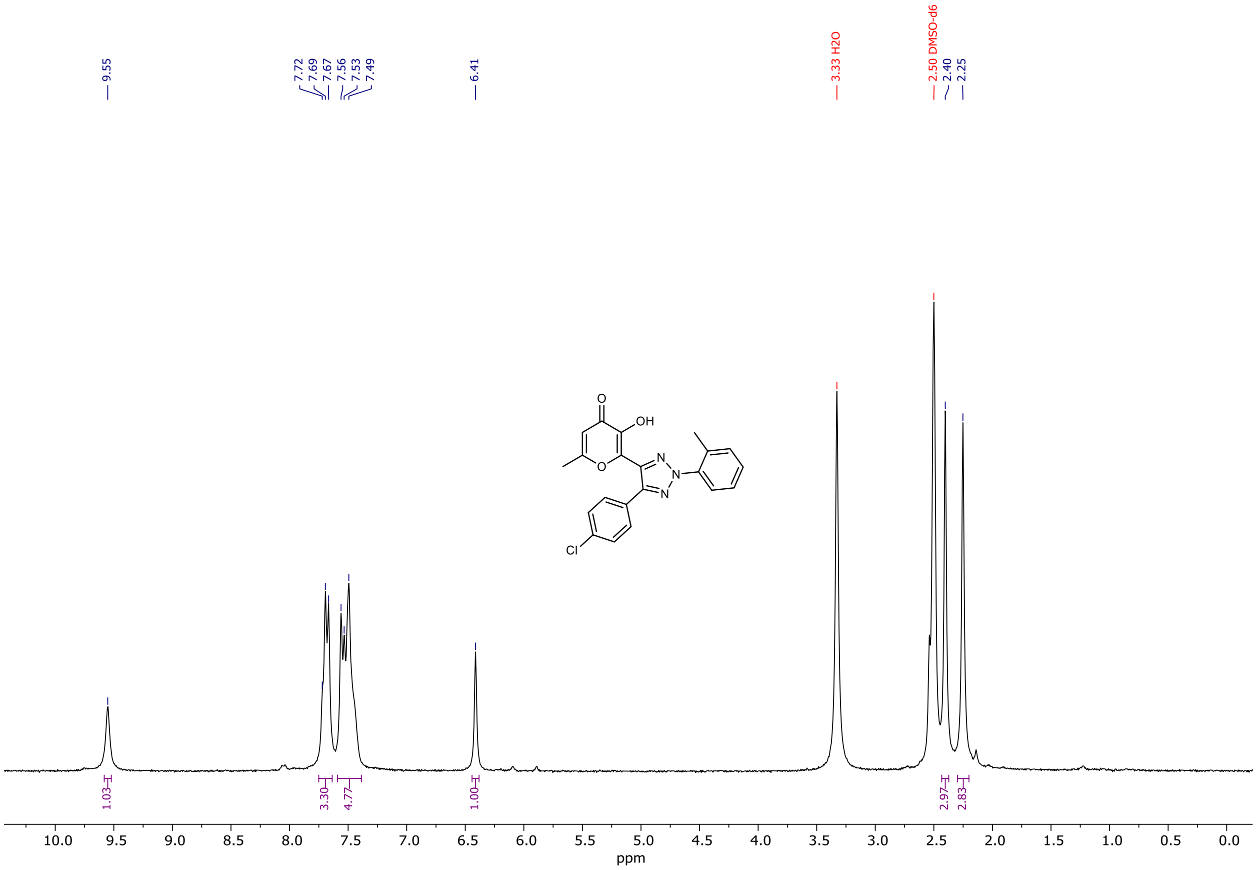

^{13}C {^{1}H} NMR spectrum (126 MHz) of **4m** in DMSO-d_6

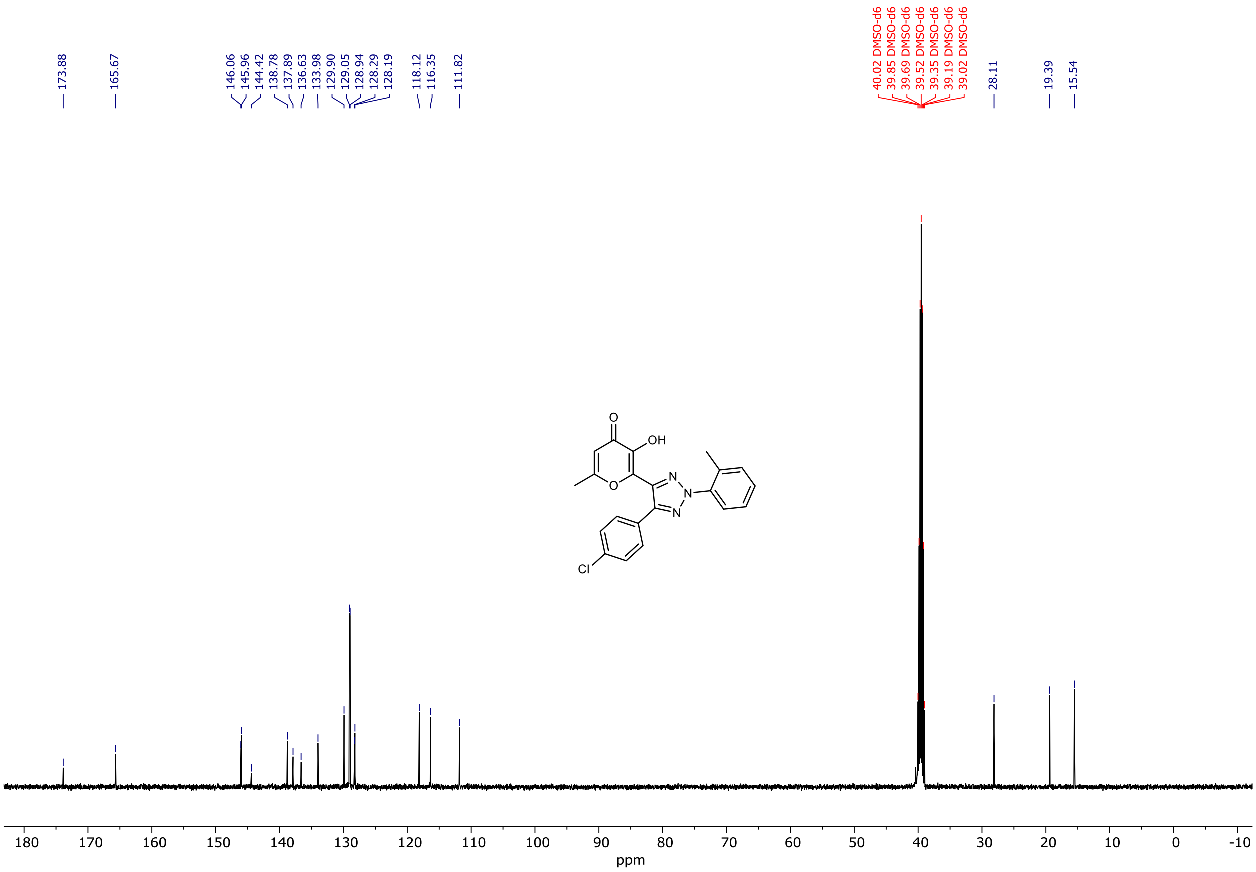

8. NMR ^{1}H and ^{13}C spectra for compounds 6

^{1}H NMR spectrum (300 MHz) of **6a** in DMSO-d_6

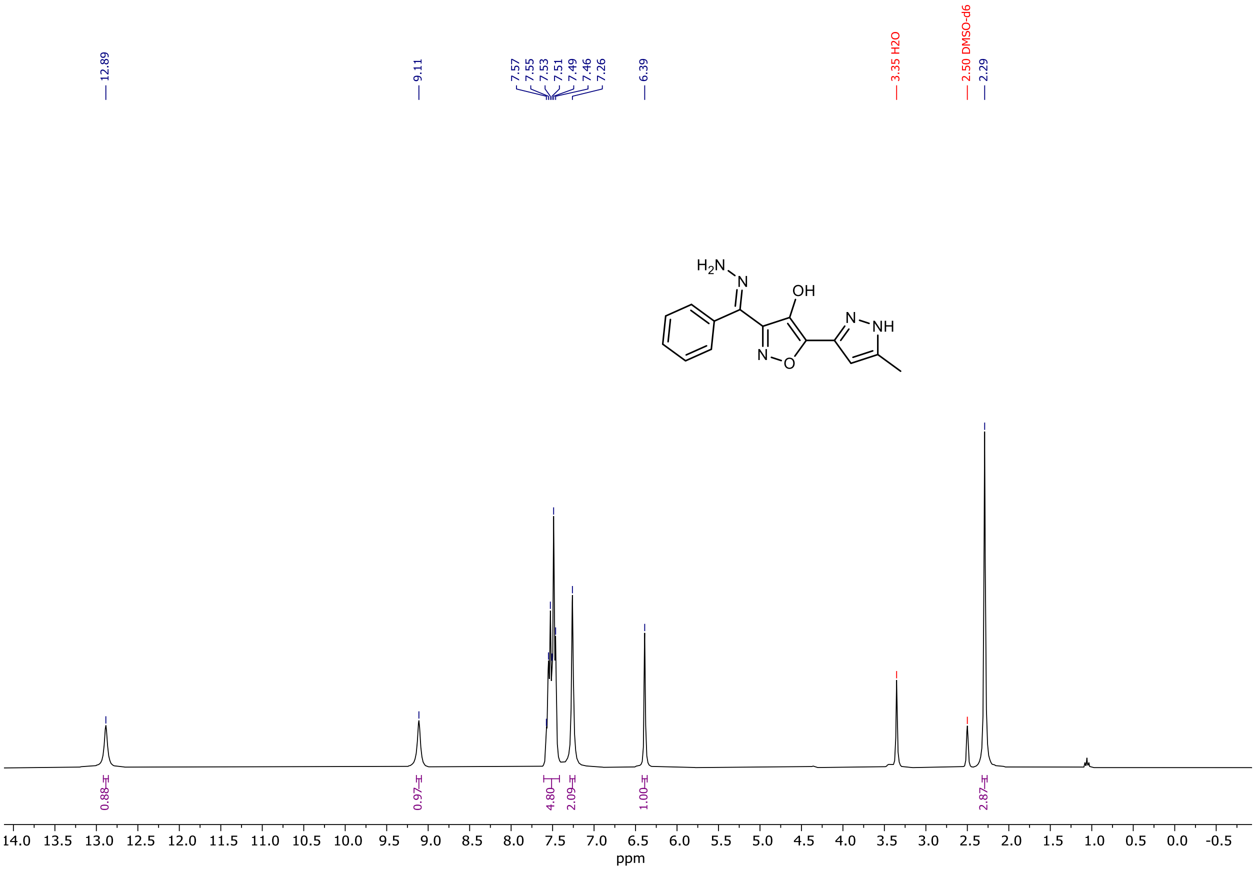

^{13}C {^{1}H} NMR spectrum (126 MHz) of **6a** in DMSO-d_6

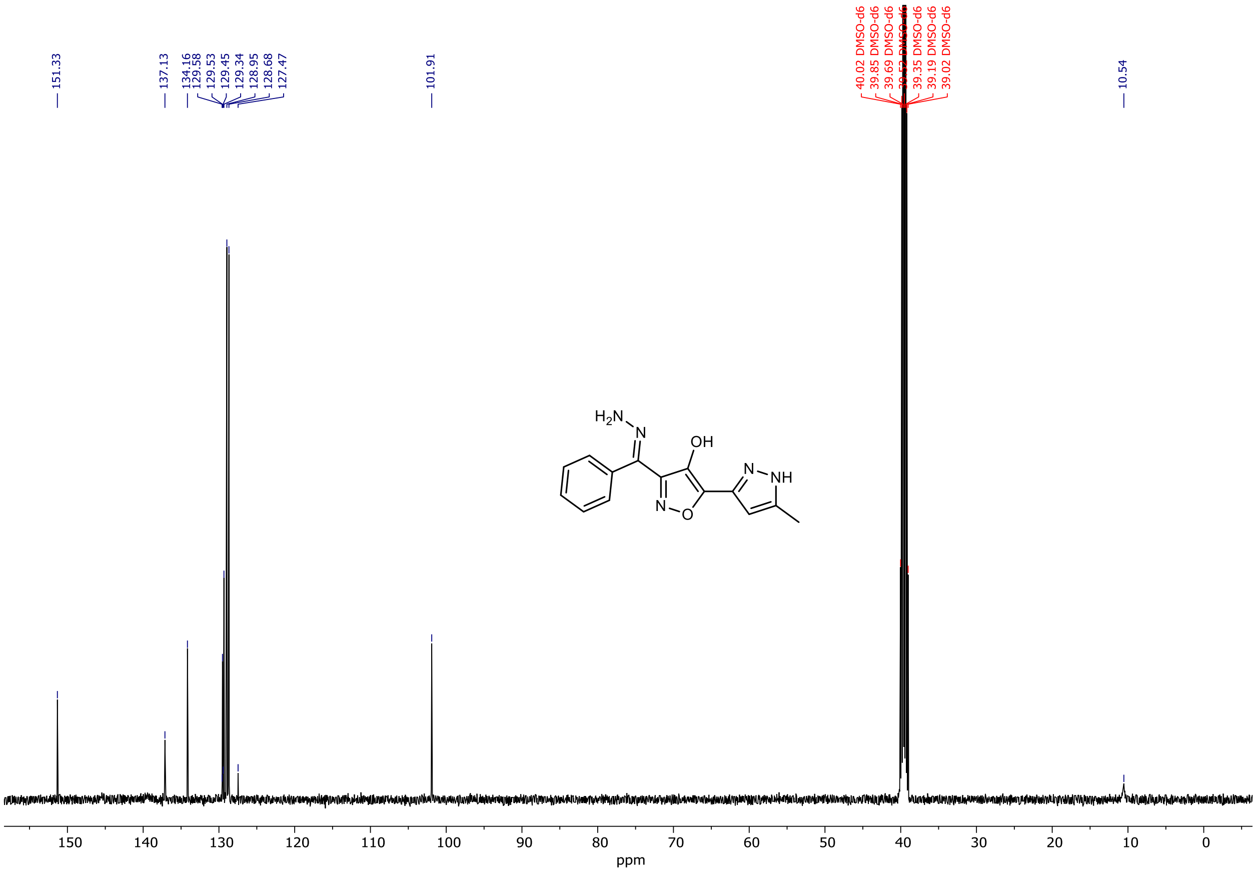

^1H NMR spectrum (300 MHz) of **6b** in DMSO-d_6

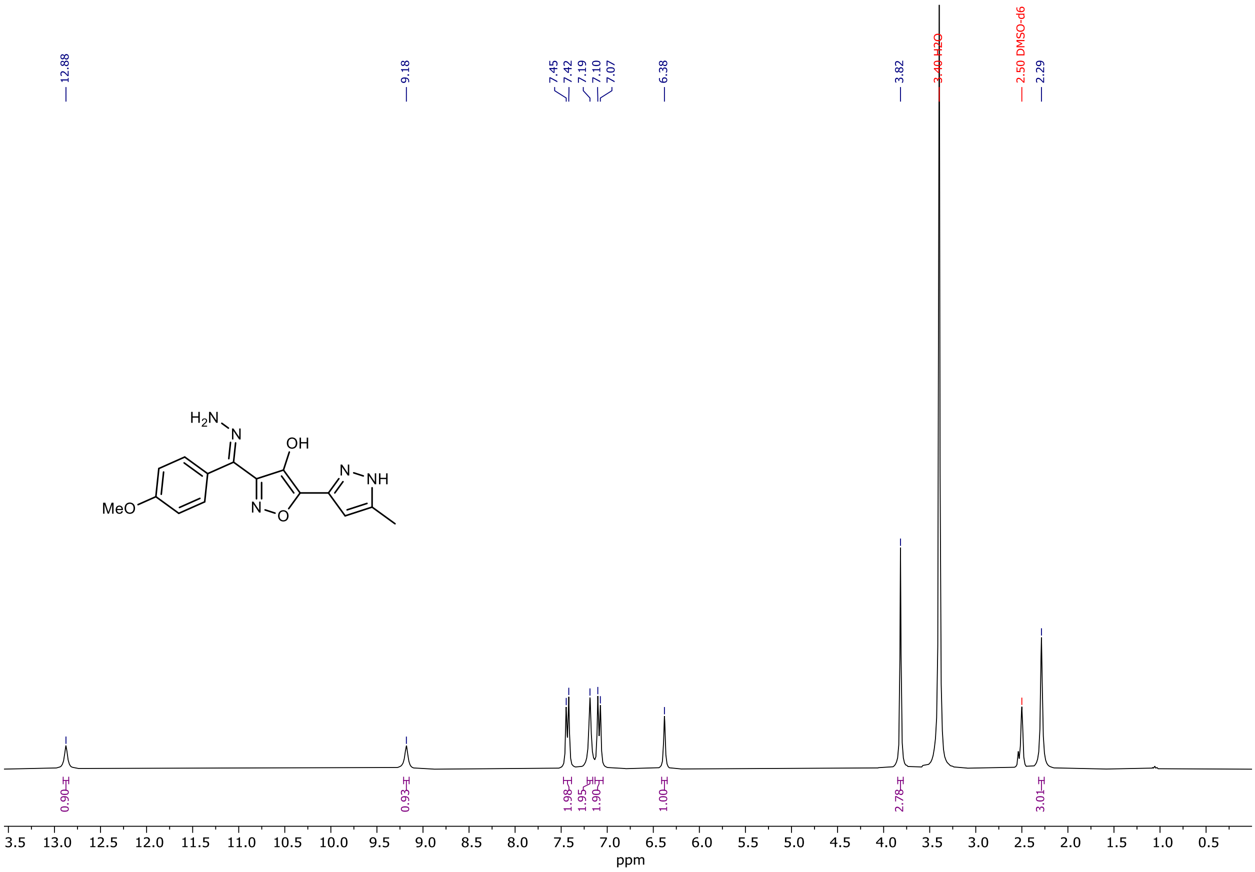

^{13}C {^1H} NMR spectrum (126 MHz) of **6b** in DMSO-d_6

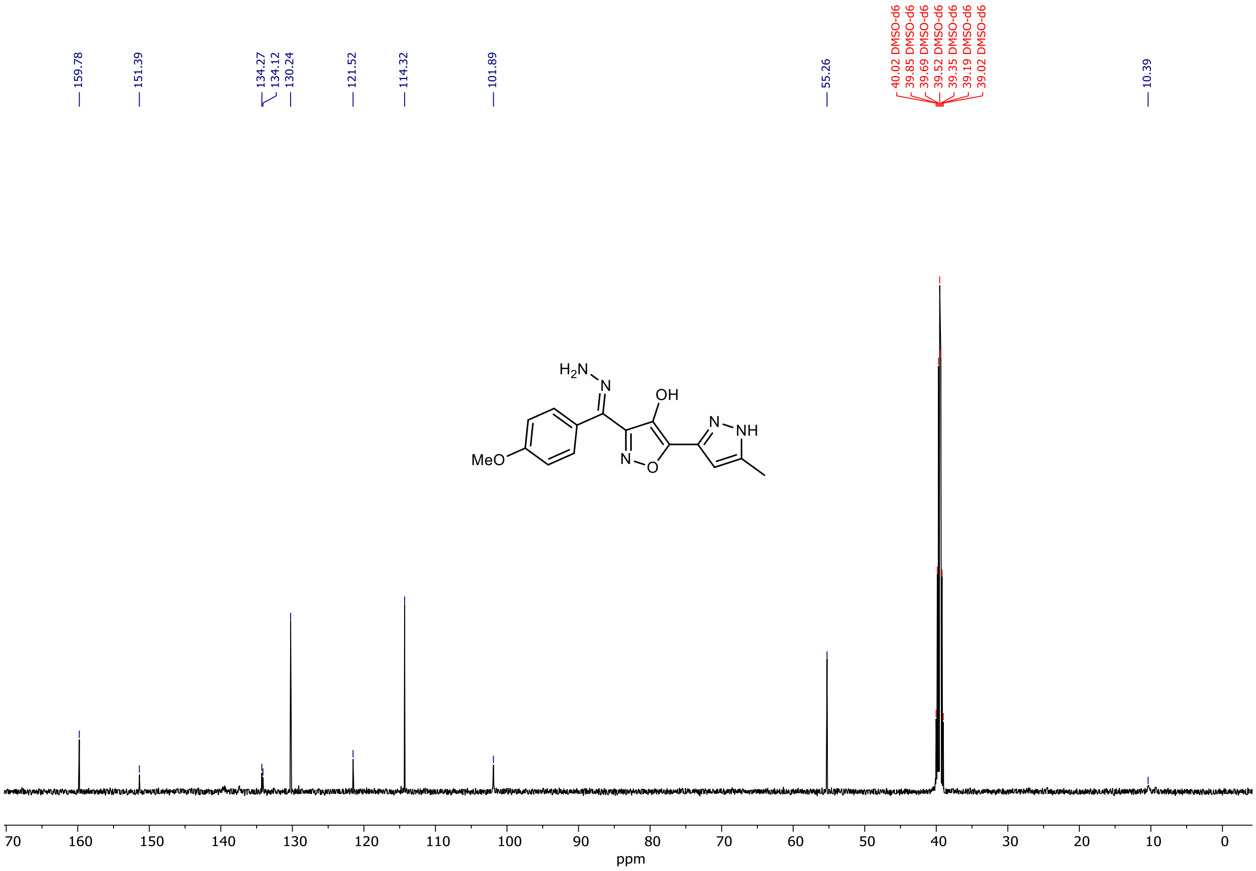

^{1}H NMR spectrum (300 MHz) of **6c** in DMSO-d_6

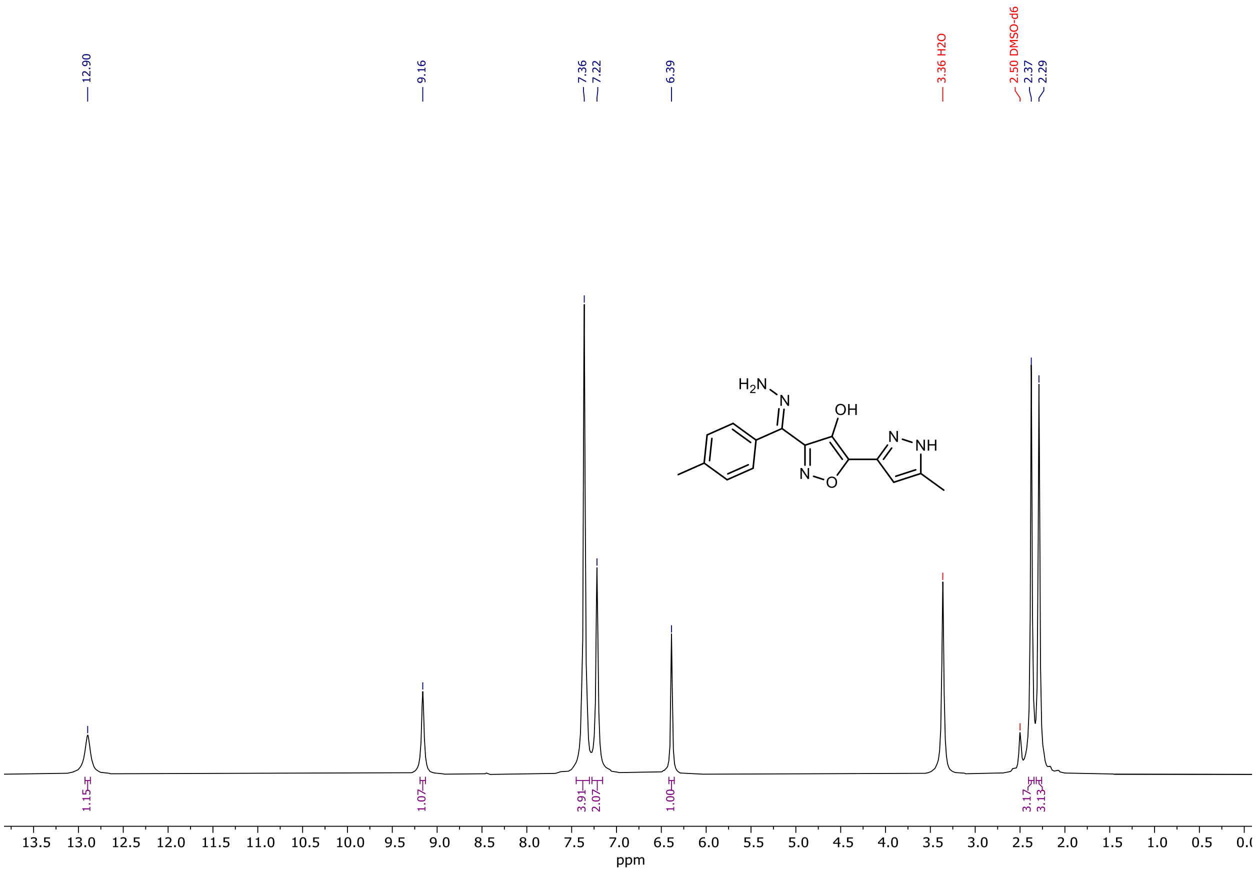

^{13}C {^{1}H} NMR spectrum (126 MHz) of **6c** in DMSO-d_6

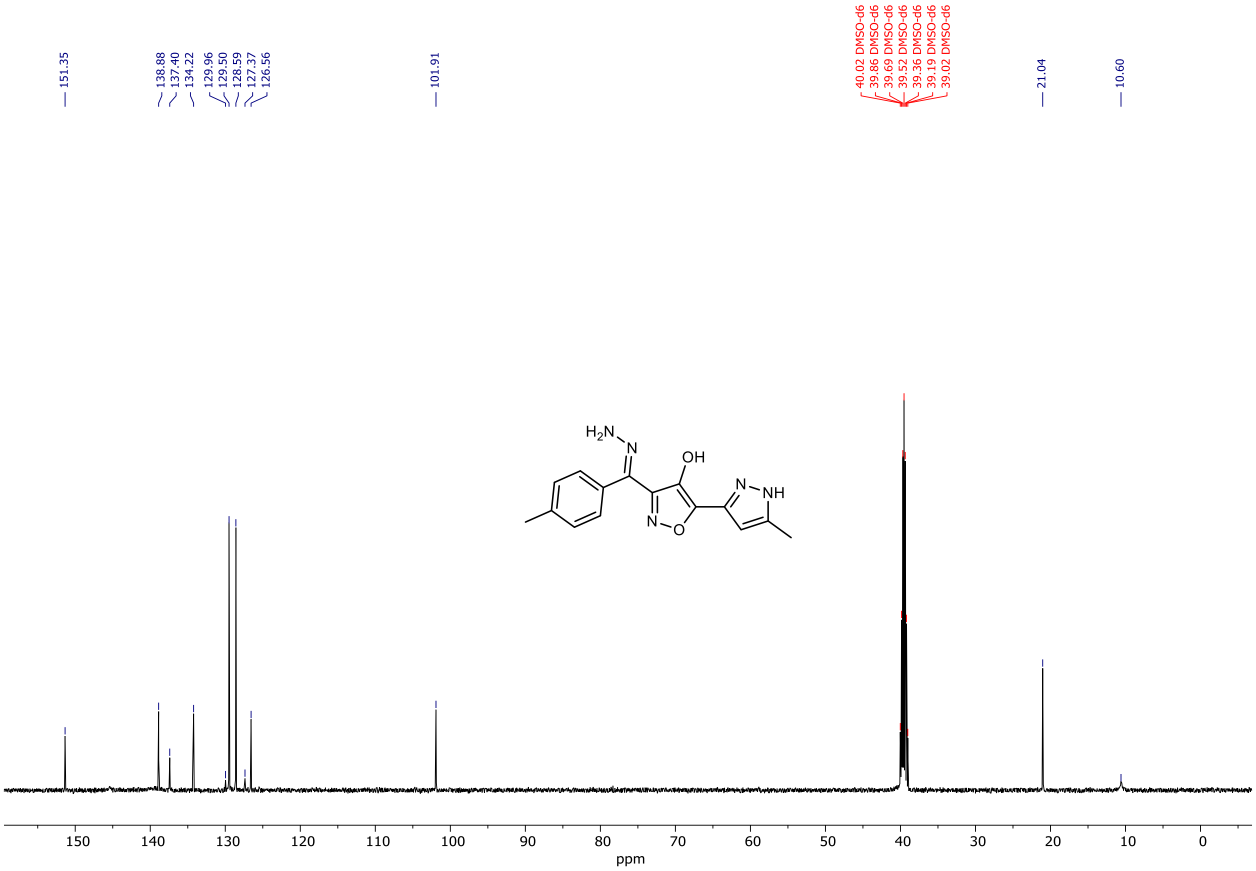

^{1}H NMR spectrum (300 MHz) of **6d** in DMSO-d_6

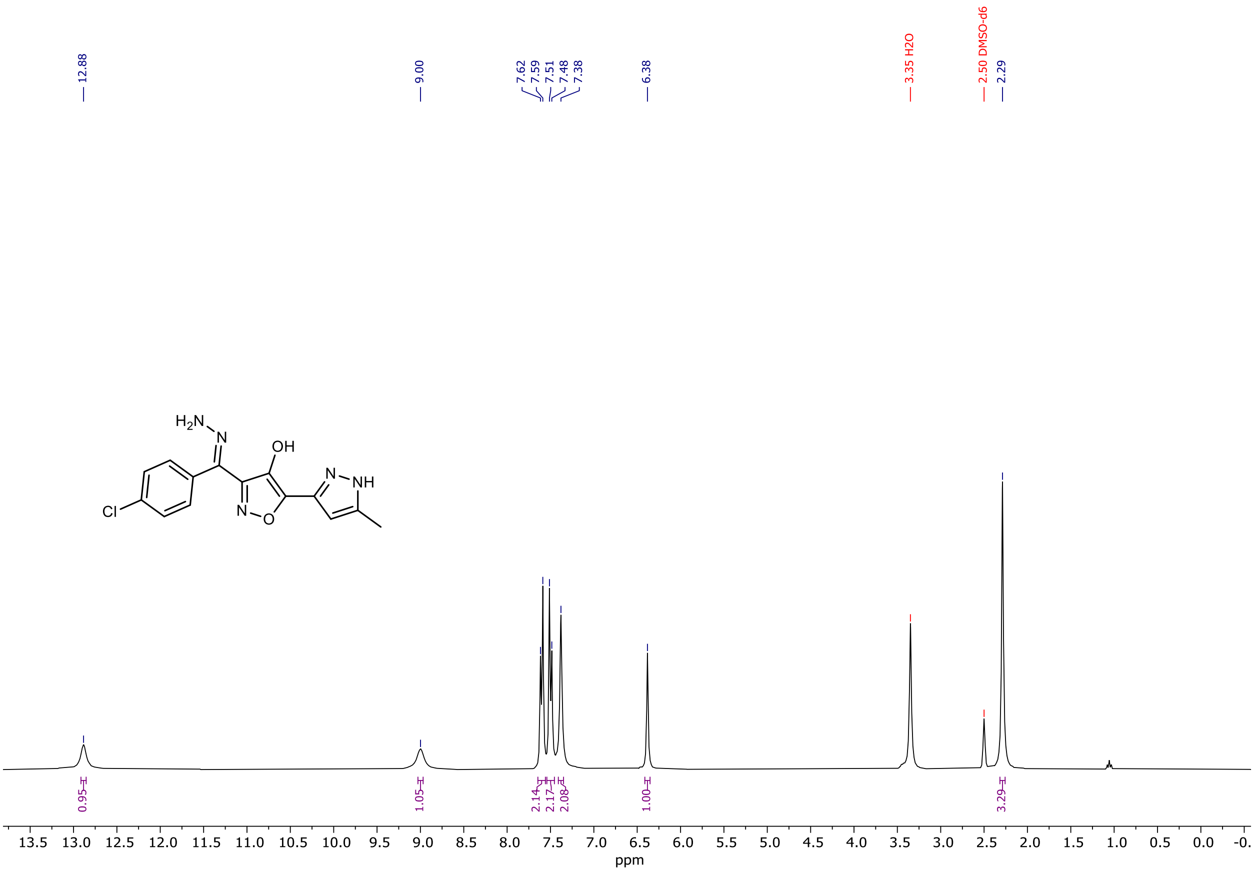

^{13}C {^{1}H} NMR spectrum (126 MHz) of **6d** in DMSO-d_6

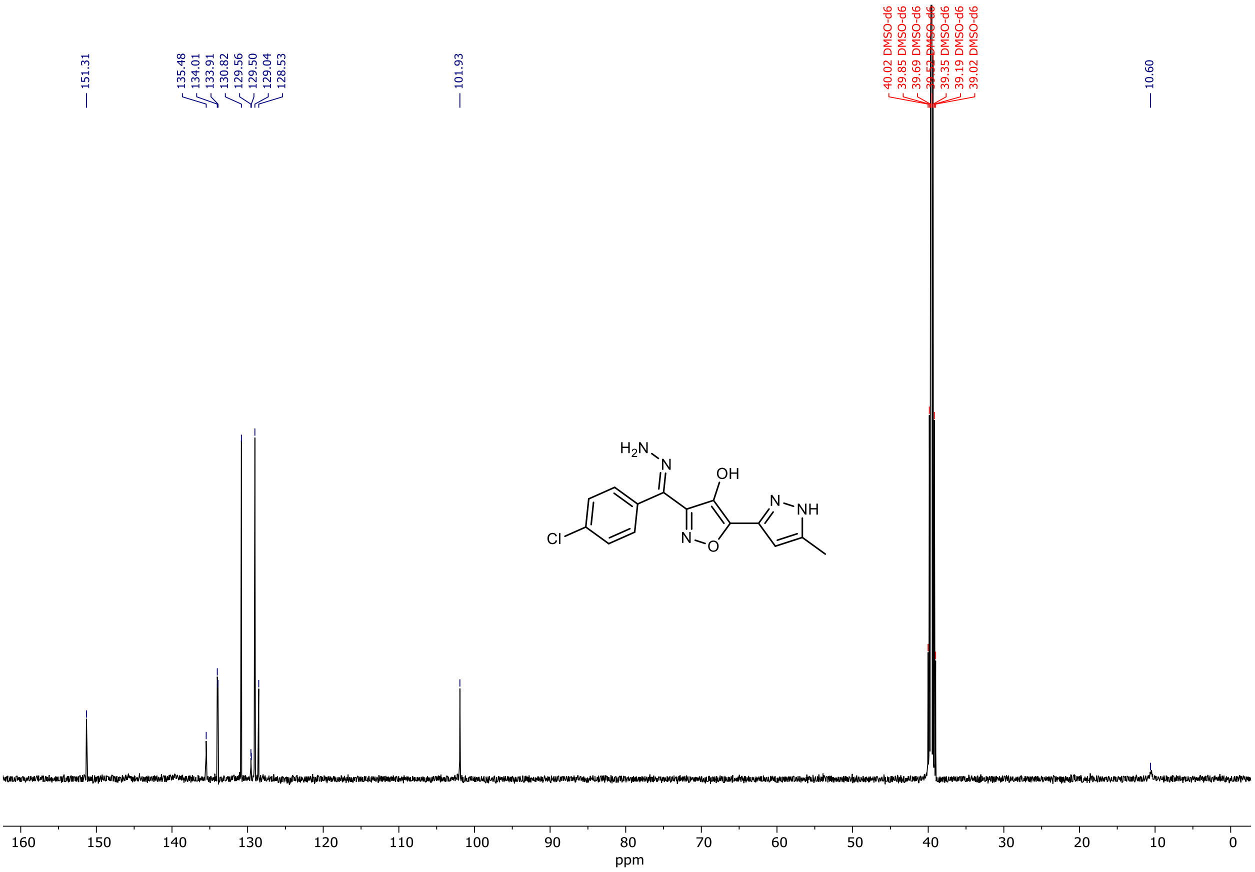

^{1}H NMR spectrum (300 MHz) of **6e** in DMSO-d_6

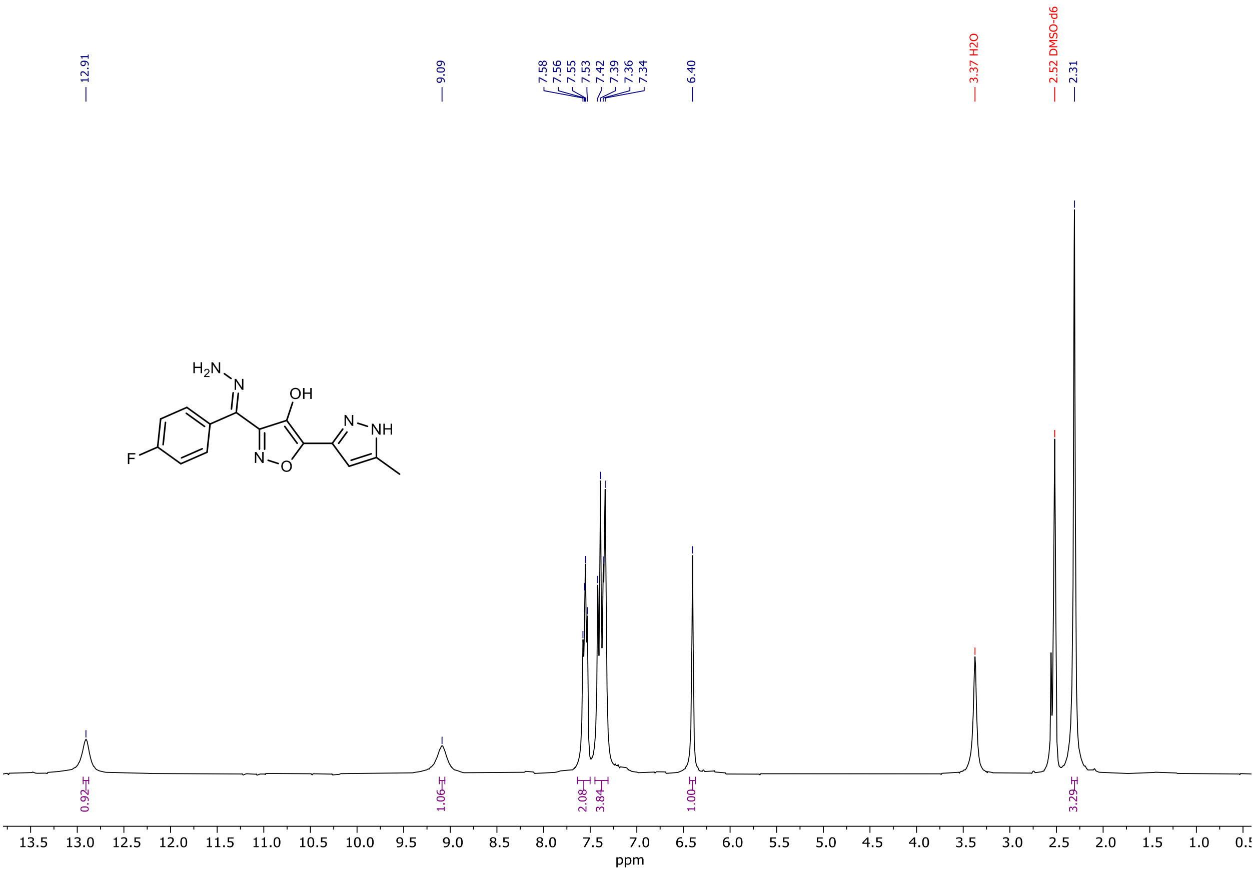

^{13}C {^{1}H} NMR spectrum (126 MHz) of **6e** in DMSO-d_6

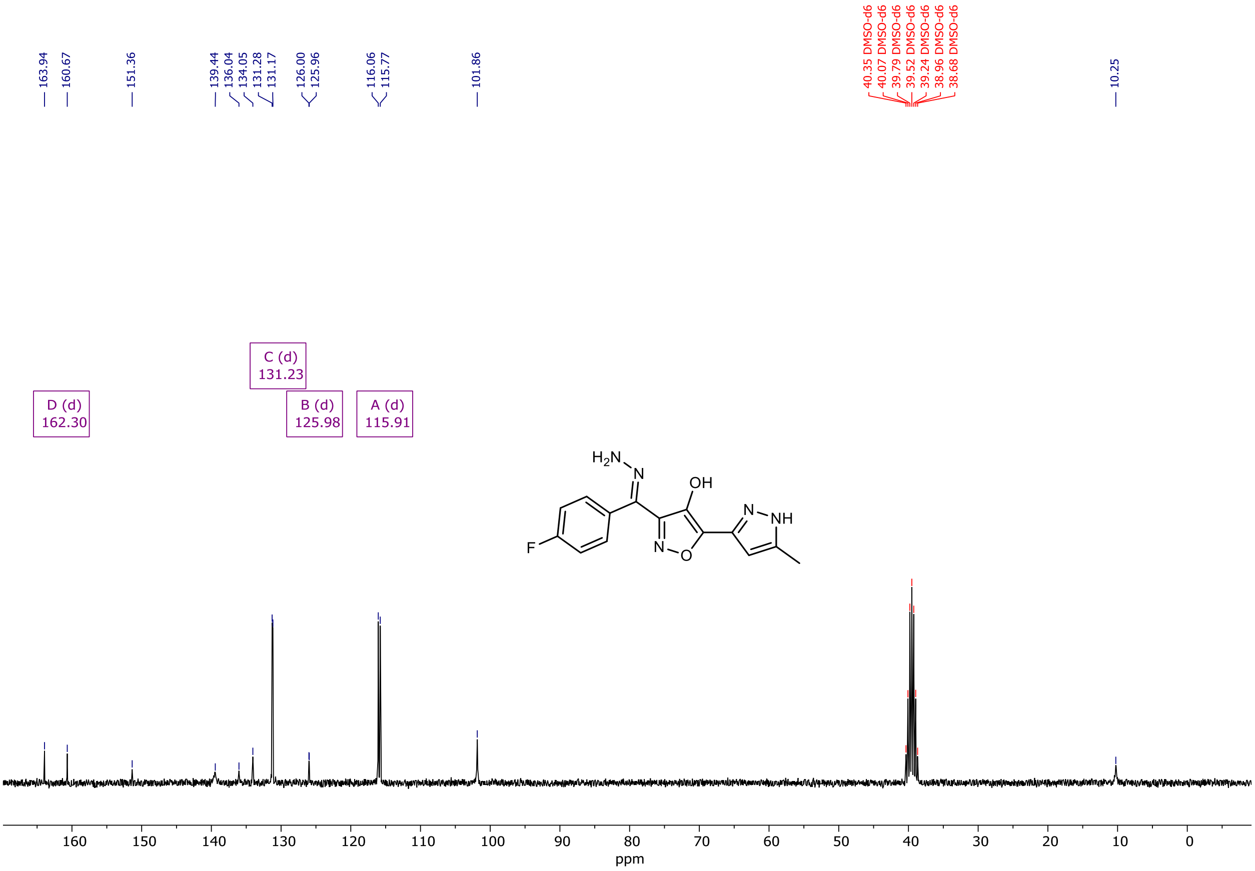

^{1}H NMR spectrum (300 MHz) of **6f** in DMSO-d_6

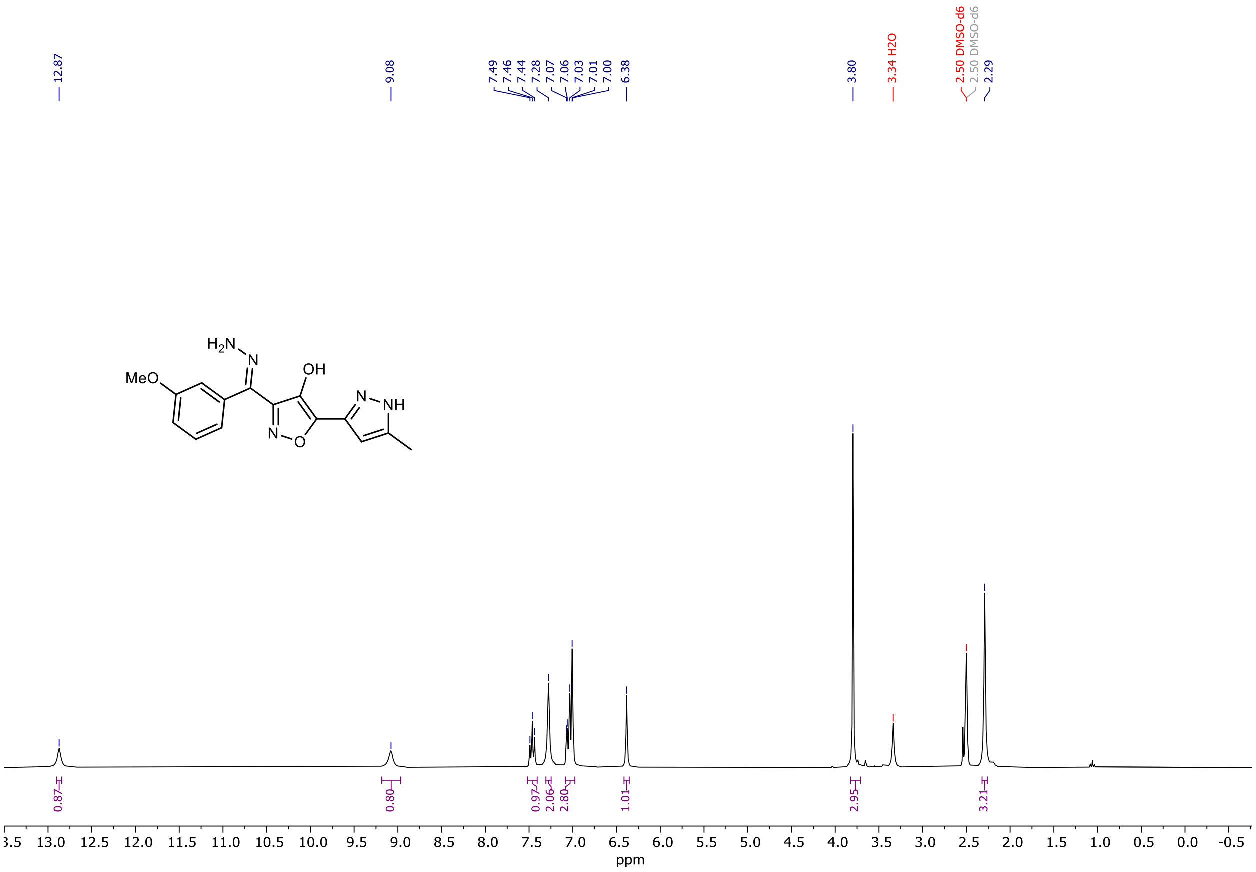

^{13}C {^{1}H} NMR spectrum (126 MHz) of **6f** in DMSO-d_6

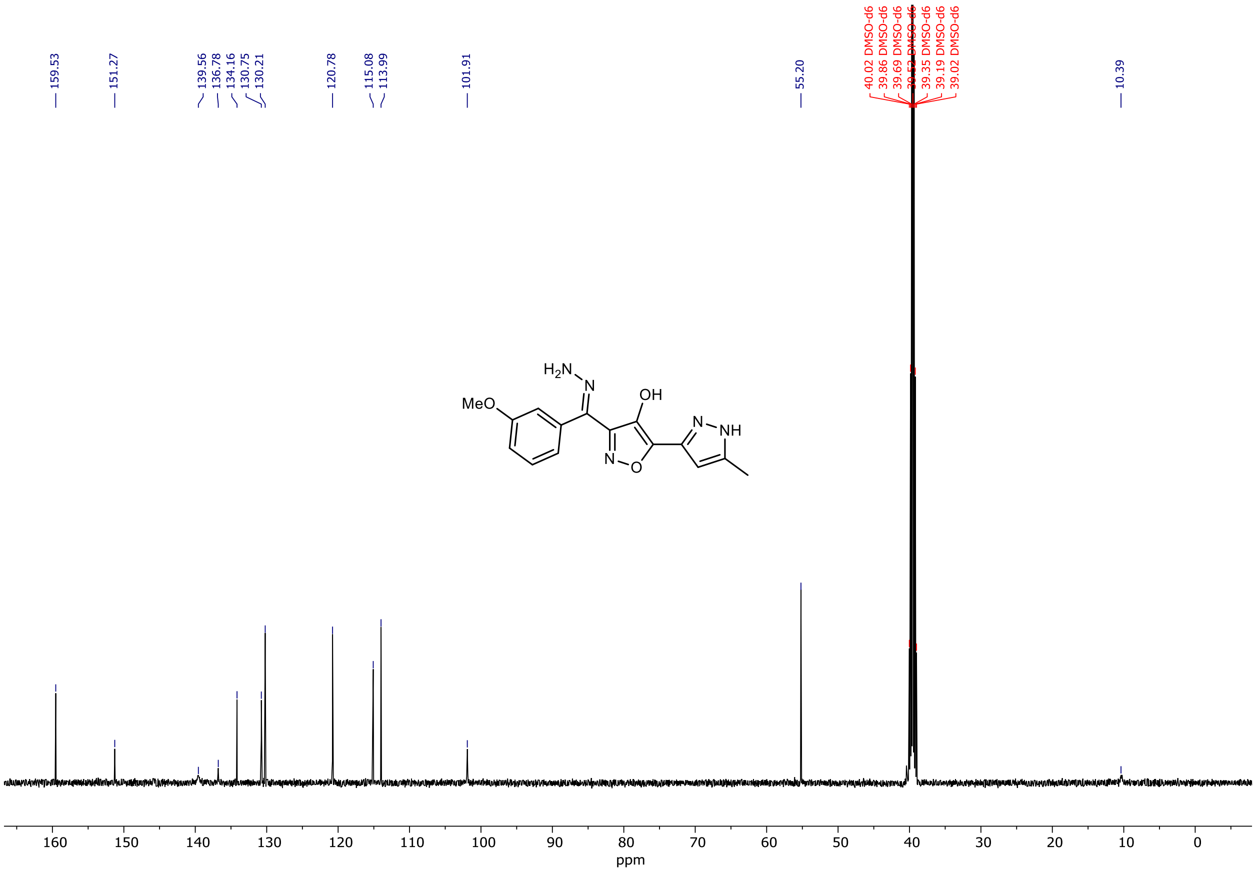

9. NMR ^{1}H and ^{13}C spectra for compound 7

^{1}H NMR spectrum (300 MHz) of **7** in DMSO-d_6

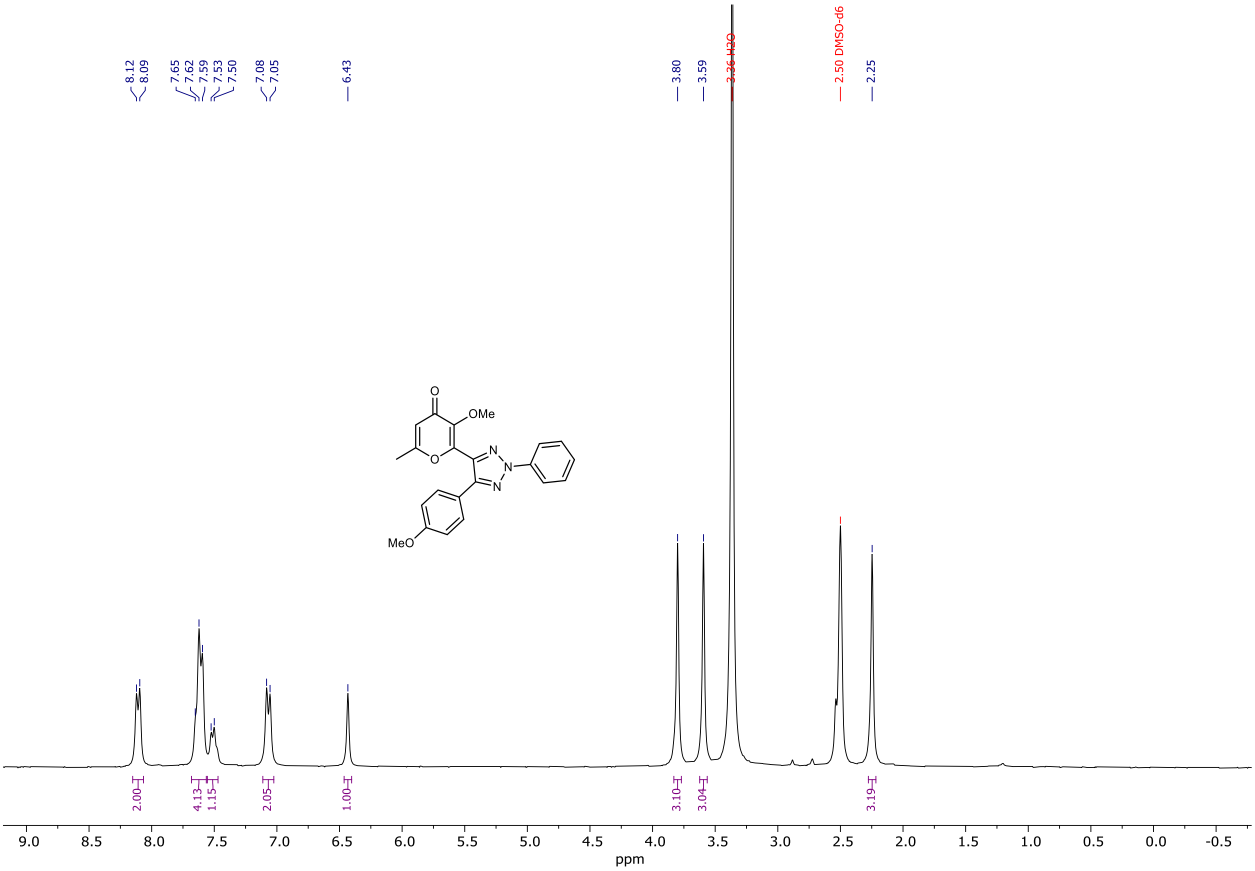

$^{13}C\{^{1}H\}$ NMR spectrum (126 MHz) of **7** in DMSO-d_6

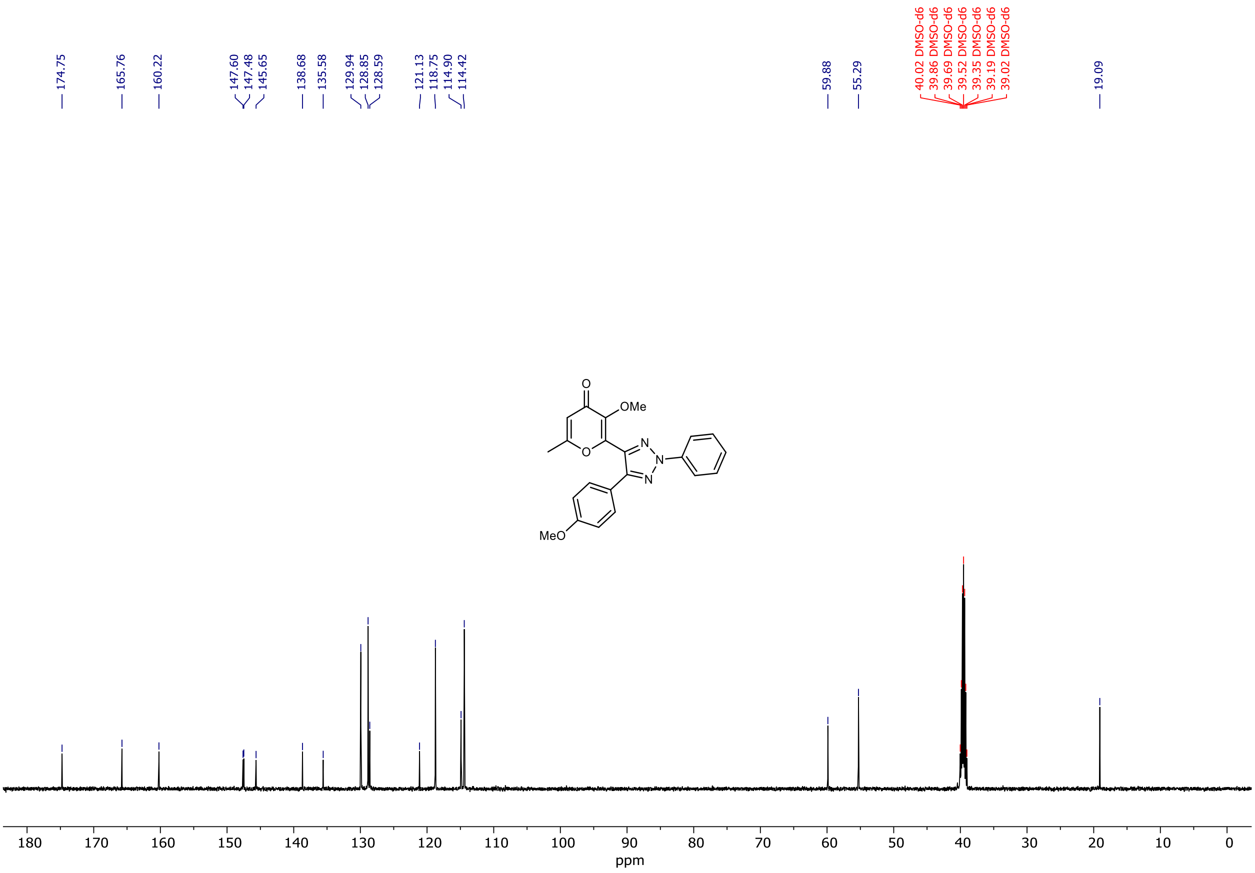

10. X-ray crystallographic data and refinement details

X-ray diffraction data were collected at 100K on a four-circle Rigaku Synergy S diffractometer equipped with a HyPix6000HE area-detector (kappa geometry, shutterless ω-scan technique), using graphite monochromatized Cu K[illegible]-radiation. The intensity data were integrated and corrected for absorption and decay by the CrysAlisPro program[1]. The structure was solved by direct methods using SHELXT[2] and refined on F^2 using SHELXL-2018[3] in the OLEX2 program.[4] All non-hydrogen atoms were refined with individual anisotropic displacement parameters. The location of hydrogen atom H3A was found from the electron density-difference map; this hydrogen atom was refined with an individual isotropic displacement parameter. All other hydrogen atoms were placed in ideal calculated positions and refined as riding atoms with relative isotropic displacement parameters. The Mercury program suite[5] was used for molecular graphics.

1. CrysAlisPro. Version 1.171.41.106a. *Rigaku Oxford Diffraction*, **2021**.

2. Sheldrick, G. M. SHELXT - Integrated space-group and crystal-structure determination. *Acta Cryst.* **2015**, A71(1), 3-8. http://doi.org/10.1107/S2053273314026370

3. Sheldrick, G. M. Crystal structure refinement with SHELXL. *Acta Cryst.* **2015**, C71(1), 3-8. http://doi.org/10.1107/S2053229614024218

4. Dolomanov O.V.; Bourhis L.J.; Gildea R.J.; Howard J.A.K.; Puschmann H. OLEX2: a complete structure solution, refinement and analysis program. *J. Appl. Cryst.* **2009**, 42(2), 339-341. http://doi.org/10.1107/S0021889808042726

5. Macrae, C. F.; Sovago, I.; Cottrell, S. J.; Galek, P. T. A.; McCabe, P.; Pidcock, E.; Platings, M.; Shields, G. P.; Stevens, J. S.; Towler, M.; Wood, P. A. Mercury 4.0: from visualization to analysis, design and prediction. *J. Appl. Cryst.* **2020**, 53, 226-235. https://doi.org/10.1107/S1600576719014092

Crystallographic data for *2-(2-(2,4-difluorophenyl)-5-phenyl-2H-1,2,3-triazol-4-yl)-3-hydroxy-6-methyl-4H-pyran-4-one* **4g**

Table 1. Crystal data and structure refinement for **4g**.

Identification code	**2343878**
Empirical formula	$C_{20}H_{13}F_2N_3O_3$
Formula weight	381.33
Temperature	100.00(16) K
Wavelength	1.54184 Å
Crystal system	Triclinic
Space group	P $\bar{1}$
Unit cell dimensions	a = 9.00930(10) Å α= 110.9280(10)°.
	b = 9.42790(10) Å β= 108.7570(10)°.
	c = 11.2567(2) Å γ = 91.0880(10)°.
Volume	835.88(2) Å^3
Z	2
Density (calculated)	1.515 g/cm^3
Absorption coefficient	1.013 mm^{-1}
F(000)	392
Crystal size	0.67 x 0.36 x 0.29 mm^3
Theta range for data collection	4.492 to 79.856°.
Index ranges	-11<=h<=11, -11<=k<=12, -14<=l<=14
Reflections collected	49406
Independent reflections	3606 [R(int) = 0.0281]
Observed reflections	3576
Completeness to theta = 67.684°	99.9 %
Absorption correction	Gaussian
Max. and min. transmission	1.000 and 0.212
Refinement method	Full-matrix least-squares on F^2
Data / restraints / parameters	3606 / 0 / 259
Goodness-of-fit on F^2	1.040
Final R indices [I>2sigma(I)]	R1 = 0.0361, wR2 = 0.0910
R indices (all data)	R1 = 0.0364, wR2 = 0.0912
Extinction coefficient	0.0201(9)
Largest diff. peak and hole	0.417 and -0.365 e.Å^{-3}

Table 2. Atomic coordinates (x 10^4) and equivalent isotropic displacement parameters ($Å^2$x 10^3) for **4g**. U(eq) is defined as one third of the trace of the orthogonalized U^{ij} tensor.

	x	y	z	U(eq)
F(1)	4559(1)	5679(1)	8972(1)	26(1)
F(2)	1706(1)	5300(1)	4580(1)	45(1)
O(1)	2326(1)	5453(1)	430(1)	16(1)
O(2)	-396(1)	8286(1)	-979(1)	20(1)
O(3)	1753(1)	9423(1)	1632(1)	22(1)
N(1)	5465(1)	8085(1)	4666(1)	16(1)
N(2)	4274(1)	7125(1)	4558(1)	16(1)
N(3)	3076(1)	6623(1)	3373(1)	16(1)
C(1)	5746(1)	9247(1)	1917(1)	20(1)
C(2)	6817(2)	10104(1)	1682(1)	23(1)
C(3)	8230(2)	10897(1)	2713(1)	24(1)
C(4)	8566(1)	10846(2)	3992(1)	24(1)
C(5)	7506(1)	9994(1)	4234(1)	20(1)
C(6)	6084(1)	9175(1)	3193(1)	16(1)
C(7)	5017(1)	8226(1)	3464(1)	15(1)
C(8)	3523(1)	7306(1)	2659(1)	15(1)
C(9)	2462(1)	6963(1)	1254(1)	15(1)
C(10)	1298(1)	4911(1)	-865(1)	16(1)
C(11)	365(1)	5813(1)	-1382(1)	17(1)
C(12)	453(1)	7401(1)	-582(1)	16(1)
C(13)	1606(1)	7950(1)	798(1)	16(1)
C(14)	1354(1)	3267(1)	-1617(1)	21(1)
C(15)	4325(1)	6709(1)	5667(1)	16(1)
C(16)	5687(1)	7229(1)	6826(1)	18(1)
C(17)	5785(1)	6880(1)	7943(1)	21(1)
C(18)	4501(1)	6005(1)	7882(1)	19(1)
C(19)	3140(1)	5449(2)	6751(1)	23(1)
C(20)	3067(1)	5820(2)	5655(1)	22(1)

Table 3. Bond lengths [Å] and angles [°] for **4g**.

F(1)-C(18)	1.3541(12)
F(2)-C(20)	1.3441(14)
O(1)-C(9)	1.3710(13)
O(1)-C(10)	1.3503(13)
O(2)-C(12)	1.2455(14)
O(3)-H(3A)	0.83(2)
O(3)-C(13)	1.3454(14)
N(1)-N(2)	1.3358(13)
N(1)-C(7)	1.3386(14)
N(2)-N(3)	1.3311(13)
N(2)-C(15)	1.4248(14)
N(3)-C(8)	1.3373(14)
C(1)-H(1)	0.9500
C(1)-C(2)	1.3928(16)
C(1)-C(6)	1.3969(16)
C(2)-H(2)	0.9500
C(2)-C(3)	1.3860(18)
C(3)-H(3B)	0.9500
C(3)-C(4)	1.3915(18)
C(4)-H(4)	0.9500
C(4)-C(5)	1.3882(16)
C(5)-H(5)	0.9500
C(5)-C(6)	1.4005(16)
C(6)-C(7)	1.4726(15)
C(7)-C(8)	1.4126(15)
C(8)-C(9)	1.4727(14)
C(9)-C(13)	1.3557(15)
C(10)-C(11)	1.3550(16)
C(10)-C(14)	1.4880(16)
C(11)-H(15)	0.9500
C(11)-C(12)	1.4300(16)
C(12)-C(13)	1.4588(15)
C(14)-H(26A)	0.9800
C(14)-H(26B)	0.9800
C(14)-H(26C)	0.9800
C(15)-C(16)	1.3922(15)
C(15)-C(20)	1.3904(16)
C(16)-H(19)	0.9500
C(16)-C(17)	1.3857(15)
C(17)-H(20)	0.9500
C(17)-C(18)	1.3766(17)
C(18)-C(19)	1.3759(17)
C(19)-H(22)	0.9500
C(19)-C(20)	1.3810(16)
C(10)-O(1)-C(9)	119.91(9)
C(13)-O(3)-H(3A)	108.8(13)
N(2)-N(1)-C(7)	104.27(9)
N(1)-N(2)-C(15)	120.47(9)
N(3)-N(2)-N(1)	115.51(9)

N(3)-N(2)-C(15)	124.02(9)
N(2)-N(3)-C(8)	103.49(9)
C(2)-C(1)-H(1)	119.8
C(2)-C(1)-C(6)	120.40(11)
C(6)-C(1)-H(1)	119.8
C(1)-C(2)-H(2)	119.8
C(3)-C(2)-C(1)	120.42(11)
C(3)-C(2)-H(2)	119.8
C(2)-C(3)-H(3B)	120.2
C(2)-C(3)-C(4)	119.50(11)
C(4)-C(3)-H(3B)	120.2
C(3)-C(4)-H(4)	119.8
C(5)-C(4)-C(3)	120.46(11)
C(5)-C(4)-H(4)	119.8
C(4)-C(5)-H(5)	119.8
C(4)-C(5)-C(6)	120.36(11)
C(6)-C(5)-H(5)	119.8
C(1)-C(6)-C(5)	118.86(10)
C(1)-C(6)-C(7)	122.02(10)
C(5)-C(6)-C(7)	119.09(10)
N(1)-C(7)-C(6)	119.62(10)
N(1)-C(7)-C(8)	107.58(9)
C(8)-C(7)-C(6)	132.75(10)
N(3)-C(8)-C(7)	109.15(9)
N(3)-C(8)-C(9)	117.86(10)
C(7)-C(8)-C(9)	132.97(10)
O(1)-C(9)-C(8)	112.13(9)
C(13)-C(9)-O(1)	121.63(10)
C(13)-C(9)-C(8)	126.09(10)
O(1)-C(10)-C(11)	121.96(10)
O(1)-C(10)-C(14)	112.33(9)
C(11)-C(10)-C(14)	125.70(10)
C(10)-C(11)-H(15)	119.4
C(10)-C(11)-C(12)	121.10(10)
C(12)-C(11)-H(15)	119.4
O(2)-C(12)-C(11)	124.55(10)
O(2)-C(12)-C(13)	120.24(10)
C(11)-C(12)-C(13)	115.18(10)
O(3)-C(13)-C(9)	120.41(10)
O(3)-C(13)-C(12)	119.34(10)
C(9)-C(13)-C(12)	120.16(10)
C(10)-C(14)-H(26A)	109.5
C(10)-C(14)-H(26B)	109.5
C(10)-C(14)-H(26C)	109.5
H(26A)-C(14)-H(26B)	109.5
H(26A)-C(14)-H(26C)	109.5
H(26B)-C(14)-H(26C)	109.5
C(16)-C(15)-N(2)	118.76(10)
C(20)-C(15)-N(2)	123.00(10)
C(20)-C(15)-C(16)	118.24(10)
C(15)-C(16)-H(19)	119.5
C(17)-C(16)-C(15)	121.00(11)
C(17)-C(16)-H(19)	119.5
C(16)-C(17)-H(20)	120.8

C(18)-C(17)-C(16)	118.46(11)
C(18)-C(17)-H(20)	120.8
F(1)-C(18)-C(17)	119.42(10)
F(1)-C(18)-C(19)	118.04(10)
C(19)-C(18)-C(17)	122.54(11)
C(18)-C(19)-H(22)	121.0
C(18)-C(19)-C(20)	117.93(11)
C(20)-C(19)-H(22)	121.0
F(2)-C(20)-C(15)	121.49(10)
F(2)-C(20)-C(19)	116.68(11)
C(19)-C(20)-C(15)	121.82(11)

Symmetry transformations used to generate equivalent atoms

Table 4. Anisotropic displacement parameters ($Å^2$x 10^3) for **4g**. The anisotropic displacement factor exponent takes the form: $-2\pi^2[h^2 a^{*2}U^{11} + ... + 2 h k a^* b^* U^{12}]$

	U^{11}	U^{22}	U^{33}	U^{23}	U^{13}	U^{12}
F(1)	24(1)	41(1)	20(1)	21(1)	7(1)	6(1)
F(2)	28(1)	79(1)	20(1)	26(1)	-7(1)	-26(1)
O(1)	17(1)	17(1)	13(1)	6(1)	4(1)	4(1)
O(2)	20(1)	20(1)	19(1)	10(1)	2(1)	4(1)
O(3)	24(1)	16(1)	17(1)	5(1)	-1(1)	5(1)
N(1)	16(1)	18(1)	16(1)	7(1)	5(1)	1(1)
N(2)	15(1)	18(1)	12(1)	6(1)	3(1)	1(1)
N(3)	17(1)	18(1)	12(1)	6(1)	3(1)	3(1)
C(1)	19(1)	24(1)	19(1)	11(1)	6(1)	5(1)
C(2)	26(1)	27(1)	23(1)	15(1)	12(1)	9(1)
C(3)	23(1)	25(1)	32(1)	16(1)	14(1)	5(1)
C(4)	19(1)	26(1)	27(1)	11(1)	6(1)	-1(1)
C(5)	20(1)	23(1)	19(1)	9(1)	6(1)	3(1)
C(6)	17(1)	16(1)	18(1)	7(1)	7(1)	6(1)
C(7)	16(1)	15(1)	13(1)	5(1)	5(1)	5(1)
C(8)	16(1)	15(1)	14(1)	6(1)	5(1)	5(1)
C(9)	15(1)	17(1)	13(1)	5(1)	4(1)	1(1)
C(10)	16(1)	20(1)	13(1)	5(1)	6(1)	1(1)
C(11)	16(1)	21(1)	12(1)	6(1)	3(1)	1(1)
C(12)	14(1)	20(1)	14(1)	9(1)	5(1)	1(1)
C(13)	16(1)	17(1)	14(1)	6(1)	4(1)	1(1)
C(14)	24(1)	19(1)	18(1)	5(1)	8(1)	4(1)
C(15)	19(1)	16(1)	13(1)	6(1)	6(1)	4(1)
C(16)	17(1)	20(1)	17(1)	9(1)	4(1)	2(1)
C(17)	19(1)	27(1)	16(1)	11(1)	2(1)	4(1)
C(18)	22(1)	24(1)	16(1)	12(1)	8(1)	8(1)
C(19)	22(1)	29(1)	20(1)	11(1)	7(1)	-2(1)
C(20)	19(1)	30(1)	14(1)	8(1)	1(1)	-3(1)

Table 5. Hydrogen coordinates (x 10^4) and isotropic displacement parameters (Å^{2}x 10^3)

for **4g**.

	x	y	z	U(eq)
H(3A)	1180(20)	9880(20)	1200(20)	44(5)
H(1)	4780	8708	1204	24
H(2)	6577	10145	810	27
H(3B)	8964	11471	2547	29
H(4)	9528	11399	4704	29
H(5)	7747	9966	5111	24
H(15)	-361	5383	-2291	20
H(26A)	2355	3175	-1788	31
H(26B)	464	2871	-2485	31
H(26C)	1280	2673	-1075	31
H(19)	6562	7832	6852	22
H(20)	6716	7237	8732	25
H(22)	2278	4829	6726	28

Table 6. Torsion angles [°] for **4g**.

F(1)-C(18)-C(19)-C(20)	178.20(11)
O(1)-C(9)-C(13)-O(3)	179.38(9)
O(1)-C(9)-C(13)-C(12)	2.77(16)
O(1)-C(10)-C(11)-C(12)	1.45(16)
O(2)-C(12)-C(13)-O(3)	-0.62(16)
O(2)-C(12)-C(13)-C(9)	176.02(10)
N(1)-N(2)-N(3)-C(8)	0.10(12)
N(1)-N(2)-C(15)-C(16)	-4.75(15)
N(1)-N(2)-C(15)-C(20)	174.44(11)
N(1)-C(7)-C(8)-N(3)	0.15(12)
N(1)-C(7)-C(8)-C(9)	-178.15(11)
N(2)-N(1)-C(7)-C(6)	-177.87(9)
N(2)-N(1)-C(7)-C(8)	-0.09(11)
N(2)-N(3)-C(8)-C(7)	-0.15(11)
N(2)-N(3)-C(8)-C(9)	178.44(9)
N(2)-C(15)-C(16)-C(17)	178.80(10)
N(2)-C(15)-C(20)-F(2)	-0.35(19)
N(2)-C(15)-C(20)-C(19)	-179.19(11)
N(3)-N(2)-C(15)-C(16)	175.97(10)
N(3)-N(2)-C(15)-C(20)	-4.85(17)
N(3)-C(8)-C(9)-O(1)	-64.85(12)
N(3)-C(8)-C(9)-C(13)	110.68(13)
C(1)-C(2)-C(3)-C(4)	-0.72(19)
C(1)-C(6)-C(7)-N(1)	173.82(10)
C(1)-C(6)-C(7)-C(8)	-3.30(19)
C(2)-C(1)-C(6)-C(5)	0.78(17)
C(2)-C(1)-C(6)-C(7)	-177.16(11)
C(2)-C(3)-C(4)-C(5)	0.78(19)
C(3)-C(4)-C(5)-C(6)	-0.05(19)
C(4)-C(5)-C(6)-C(1)	-0.72(17)
C(4)-C(5)-C(6)-C(7)	177.28(11)
C(5)-C(6)-C(7)-N(1)	-4.11(16)
C(5)-C(6)-C(7)-C(8)	178.77(11)
C(6)-C(1)-C(2)-C(3)	-0.06(18)
C(6)-C(7)-C(8)-N(3)	177.53(11)
C(6)-C(7)-C(8)-C(9)	-0.8(2)
C(7)-N(1)-N(2)-N(3)	-0.01(12)
C(7)-N(1)-N(2)-C(15)	-179.35(9)
C(7)-C(8)-C(9)-O(1)	113.34(13)
C(7)-C(8)-C(9)-C(13)	-71.14(17)
C(8)-C(9)-C(13)-O(3)	4.25(17)
C(8)-C(9)-C(13)-C(12)	-172.36(10)
C(9)-O(1)-C(10)-C(11)	-1.01(15)
C(9)-O(1)-C(10)-C(14)	177.94(9)
C(10)-O(1)-C(9)-C(8)	174.60(9)
C(10)-O(1)-C(9)-C(13)	-1.15(15)
C(10)-C(11)-C(12)-O(2)	-177.99(11)
C(10)-C(11)-C(12)-C(13)	0.16(15)
C(11)-C(12)-C(13)-O(3)	-178.86(9)
C(11)-C(12)-C(13)-C(9)	-2.22(15)

C(14)-C(10)-C(11)-C(12)	-177.36(10)
C(15)-N(2)-N(3)-C(8)	179.41(10)
C(15)-C(16)-C(17)-C(18)	-0.03(18)
C(16)-C(15)-C(20)-F(2)	178.84(11)
C(16)-C(15)-C(20)-C(19)	0.00(19)
C(16)-C(17)-C(18)-F(1)	-178.60(10)
C(16)-C(17)-C(18)-C(19)	0.96(18)
C(17)-C(18)-C(19)-C(20)	-1.36(19)
C(18)-C(19)-C(20)-F(2)	-178.03(12)
C(18)-C(19)-C(20)-C(15)	0.86(19)
C(20)-C(15)-C(16)-C(17)	-0.43(17)

Symmetry transformations used to generate equivalent atoms

Table 7. Hydrogen bonds for **4g** [Å and °]

D-H...A	d(D-H)	d(H...A)	d(D...A)	<(DHA)
O(3)-H(3A)...O(2)#1	0.83(2)	1.94(2)	2.7128(12)	153.2(18)

Symmetry transformations used to generate equivalent atoms:
#1 -x,-y+2,-z

Crystallographic data for *(E)-3-(hydrazineylidene(phenyl)methyl)-5-(5-methyl-1H-pyrazol-3-yl)isoxazol-4-ol* **6a**

Table 8. Crystal data and structure refinement for **6a**.

Identification code	**2343877**	
Empirical formula	$C_{14}H_{13}N_5O_2$	
Formula weight	283.29	
Temperature	100.00(10) K	
Wavelength	1.54184 Å	
Crystal system	Monoclinic	
Space group	$P2_1/n$	
Unit cell dimensions	a = 15.35343(12) Å	□= 90°.
	b = 11.17807(8) Å	□= 113.3476(10)°.
	c = 17.13289(15) Å	□ = 90°.
Volume	2699.61(4) $Å^3$	
Z	8	
Density (calculated)	1.394 g/cm^3	
Absorption coefficient	0.812 mm^{-1}	
F(000)	1184	
Crystal size	0.31 x 0.1 x 0.05 mm^3	
Theta range for data collection	3.277 to 80.004°.	
Index ranges	-19<=h<=14, -14<=k<=14, -21<=l<=21	
Reflections collected	43095	
Independent reflections	5878 [R(int) = 0.0314]	
Observed reflections	5467	
Completeness to theta = 67.684°	100.0 %	
Absorption correction	Gaussian	
Max. and min. transmission	1.000 and 0.429	
Refinement method	Full-matrix least-squares on F^2	
Data / restraints / parameters	5878 / 8 / 426	
Goodness-of-fit on F^2	1.061	
Final R indices [I>2sigma(I)]	R1 = 0.0364, wR2 = 0.0956	
R indices (all data)	R1 = 0.0387, wR2 = 0.0973	
Extinction coefficient	n/a	
Largest diff. peak and hole	0.332 and -0.227 e.$Å^{-3}$	

Table 9. Atomic coordinates (x 10^4) and equivalent isotropic displacement parameters ($Å^2$x 10^3) for **6a**. U(eq) is defined as one third of the trace of the orthogonalized U^{ij} tensor.

	x	y	z	U(eq)
O(1A)	5809(1)	11568(1)	7785(1)	25(1)
O(2A)	4799(1)	10282(1)	9118(1)	27(1)
N(1A)	3138(1)	13374(1)	7699(1)	20(1)
N(2A)	3770(1)	12548(1)	8173(1)	20(1)
N(3A)	6480(1)	10670(1)	8183(1)	24(1)
N(4A)	6254(1)	8742(1)	9732(1)	20(1)
N(5A)	6607(1)	7813(1)	10265(1)	23(1)
C(1A)	2661(1)	14610(1)	6386(1)	33(1)
C(2A)	3297(1)	13739(1)	7015(1)	23(1)
C(3A)	4088(1)	13125(1)	7041(1)	23(1)
C(4A)	4353(1)	12395(1)	7768(1)	19(1)
C(5A)	5114(1)	11528(1)	8094(1)	20(1)
C(6A)	5310(1)	10631(1)	8674(1)	20(1)
C(7A)	6176(1)	10117(1)	8707(1)	19(1)
C(8A)	6671(1)	9114(1)	9251(1)	18(1)
C(9A)	7561(1)	8588(1)	9244(1)	19(1)
C(10A)	7545(1)	7418(1)	8952(1)	24(1)
C(11A)	8365(1)	6892(1)	8955(1)	26(1)
C(12A)	9208(1)	7525(1)	9255(1)	26(1)
C(13A)	9233(1)	8676(1)	9557(1)	32(1)
C(14A)	8413(1)	9214(1)	9551(1)	26(1)
O(1B)	4002(1)	7758(1)	6873(1)	23(1)
O(2B)	5114(1)	9490(1)	5803(1)	23(1)
N(1B)	6725(1)	6141(1)	6973(1)	21(1)
N(2B)	6217(1)	7147(1)	6659(1)	21(1)
N(3B)	3340(1)	8690(1)	6561(1)	22(1)
N(4B)	3609(1)	10994(1)	5255(1)	21(1)
N(5B)	3276(1)	12030(1)	4826(1)	24(1)
C(1B)	6786(1)	4256(1)	7755(1)	28(1)
C(2B)	6322(1)	5401(1)	7357(1)	21(1)
C(3B)	5492(1)	5950(1)	7298(1)	21(1)
C(4B)	5462(1)	7029(1)	6862(1)	20(1)
C(5B)	4734(1)	7944(1)	6622(1)	20(1)
C(6B)	4572(1)	8976(1)	6167(1)	19(1)
C(7B)	3683(1)	9405(1)	6138(1)	19(1)
C(8B)	3176(1)	10462(1)	5672(1)	19(1)
C(9B)	2261(1)	10827(1)	5706(1)	22(1)
C(10B)	1427(7)	10880(10)	4973(8)	36(1)
C(10C)	1459(11)	11044(10)	4966(12)	36(1)
C(11B)	556(10)	11200(14)	4990(9)	43(2)
C(11C)	659(14)	11474(14)	5060(13)	43(2)
C(12B)	531(11)	11448(13)	5775(9)	39(1)
C(12C)	622(16)	11693(14)	5843(13)	39(1)
C(13B)	1348(7)	11369(8)	6508(11)	31(1)
C(13C)	1411(10)	11483(11)	6577(17)	31(1)
C(14B)	2218(1)	11054(1)	6491(1)	25(1)

Table 10. Bond lengths [Å] and angles [°] for **6a**.

O(1A)-N(3A)	1.4064(12)
O(1A)-C(5A)	1.3664(13)
O(2A)-H(2A)	0.89(2)
O(2A)-C(6A)	1.3488(14)
N(1A)-H(1A)	0.903(16)
N(1A)-N(2A)	1.3523(13)
N(1A)-C(2A)	1.3513(15)
N(2A)-C(4A)	1.3433(14)
N(3A)-C(7A)	1.3180(15)
N(4A)-N(5A)	1.3466(14)
N(4A)-C(8A)	1.2976(14)
N(5A)-H(5A)	0.901(17)
N(5A)-H(5B)	0.885(16)
C(1A)-H(1B)	0.9800
C(1A)-H(1C)	0.9800
C(1A)-H(1D)	0.9800
C(1A)-C(2A)	1.4928(16)
C(2A)-C(3A)	1.3802(16)
C(3A)-H(3A)	0.9500
C(3A)-C(4A)	1.4077(15)
C(4A)-C(5A)	1.4502(15)
C(5A)-C(6A)	1.3581(16)
C(6A)-C(7A)	1.4288(15)
C(7A)-C(8A)	1.4629(15)
C(8A)-C(9A)	1.4924(14)
C(9A)-C(10A)	1.3967(16)
C(9A)-C(14A)	1.3893(16)
C(10A)-H(10A)	0.9500
C(10A)-C(11A)	1.3879(16)
C(11A)-H(11A)	0.9500
C(11A)-C(12A)	1.3827(18)
C(12A)-H(12A)	0.9500
C(12A)-C(13A)	1.3817(18)
C(13A)-H(13A)	0.9500
C(13A)-C(14A)	1.3930(17)
C(14A)-H(14A)	0.9500
O(1B)-N(3B)	1.4052(12)
O(1B)-C(5B)	1.3694(13)
O(2B)-H(2B)	0.88(2)
O(2B)-C(6B)	1.3503(13)
N(1B)-H(1E)	0.896(17)
N(1B)-N(2B)	1.3528(13)
N(1B)-C(2B)	1.3493(15)
N(2B)-C(4B)	1.3424(14)
N(3B)-C(7B)	1.3205(15)
N(4B)-N(5B)	1.3581(14)
N(4B)-C(8B)	1.2976(14)
N(5B)-H(5C)	0.895(17)
N(5B)-H(5D)	0.899(17)
C(1B)-H(1F)	0.9800
C(1B)-H(1G)	0.9800
C(1B)-H(1H)	0.9800

C(1B)-C(2B)	1.4922(16)
C(2B)-C(3B)	1.3804(16)
C(3B)-H(3B)	0.9500
C(3B)-C(4B)	1.4099(16)
C(4B)-C(5B)	1.4493(15)
C(5B)-C(6B)	1.3590(16)
C(6B)-C(7B)	1.4286(15)
C(7B)-C(8B)	1.4653(15)
C(8B)-C(9B)	1.4861(15)
C(9B)-C(10B)	1.395(8)
C(9B)-C(10C)	1.394(12)
C(9B)-C(14B)	1.3954(16)
C(10B)-H(10B)	0.9500
C(10B)-C(11B)	1.395(8)
C(10C)-H(10C)	0.9500
C(10C)-C(11C)	1.387(13)
C(11B)-H(11B)	0.9500
C(11B)-C(12B)	1.388(8)
C(11C)-H(11C)	0.9500
C(11C)-C(12C)	1.387(12)
C(12B)-H(12B)	0.9500
C(12B)-C(13B)	1.382(8)
C(12C)-H(12C)	0.9500
C(12C)-C(13C)	1.377(13)
C(13B)-H(13B)	0.9500
C(13B)-C(14B)	1.394(8)
C(13C)-H(13C)	0.9500
C(13C)-C(14B)	1.391(12)
C(14B)-H(14B)	0.9500
C(14B)-H(2)	0.9500

C(5A)-O(1A)-N(3A)	108.84(8)
C(6A)-O(2A)-H(2A)	103.5(12)
N(2A)-N(1A)-H(1A)	118.1(10)
C(2A)-N(1A)-H(1A)	128.6(10)
C(2A)-N(1A)-N(2A)	113.25(9)
C(4A)-N(2A)-N(1A)	103.99(9)
C(7A)-N(3A)-O(1A)	106.00(9)
C(8A)-N(4A)-N(5A)	120.67(10)
N(4A)-N(5A)-H(5A)	113.4(10)
N(4A)-N(5A)-H(5B)	116.5(10)
H(5A)-N(5A)-H(5B)	118.6(14)
H(1B)-C(1A)-H(1C)	109.5
H(1B)-C(1A)-H(1D)	109.5
H(1C)-C(1A)-H(1D)	109.5
C(2A)-C(1A)-H(1B)	109.5
C(2A)-C(1A)-H(1C)	109.5
C(2A)-C(1A)-H(1D)	109.5
N(1A)-C(2A)-C(1A)	122.11(11)
N(1A)-C(2A)-C(3A)	106.31(10)
C(3A)-C(2A)-C(1A)	131.53(11)
C(2A)-C(3A)-H(3A)	127.5
C(2A)-C(3A)-C(4A)	104.96(10)
C(4A)-C(3A)-H(3A)	127.5

N(2A)-C(4A)-C(3A)	111.49(10)
N(2A)-C(4A)-C(5A)	119.07(10)
C(3A)-C(4A)-C(5A)	129.41(10)
O(1A)-C(5A)-C(4A)	117.52(10)
C(6A)-C(5A)-O(1A)	109.10(9)
C(6A)-C(5A)-C(4A)	133.38(10)
O(2A)-C(6A)-C(5A)	128.00(10)
O(2A)-C(6A)-C(7A)	127.01(10)
C(5A)-C(6A)-C(7A)	104.98(10)
N(3A)-C(7A)-C(6A)	111.07(10)
N(3A)-C(7A)-C(8A)	123.93(10)
C(6A)-C(7A)-C(8A)	125.00(10)
N(4A)-C(8A)-C(7A)	112.83(9)
N(4A)-C(8A)-C(9A)	124.24(10)
C(7A)-C(8A)-C(9A)	122.93(9)
C(10A)-C(9A)-C(8A)	119.03(10)
C(14A)-C(9A)-C(8A)	121.70(10)
C(14A)-C(9A)-C(10A)	119.22(10)
C(9A)-C(10A)-H(10A)	119.8
C(11A)-C(10A)-C(9A)	120.49(11)
C(11A)-C(10A)-H(10A)	119.8
C(10A)-C(11A)-H(11A)	120.0
C(12A)-C(11A)-C(10A)	119.95(11)
C(12A)-C(11A)-H(11A)	120.0
C(11A)-C(12A)-H(12A)	120.0
C(13A)-C(12A)-C(11A)	119.92(11)
C(13A)-C(12A)-H(12A)	120.0
C(12A)-C(13A)-H(13A)	119.7
C(12A)-C(13A)-C(14A)	120.55(12)
C(14A)-C(13A)-H(13A)	119.7
C(9A)-C(14A)-C(13A)	119.86(11)
C(9A)-C(14A)-H(14A)	120.1
C(13A)-C(14A)-H(14A)	120.1
C(5B)-O(1B)-N(3B)	109.17(8)
C(6B)-O(2B)-H(2B)	105.2(12)
N(2B)-N(1B)-H(1E)	119.3(11)
C(2B)-N(1B)-H(1E)	127.1(10)
C(2B)-N(1B)-N(2B)	113.61(9)
C(4B)-N(2B)-N(1B)	103.60(9)
C(7B)-N(3B)-O(1B)	105.75(8)
C(8B)-N(4B)-N(5B)	121.56(10)
N(4B)-N(5B)-H(5C)	111.6(11)
N(4B)-N(5B)-H(5D)	116.0(10)
H(5C)-N(5B)-H(5D)	117.1(14)
H(1F)-C(1B)-H(1G)	109.5
H(1F)-C(1B)-H(1H)	109.5
H(1G)-C(1B)-H(1H)	109.5
C(2B)-C(1B)-H(1F)	109.5
C(2B)-C(1B)-H(1G)	109.5
C(2B)-C(1B)-H(1H)	109.5
N(1B)-C(2B)-C(1B)	121.31(10)
N(1B)-C(2B)-C(3B)	106.27(10)
C(3B)-C(2B)-C(1B)	132.41(11)
C(2B)-C(3B)-H(3B)	127.6

C(2B)-C(3B)-C(4B)	104.73(10)
C(4B)-C(3B)-H(3B)	127.6
N(2B)-C(4B)-C(3B)	111.80(10)
N(2B)-C(4B)-C(5B)	120.28(10)
C(3B)-C(4B)-C(5B)	127.91(10)
O(1B)-C(5B)-C(4B)	116.08(10)
C(6B)-C(5B)-O(1B)	108.80(9)
C(6B)-C(5B)-C(4B)	135.09(10)
O(2B)-C(6B)-C(5B)	128.24(10)
O(2B)-C(6B)-C(7B)	126.66(10)
C(5B)-C(6B)-C(7B)	105.09(10)
N(3B)-C(7B)-C(6B)	111.19(10)
N(3B)-C(7B)-C(8B)	123.09(10)
C(6B)-C(7B)-C(8B)	125.65(10)
N(4B)-C(8B)-C(7B)	113.12(10)
N(4B)-C(8B)-C(9B)	126.46(10)
C(7B)-C(8B)-C(9B)	120.42(10)
C(10B)-C(9B)-C(8B)	121.4(7)
C(10B)-C(9B)-C(14B)	118.9(7)
C(10C)-C(9B)-C(8B)	121.3(11)
C(10C)-C(9B)-C(14B)	118.9(11)
C(14B)-C(9B)-C(8B)	119.58(10)
C(9B)-C(10B)-H(10B)	118.8
C(9B)-C(10B)-C(11B)	122.5(13)
C(11B)-C(10B)-H(10B)	118.8
C(9B)-C(10C)-H(10C)	121.4
C(11C)-C(10C)-C(9B)	117(2)
C(11C)-C(10C)-H(10C)	121.4
C(10B)-C(11B)-H(11B)	121.1
C(12B)-C(11B)-C(10B)	117.8(13)
C(12B)-C(11B)-H(11B)	121.1
C(10C)-C(11C)-H(11C)	118.2
C(10C)-C(11C)-C(12C)	124(2)
C(12C)-C(11C)-H(11C)	118.2
C(11B)-C(12B)-H(12B)	119.8
C(13B)-C(12B)-C(11B)	120.3(14)
C(13B)-C(12B)-H(12B)	119.8
C(11C)-C(12C)-H(12C)	120.2
C(13C)-C(12C)-C(11C)	120(2)
C(13C)-C(12C)-H(12C)	120.2
C(12B)-C(13B)-H(13B)	119.0
C(12B)-C(13B)-C(14B)	121.9(14)
C(14B)-C(13B)-H(13B)	119.0
C(12C)-C(13C)-H(13C)	121.3
C(12C)-C(13C)-C(14B)	117(2)
C(14B)-C(13C)-H(13C)	121.3
C(9B)-C(14B)-H(14B)	120.7
C(9B)-C(14B)-H(2)	118.3
C(13B)-C(14B)-C(9B)	118.6(8)
C(13B)-C(14B)-H(14B)	120.7
C(13C)-C(14B)-C(9B)	123.3(11)
C(13C)-C(14B)-H(2)	118.3

Symmetry transformations used to generate equivalent atoms

Table 11. Anisotropic displacement parameters ($Å^2$x 10^3) for **6a**. The anisotropic displacement factor exponent takes the form: $-2\pi^2[h^2 a^{*2}U^{11} + ... + 2 h k a^* b^* U^{12}]$

	U^{11}	U^{22}	U^{33}	U^{23}	U^{13}	U^{12}
O(1A)	22(1)	25(1)	32(1)	10(1)	16(1)	7(1)
O(2A)	25(1)	33(1)	30(1)	12(1)	18(1)	9(1)
N(1A)	20(1)	22(1)	20(1)	3(1)	9(1)	4(1)
N(2A)	19(1)	21(1)	19(1)	2(1)	8(1)	3(1)
N(3A)	22(1)	23(1)	30(1)	9(1)	14(1)	7(1)
N(4A)	20(1)	20(1)	18(1)	0(1)	7(1)	0(1)
N(5A)	24(1)	25(1)	22(1)	6(1)	10(1)	3(1)
C(1A)	34(1)	38(1)	30(1)	14(1)	16(1)	14(1)
C(2A)	24(1)	24(1)	21(1)	4(1)	10(1)	3(1)
C(3A)	24(1)	25(1)	22(1)	4(1)	12(1)	3(1)
C(4A)	19(1)	19(1)	20(1)	1(1)	8(1)	0(1)
C(5A)	19(1)	22(1)	22(1)	0(1)	10(1)	0(1)
C(6A)	19(1)	22(1)	21(1)	1(1)	10(1)	1(1)
C(7A)	18(1)	20(1)	20(1)	0(1)	8(1)	0(1)
C(8A)	18(1)	19(1)	18(1)	-1(1)	7(1)	0(1)
C(9A)	19(1)	21(1)	17(1)	3(1)	8(1)	3(1)
C(10A)	22(1)	23(1)	25(1)	-3(1)	9(1)	-1(1)
C(11A)	29(1)	21(1)	28(1)	-2(1)	12(1)	4(1)
C(12A)	23(1)	26(1)	32(1)	3(1)	13(1)	6(1)
C(13A)	21(1)	29(1)	48(1)	-5(1)	14(1)	-3(1)
C(14A)	23(1)	21(1)	35(1)	-4(1)	12(1)	-1(1)
O(1B)	21(1)	23(1)	28(1)	7(1)	14(1)	4(1)
O(2B)	23(1)	24(1)	28(1)	5(1)	16(1)	1(1)
N(1B)	21(1)	21(1)	24(1)	1(1)	11(1)	2(1)
N(2B)	21(1)	20(1)	23(1)	2(1)	11(1)	1(1)
N(3B)	20(1)	23(1)	24(1)	4(1)	11(1)	3(1)
N(4B)	23(1)	21(1)	19(1)	1(1)	10(1)	-1(1)
N(5B)	27(1)	25(1)	24(1)	6(1)	13(1)	2(1)
C(1B)	32(1)	23(1)	32(1)	5(1)	16(1)	5(1)
C(2B)	24(1)	20(1)	21(1)	-1(1)	11(1)	-1(1)
C(3B)	22(1)	22(1)	22(1)	0(1)	12(1)	-1(1)
C(4B)	20(1)	21(1)	20(1)	-1(1)	9(1)	-1(1)
C(5B)	19(1)	23(1)	21(1)	0(1)	11(1)	-1(1)
C(6B)	19(1)	22(1)	20(1)	-1(1)	10(1)	-2(1)
C(7B)	19(1)	20(1)	18(1)	-2(1)	9(1)	-2(1)
C(8B)	20(1)	21(1)	18(1)	-1(1)	8(1)	-1(1)
C(9B)	21(1)	22(1)	24(1)	4(1)	11(1)	2(1)
C(10B)	26(1)	57(2)	26(1)	16(2)	11(1)	4(1)
C(10C)	26(1)	57(2)	26(1)	16(2)	11(1)	4(1)
C(11B)	21(3)	64(5)	41(2)	26(3)	9(2)	7(3)
C(11C)	21(3)	64(5)	41(2)	26(3)	9(2)	7(3)
C(12B)	31(3)	34(4)	61(2)	19(3)	29(2)	12(3)
C(12C)	31(3)	34(4)	61(2)	19(3)	29(2)	12(3)
C(13B)	38(1)	23(1)	43(3)	-5(2)	27(2)	0(1)
C(13C)	38(1)	23(1)	43(3)	-5(2)	27(2)	0(1)
C(14B)	27(1)	23(1)	27(1)	-4(1)	14(1)	-3(1)

Table 12. Hydrogen coordinates (x 10^4) and isotropic displacement parameters ($Å^2$ x 10^3) for **6a**.

	x	y	z	U(eq)
H(2A)	5133(13)	9675(18)	9430(12)	46(5)
H(1A)	2671(11)	13597(15)	7862(10)	29(4)
H(5A)	6405(11)	7789(15)	10692(11)	30(4)
H(5B)	7201(11)	7610(14)	10374(10)	28(4)
H(1B)	2232	14975	6615	50
H(1C)	2288	14191	5855	50
H(1D)	3044	15234	6273	50
H(3A)	4389	13183	6652	27
H(10A)	6968	6979	8751	29
H(11A)	8347	6098	8751	31
H(12A)	9769	7168	9253	32
H(13A)	9816	9104	9770	39
H(14A)	8435	10008	9756	32
H(2B)	4787(13)	10113(19)	5525(12)	47(5)
H(1E)	7273(12)	6030(15)	6913(10)	30(4)
H(5C)	3489(11)	12142(15)	4415(11)	31(4)
H(5D)	2660(12)	12191(15)	4697(10)	29(4)
H(1F)	7102	3902	7413	42
H(1G)	6306	3700	7782	42
H(1H)	7256	4413	8331	42
H(3B)	5041	5663	7505	26
H(10B)	1453	10690	4442	43
H(10C)	1461	10903	4420	43
H(11B)	-1	11246	4482	52
H(11C)	108	11628	4562	52
H(12B)	-50	11673	5808	46
H(12C)	55	11985	5873	46
H(13B)	1315	11534	7040	38
H(13C)	1404	11627	7121	38
H(14B)	2771	10995	7003	30
H(2)	2769	10907	6992	30

Table 13. Torsion angles [°] for **6a**.

O(1A)-N(3A)-C(7A)-C(6A)	-0.27(13)
O(1A)-N(3A)-C(7A)-C(8A)	-179.99(10)
O(1A)-C(5A)-C(6A)-O(2A)	-179.04(11)
O(1A)-C(5A)-C(6A)-C(7A)	0.21(13)
O(2A)-C(6A)-C(7A)-N(3A)	179.31(11)
O(2A)-C(6A)-C(7A)-C(8A)	-0.98(19)
N(1A)-N(2A)-C(4A)-C(3A)	0.12(13)
N(1A)-N(2A)-C(4A)-C(5A)	-178.16(10)
N(1A)-C(2A)-C(3A)-C(4A)	0.50(13)
N(2A)-N(1A)-C(2A)-C(1A)	177.43(11)
N(2A)-N(1A)-C(2A)-C(3A)	-0.47(14)
N(2A)-C(4A)-C(5A)-O(1A)	-166.47(10)
N(2A)-C(4A)-C(5A)-C(6A)	13.34(19)
N(3A)-O(1A)-C(5A)-C(4A)	179.47(9)
N(3A)-O(1A)-C(5A)-C(6A)	-0.38(13)
N(3A)-C(7A)-C(8A)-N(4A)	177.89(11)
N(3A)-C(7A)-C(8A)-C(9A)	-2.09(17)
N(4A)-C(8A)-C(9A)-C(10A)	66.38(15)
N(4A)-C(8A)-C(9A)-C(14A)	-111.12(13)
N(5A)-N(4A)-C(8A)-C(7A)	179.08(9)
N(5A)-N(4A)-C(8A)-C(9A)	-0.95(16)
C(1A)-C(2A)-C(3A)-C(4A)	-177.13(13)
C(2A)-N(1A)-N(2A)-C(4A)	0.22(13)
C(2A)-C(3A)-C(4A)-N(2A)	-0.40(14)
C(2A)-C(3A)-C(4A)-C(5A)	177.66(11)
C(3A)-C(4A)-C(5A)-O(1A)	15.60(18)
C(3A)-C(4A)-C(5A)-C(6A)	-164.59(13)
C(4A)-C(5A)-C(6A)-O(2A)	1.1(2)
C(4A)-C(5A)-C(6A)-C(7A)	-179.61(12)
C(5A)-O(1A)-N(3A)-C(7A)	0.40(12)
C(5A)-C(6A)-C(7A)-N(3A)	0.05(13)
C(5A)-C(6A)-C(7A)-C(8A)	179.76(10)
C(6A)-C(7A)-C(8A)-N(4A)	-1.79(16)
C(6A)-C(7A)-C(8A)-C(9A)	178.23(10)
C(7A)-C(8A)-C(9A)-C(10A)	-113.65(12)
C(7A)-C(8A)-C(9A)-C(14A)	68.85(15)
C(8A)-C(9A)-C(10A)-C(11A)	-178.63(10)
C(8A)-C(9A)-C(14A)-C(13A)	178.09(12)
C(9A)-C(10A)-C(11A)-C(12A)	0.53(18)
C(10A)-C(9A)-C(14A)-C(13A)	0.59(18)
C(10A)-C(11A)-C(12A)-C(13A)	0.49(19)
C(11A)-C(12A)-C(13A)-C(14A)	-1.0(2)
C(12A)-C(13A)-C(14A)-C(9A)	0.4(2)
C(14A)-C(9A)-C(10A)-C(11A)	-1.06(17)
O(1B)-N(3B)-C(7B)-C(6B)	-0.66(12)
O(1B)-N(3B)-C(7B)-C(8B)	176.67(9)
O(1B)-C(5B)-C(6B)-O(2B)	179.96(10)
O(1B)-C(5B)-C(6B)-C(7B)	-1.10(12)
O(2B)-C(6B)-C(7B)-N(3B)	-179.92(10)
O(2B)-C(6B)-C(7B)-C(8B)	2.84(18)
N(1B)-N(2B)-C(4B)-C(3B)	-0.35(12)

N(1B)-N(2B)-C(4B)-C(5B)	-179.45(10)
N(1B)-C(2B)-C(3B)-C(4B)	-0.08(12)
N(2B)-N(1B)-C(2B)-C(1B)	-179.01(10)
N(2B)-N(1B)-C(2B)-C(3B)	-0.14(13)
N(2B)-C(4B)-C(5B)-O(1B)	-178.65(10)
N(2B)-C(4B)-C(5B)-C(6B)	3.7(2)
N(3B)-O(1B)-C(5B)-C(4B)	-177.49(9)
N(3B)-O(1B)-C(5B)-C(6B)	0.75(12)
N(3B)-C(7B)-C(8B)-N(4B)	-175.69(10)
N(3B)-C(7B)-C(8B)-C(9B)	4.16(16)
N(4B)-C(8B)-C(9B)-C(10B)	60.5(5)
N(4B)-C(8B)-C(9B)-C(10C)	51.3(4)
N(4B)-C(8B)-C(9B)-C(14B)	-123.39(13)
N(5B)-N(4B)-C(8B)-C(7B)	-176.04(9)
N(5B)-N(4B)-C(8B)-C(9B)	4.12(17)
C(1B)-C(2B)-C(3B)-C(4B)	178.62(12)
C(2B)-N(1B)-N(2B)-C(4B)	0.30(12)
C(2B)-C(3B)-C(4B)-N(2B)	0.27(13)
C(2B)-C(3B)-C(4B)-C(5B)	179.29(11)
C(3B)-C(4B)-C(5B)-O(1B)	2.41(17)
C(3B)-C(4B)-C(5B)-C(6B)	-175.23(12)
C(4B)-C(5B)-C(6B)-O(2B)	-2.3(2)
C(4B)-C(5B)-C(6B)-C(7B)	176.67(12)
C(5B)-O(1B)-N(3B)-C(7B)	-0.04(12)
C(5B)-C(6B)-C(7B)-N(3B)	1.11(13)
C(5B)-C(6B)-C(7B)-C(8B)	-176.13(10)
C(6B)-C(7B)-C(8B)-N(4B)	1.25(16)
C(6B)-C(7B)-C(8B)-C(9B)	-178.91(10)
C(7B)-C(8B)-C(9B)-C(10B)	-119.3(5)
C(7B)-C(8B)-C(9B)-C(10C)	-128.5(4)
C(7B)-C(8B)-C(9B)-C(14B)	56.79(15)
C(8B)-C(9B)-C(10B)-C(11B)	178.9(4)
C(8B)-C(9B)-C(10C)-C(11C)	-174.9(4)
C(8B)-C(9B)-C(14B)-C(13B)	-178.6(4)
C(8B)-C(9B)-C(14B)-C(13C)	175.1(6)
C(9B)-C(10B)-C(11B)-C(12B)	-1.4(5)
C(9B)-C(10C)-C(11C)-C(12C)	-0.1(3)
C(10B)-C(9B)-C(14B)-C(13B)	-2.4(5)
C(10B)-C(11B)-C(12B)-C(13B)	-0.4(6)
C(10C)-C(9B)-C(14B)-C(13C)	0.3(6)
C(10C)-C(11C)-C(12C)-C(13C)	0.3(6)
C(11B)-C(12B)-C(13B)-C(14B)	0.7(7)
C(11C)-C(12C)-C(13C)-C(14B)	-0.1(8)
C(12B)-C(13B)-C(14B)-C(9B)	0.8(7)
C(12C)-C(13C)-C(14B)-C(9B)	-0.2(9)
C(14B)-C(9B)-C(10B)-C(11B)	2.8(5)
C(14B)-C(9B)-C(10C)-C(11C)	-0.1(3)

Symmetry transformations used to generate equivalent atoms

Table 14. Hydrogen bonds for **6a** [Å and °]

D-H...A	d(D-H)	d(H...A)	d(D...A)	<(DHA)
O(2A)-H(2A)...O(2A)#1	0.89(2)	2.449(19)	2.9055(17)	112.3(15)
O(2A)-H(2A)...N(4A)	0.89(2)	1.90(2)	2.6829(13)	145.7(17)
N(1A)-H(1A)...N(3B)#2	0.903(16)	2.147(16)	3.0275(13)	164.7(14)
N(5A)-H(5AA)...N(2A)#1	0.901(17)	2.100(17)	2.9816(14)	165.6(14)
N(5A)-H(5AB)...N(3B)#3	0.885(16)	2.548(16)	3.1862(14)	129.7(13)
O(2B)-H(2B)...O(2B)#4	0.88(2)	2.38(2)	2.8683(17)	115.3(15)
O(2B)-H(2B)...N(4B)	0.88(2)	1.95(2)	2.7074(13)	143.6(17)
N(1B)-H(1B)...N(3A)#5	0.896(17)	2.026(17)	2.9211(14)	176.1(15)
N(5B)-H(5BA)...N(2B)#4	0.895(17)	2.209(17)	3.0817(14)	164.7(15)

Symmetry transformations used to generate equivalent atoms:
#1 -x+1,-y+2,-z+2 #2 -x+1/2,y+1/2,-z+3/2 #3 x+1/2,-y+3/2,z+1/2
#4 -x+1,-y+2,-z+1 #5 -x+3/2,y-1/2,-z+3/2
